# Design, synthesis and biological evaluation of 2,5-diaryloxazolo[4,5-*d*]pyrimidin-7-ylamines as selective cytotoxic agents against HeLa cells

**DOI:** 10.3762/bjoc.22.27

**Published:** 2026-03-03

**Authors:** Maryna V Kachaeva, Agnieszka B Olejniczak, Marta Denel-Bobrowska, Victor V Zhirnov, Yevheniia S Velihina, Stepan G Pilyo, Volodymyr S Brovarets

**Affiliations:** 1 Department of Chemistry of Bioactive Nitrogen-Containing Heterocyclic Bases, V.P. Kukhar Institute of Bioorganic Chemistry and Petrochemistry, National Academy of Science of Ukraine, 1, Academician Kukhar str., Kyiv, 02094, Ukraine; 2 Screening Laboratory, Institute of Medical Biology, Polish Academy of Sciences, 106 Lodowa St. 93-232 Łódź, Poland; 3 Chimie Hétérocyclique pour l’Innovation en Thérapeutique et Imagerie TEP, Institut de Chimie Organique et Analytique, Université d’Orléans, Pôle de chimierue de Chartres, BP 6759, 45067 Orléans Cedex 2, France

**Keywords:** ADMET analysis, anticancer activity, 2,5-diaryloxazolo[4,5-*d*]pyrimidin-7-ylamines

## Abstract

Nine novel functionalized 2,5-diaryloxazolo[4,5-*d*]pyrimidin-7-ylamines were designed, synthesized and characterized using ^1^H, ^13^C NMR, IR spectroscopy, elemental analysis, and liquid chromatography–mass spectrometry*.* Their anticancer activity was assessed against human cancer cell as well as non-cancer cell lines. Three compounds, **1**, **3**, and **9**, were the most cytotoxic to HeLa cells (IC_50_ = 6.13 ± 1.95, 13.99 ± 1.80 and 49.92 ± 3.98 μM, respectively). However, only compounds **1**, **7**, and **9** were selective against the tested lines. Compound **9** can be classified as the most attractive and promising candidate for further development against cervical adenocarcinoma.

## Introduction

Purine-mimicking scaffolds are a proven strategy in the design of anticancer drugs. Many cancer-related proteins (e.g., kinases, ATPases, DNA/RNA polymerases) have binding pockets designed for purine nucleotides (ATP, GTP). Oxazolopyrimidines can compete with purines or their analogues, inhibiting enzymatic activity. They combine purine-like recognition features with the synthetic flexibility of heterocycles, offering a platform for selective targeting of tumor-related enzymes and receptors. Tumor cells overexpress kinases, DNA/RNA polymerases, and metabolic enzymes that bind purine nucleotides. Cancer cell lines such as HepG2 (liver), HeLa (cervix), A549 (lung), and glioblastoma models rely on enhanced nucleotide metabolism to sustain rapid DNA/RNA synthesis. Oxazolopyrimidines can inhibit these overactive enzymes in cancer cells, while normal cells (with lower demand) are less affected. This explains why oxazolopyrimidine derivatives may demonstrate selective cytotoxicity against cancer cell lines like HeLa, HepG2, A549, and central nervous system (CNS) tumor models [1–3].

The introduction of heterocyclic molecules with different amino groups as hydrogen-bond donors (and sometimes acceptors via lone electron pairs) can increase water solubility and facilitate salt formation. Introducing amino groups into heterocycles can have a significant impact on the anticancer activity of a compound [4–5].

Many studies have investigated the anticancer potential of heterocycle derivatives containing cytisine, glucamine, and the Strecker amine (aminoethylamine) moieties. Cytisine has been shown to inhibit the proliferation of A549, HepG2, EC109, K562, HL-60, and U937 cells. Following a 48 h treatment, IC_50_ values were approximately 2 mM in HL-60 and U937 cells, whereas higher concentrations were required to inhibit K562 and EC109 cells (IC_50_ > 12 mM). In A549 and HepG2 cells, cytisine exhibited activity, with IC_50_ values of 4.40 ± 1.70 mM and 5.92 ± 2.77 mM, respectively [6]. Heterocycle hybrids with sugar moieties have shown selective cytotoxicity against liver cancer (HepG2 – IC_50_ values of 1.32–9.43 µM) and breast cancer cells (MCF-7 – IC_50_ 1.18–11.81 µM) by increasing intracellular accumulation [7].

A significant number of studies has been published on the anticancer activity of oxazolo[5,4-*d*]pyrimidines, showing their significant activity as agonists and antagonists of signaling pathways involved in the regulation of the cell life cycle [8]. On the contrary, oxazolo[4,5-*d*]pyrimidines represent a poorly studied class of compounds because of limited access to this scaffold [9]. An in silico study showed that both isomeric forms of oxazolopyrimidines can form stable complexes due to hydrogen bonds between a lone electron pair at the nitrogen atoms and protonated amino acids of proteins. However, oxazolo[5,4-*d*]pyrimidine forms more stable complexes, which is in good agreement with in vitro studies [10].

**Scheme 1:**
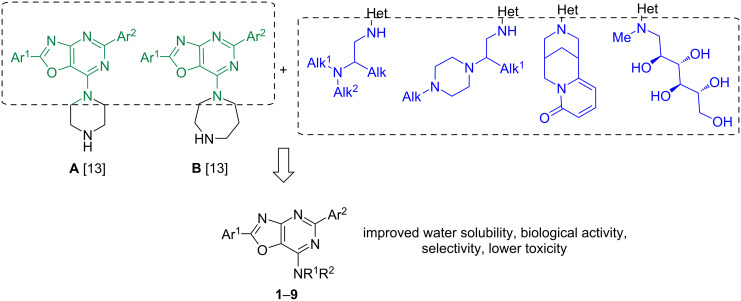
Rational design of [1,3]oxazolo[4,5-*d*]pyrimidin-7-amine derivatives **1**–**9**.

We have already shown the anticancer potential of 7-*N*-derivatives of 2,5-diaryl[1,3]oxazolo[4,5-*d*]pyrimidines and a series of 7-*N*-(1,4-diazepane)- and 7-*N*-piperazine-substituted 2,5-diaryl[1,3]oxazolo[4,5-*d*]pyrimidines were highly effective anticancer agents, as proven in a five-dose assay in NCI60 cancer cell line screen [11–12]. The most active derivatives of both series displayed high total antiproliferative and cytotoxic activity against the tested NCI60 cancer cell lines. Also, several other 7-(1,4-diazepan)- and 7-piperazine-substituted [1,3]oxazolo[4,5-*d*]pyrimidines (structures **A** and **B** in Scheme 1) showed cytotoxic activity in the micromolar concentration range against most breast cancer cell lines represented in the corresponding NCI60 subpanel derived from leukemia, melanoma, non-small-cell lung carcinoma, and cancers of the brain, ovary, breast, colon, kidney, and prostate [13]. These results provided evidence that the compound could be helpful in developing new anticancer drugs. The combination of the [1,3]oxazolo[4,5-*d*]pyrimidin-7-amine fragment (structures **A** and **B**) with cytisine, glucamine, and aminoethylamine represents a promising strategy for the rational design of multifunctional hybrids with improved biological activity, water solubility, lower toxicity and target engagement potential.

Therefore, further functionalization of 2,4-diaryl[1,3]oxazolo[4,5-*d*]pyrimidines at position 7 of the structure-forming core was carried out in this work.

## Results and Discussion

### Chemistry

**Scheme 2:**
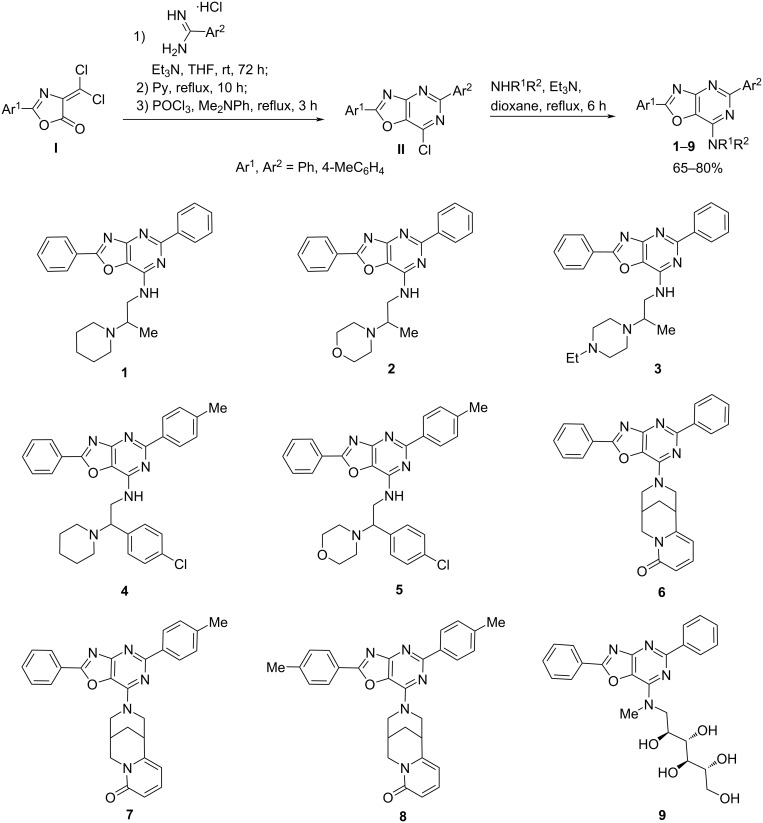
Synthesis of new 2,5-diaryloxazolo[4,5-*d*]pyrimidin-7-ylamines **1**–**9**.

The synthesis of 1,3-oxazolo[4,5-*d*]pyrimidine derivatives **1**–**9** was accomplished according to the previously described methods [14–16] as depicted in Scheme 2.

The 7-Amino-substituted 1,3-oxazolo[4,5-*d*]pyrimidines **1**–**9** were obtained with high yields (65–80%) by the reaction of 2,5-diaryl-7-chloro-1,3-oxazolo[4,5-*d*]pyrimidines **II** with the corresponding amine (aminoethylamines [17], cytisine and *N*-methyl-ᴅ-glucamine). Compounds **II** were obtained by the sequence of reactions starting from available 2-aryl-4-dichloromethylene-1,3-oxazol-5(4*H*)-ones **I** [18]. Thus, treatment of compounds **I** with arylamidine hydrochlorides in the presence of triethylamine, followed by heating with pyridine, afforded the cyclocondensation products – [1,3]oxazolo[4,5-*d*]pyrimidines. Subsequent reaction with phosphoryl chloride in the presence of *N,N*-dimethylaniline converted these intermediates into 2,5-diaryl-7-chloro[1,3]oxazolo[4,5-*d*]pyrimidines **II**.

The structures of compounds **1**–**9** were proven and confirmed by ^1^H and ^13^C NMR, IR spectroscopy, LC–MS and elemental analysis (Supporting Information File 1, Figures S1–S36). The IR spectra of compounds **1**–**5** showed the presence of NH absorption bands in the range 3381–2936 cm^−1^, OH at 3396 cm^−1^ (compound **9**), and C=O at 1601–1610 cm^−1^ (compounds **6-8**).

### Cytotoxic potency of the compounds **1**–**9**

Cytotoxic properties of the studied compounds were assessed on the non-cancer cell line NCTC clone 929 (Mus musculus normal subcutaneous connective tissue cells), as well as four human cancer cell lines: HeLa (Human cervix adenocarcinoma cells), A549 (Human lung carcinoma cells), HepG2 (Human hepatocellular carcinoma cells), and T98G (Human glioblastoma multiforme cells). Cytotoxicity was determined by measurement of 50% inhibition of cell growth by the MTT (3-(4,5-dimethylthiazol-2-yl)-2,5-diphenyltetrazolium bromide) assay. The selectivity index (SI) was calculated for the investigated compounds. The cytotoxicity results (CC_50_, µM) for the investigated compounds and the calculated selectivity index values are presented in Table 1.

**Table 1:** Results of in vitro anticancer screening of the compounds **1 9**.

Compd.	Non-cancer cells	Cancer cells							
		
	NCTC clone 929	HeLa		A549		HepG2		T98G	
					
	CC_50_^a^	IC_50_	SI^b^	IC_50_	SI	IC_50_	SI	IC_50_	SI

**1**	51.33 ± 7.91	6.13 ± 1.95	8.37	32.76 ± 2.63	1.57	85.43 ± 4.37	0.60	245.26 ± 19.17	0.21
**2**	>1000	>1000	nd^c^	>1000	nd	>1000	nd	>1000	nd
**3**	21.60 ± 4.61	13.99 ± 1.80	1.54	56.23 ± 11.10	0.38	39.02 ± 3.74	0.55	161.93 ± 7.36	0.13
**4**	81.19 ± 9.52	98.05 ± 4.11	0.82	251.86 ± 39.85	0.32	223.16 ± 31.97	0.36	>1000	<0.10
**5**	72.63 ± 15.73	58.26 ± 7.57	1.25	465.88 ± 26.13	0.16	>1000	<0.10	>1000	<0.10
**6**	>1000	>1000	nd	>1000	nd	>1000	nd	>1000	nd
**7**	>1000	62.59 ± 8.73	>15.98	922.3 ± 12.94	>1.08	>1000	nd	>1000	nd
**8**	>1000	>1000	nd	>1000	nd	>1000	nd	>1000	nd
**9**	706.50 ± 48.51	49.92 ± 3.98	14.15	920.23 ± 79.92	0.77	>1000	<0.71	>1000	<0.71
**PTX**	54.67 ± 5.77	0.02 ± 0.00	2733.50	36.00 ± 6.54	1.52	56.67 ± 8.08	0.96	0.03 ± 0.00	1822.33

^a^CC_50_ and IC_50_ are expressed in µM; ^b^SI, the selectivity index is calculated as SI = CC_50_/IC_50_; ^c^nd, not defined; data are presented as mean ± S.D. of three replicates. Paclitaxel (PTX) was used as a positive control.

The compounds **2**, **6**, and **8** were found to be non-toxic towards all the cancer and non-cancer cell lines investigated (CC_50_ > 1000 µM). In general, cancer cells form the following rows in the order of decreasing sensitivity to the active compounds:

HeLa: **1** > **3** > **9** > **5** > **7** > **4**

A549: **1** > **3** > **4** > **5** > **7** = **9**

HepG2: **3** > **1** > **4**

T98G: **3** > **1**

It was observed that compounds **1** and **3** showed the highest cytotoxicity against all cancer cells. Compound **1** exhibited the highest selectivity towards HeLa cells (SI = 8.37), suggesting potential selective toxicity. Its activity against other cancer lines was less pronounced (SI < 2). Moreover, compounds **7** and **9** showed high selectivity (SI > 10), and **1** demonstrated only moderate one (SI < 10) to the HeLa cell line. Compound **9** also showed noteworthy selectivity for HeLa cells (SI = 14.15), with a high CC_50_ value for non-cancer NCTC clone 929 cells (706.50 µM), suggesting low general toxicity. Compound **3** displayed low selectivity towards HeLa cells (SI = 1.54). All compounds showed very low SI values for other cancer lines (SI ≤ 0.6), indicating limited therapeutic potential.

Analyzing the structure–activity relationship with respect to the HeLa cancer cell line, it can be seen that among the diphenyloxazolopyridine derivatives, the substitution of the piperidine functional motif at position 7 in compound **1** by 4-ethylpiperazine leads to a decrease in the cytotoxicity by two times (compound **3**). Whereas its substitution by morpholine lead to inactive compound **2**. The same result was obtained by substituting 2-piperidin-1-ylpropylamine in **1** with hexahydro-1,5-methanopyrido[1,2-*a*][1,5]diazocin-8-one (**6**). However, methylation of the phenyl group at the 5-position of compound **6** resulted in restoration of activity (**7**). In contrast, simultaneous introduction of a methyl group in both phenyl residues of the oxazolopyridine ring of compound **6** did not give a positive result (**8**). The combined replacement of the 5-phenyl substituent in compound **1** by a 4-methylphenyl group and addition of a 4-chlorophenyl group instead of the methyl group as a functional motif at position 7 (**4**) sharply reduced the cytotoxicity of compound **1**. The substitution of piperidine by morpholine in this motif in compound **4** resulted in a 2-fold increase in the activity of the obtained derivative **5**. However, compound **5** was one order less potent than compound **1** in terms of its cytotoxic potency.

It is important to note that among the compounds that have demonstrated high activity against cancer cells, a significant indicator of their potential as drug candidates is cytotoxic selectivity relative to non-cancer cells. Therefore, regardless of the concentration in effect, the compound with greater selectivity should be favored. According to this criterion, compounds **1**, **7**, and **9** show the most significant appeal for further testing as potential agents against cervical cancer. Unfortunately, none of these compounds was anywhere near the activity of paclitaxel against HeLa cells (SI = 2733.50).

### ADMET analysis

ADMET (absorption, distribution, metabolism, excretion, toxicity) analysis performed among active in vitro anticancer compounds allows to identify the most promising agents to be sent to further stages of drug candidate development, taking into account the expected pharmacokinetic and toxicological, as well as undesirable properties of the molecules. At the same time, drug similarity filters developed by pharmaceutical companies based on the physicochemical properties of approved drugs with known mechanisms of action have utilitarian value, as they do not account for the probability of including targets not inherent to existing drugs in their molecular mechanisms of anticancer action. The physicochemical properties of such compounds may not fit the parameters developed from databases of approved drugs, thereby hindering the search for drug candidates with unique anticancer properties [19]. Practically, this means that the failure of any new compounds with in vitro anticancer activity and acceptable pharmacokinetic and toxicological properties to pass these filters requires a tailored approach. It should be noted, however, that different ADMET platforms may yield conflicting results, especially regarding the metabolism and cytotoxic molecular targets of the analyzed compounds, due to different approaches used to virtually evaluate these parameters. Therefore, indeed, the equivalence of results for a particular parameter obtained from different platforms increases the reliability of the prediction [20]. In addition, during the development of chemotherapeutics, oral bioavailability is desirable but not critical, given the preference for the parenteral route of administration.

Due to the probabilistic nature of the evaluation, parameters for which the probability of a compound's relevant property is predicted to occur in the range 0.3 < p < 0.7 does not allow a confident conclusion to be drawn and require further evaluation [21]. The same applies to the conflicting results obtained by different platforms.

### Pharmacokinetic properties

ADMET analysis enables the prediction of a compound's pharmacokinetic profile, which is crucial for assessing its pharmacodynamic activity. The predicted pharmacokinetic properties of compounds **1**, **7** and **9** are given in Table 2.

**Table 2:** Complex pharmacokinetic profile of compounds **1**, **7**, and **9** predicted by ADMETlab3.0.

Parameter	ADMETlab3.0		
	
	**1**	**7**	**9**

HIA^a^	0.000	0.000	0.000
BBB permeant^b^	0.999	0.998	0.754
Pgp substrate^c^	0.004	0.000	0.001
Pgp inhibitor	0.950	0.949	0.013
CYP1A2 substrate^d^	0.623	0.053	0.000
CYP2C19 substrate	0.119	0.636	0.000
CYP2C9 substrate	0.000	0.142	0.000
CYP2D6 substrate	0.401	0.088	0.000
CYP3A4 substrate	0.242	0.208	0.000
CYP2B6 substrate	0.021	0.019	0.020

^a^HIA, human intestinal absorption; ^b^BBB, blood–brain barrier penetration; ^c^Pgp, glycoprotein P; ^d^CYP, cytochrome P450.

For human intestinal absorption, the virtual platforms gave mutually exclusive results. However, as mentioned above, this parameter is not critical for anticancer compounds.

It should be noted that the training samples of medicines used by all ADMET platforms are generic, i.e., medicines used to treat a wide range of diseases whose medical requirements may be mutually exclusive. Consequently, the underlying training samples used to calibrate the data may also differ depending on the application route of the medicine. Therefore, due to the conflicting results of BBB permeability predicted for compound **9** by common ADMET platforms, a specific drug identity filter for CNS-penetrating compounds was utilized (Table 3) [22].

**Table 3:** The parameters of physicochemical properties of compounds **1**, **7**, and **9** determine the threshold values of BBB permeability according to the CNS rule descriptor.

Descriptor	Compound			CNS rule
		
	**1**	**7**	**9**	

MW^a^	413.2	475.2	466	135–582
logP^b^	5.6	4.7	5.4	−0.2 to 6.1
*n*HA^c^	6.0	7.0	10.0	≤5
*n*HD^d^	1.0	0.0	5.0	≤3
tPSA^e^	67	77	156	3–118

^a^MW, molecular weight; ^b^LogP, octanol/water distribution coefficient; ^c^*n*HA, number of H–bond acceptors; ^d^*n*HD, number of H–bond donors; ^e^tPSA, topological polar surface area.

It follows from the presented data that, according to the value of physicochemical parameters used to predict the BBB permeability of anticancer CNS targeted compounds, compound **9** (two deviations: descriptors *n*HA and *n*HD), unlike **1** and **7** (one deviation: *n*HA, that is acceptable for decision-making), does not pass the CNS filter. In other words, it belongs to the group of compounds that do not pass the blood–brain barrier. This finding is also entirely consistent with the predicted ADMETlab3.0 neurotoxicity of these compounds (Table 3). With regard to the substrate specificity of Pgp, it is highly probable that these compounds are not substrates of this protein, although compounds **1** and **7**, unlike **9**, appear to be capable of inhibiting it (Table 3). A low probability of oxidative metabolism of these compounds by the studied cytochromes is also predicted.

### Human toxicity of compounds **1**, **7** and **9**

The results of predicting the human toxicity of tested compounds **1**, **7**, and **9** are presented in Table 4 and Table 5.

**Table 4:** Predicted parameters of human toxicity of compounds **1**, **7**, and **9**.

Parameter	ADMETlab3.0		
	
	**1**	**7**	**9**

cardiotoxicity (hERG blocker)	0.802	0.469	0.172
DILI (drug-induced liver injury)	0.832	0.967	0.651
carcinogenicity	0.480	0.644	0.072
respiratory toxicity	0.755	0.587	0.003
nephrotoxicity	0.441	0.566	0.310
neurotoxicity	0.888	0.953	0.280
hematotoxicity	0.202	0.661	0.014
genotoxicity	0.517	0.989	0.082
RPMI-8226 immunitoxicity	0.043	0.085	0.021

**Table 5:** Predicted probability of interaction of compound **1**, **7**, and **9** with nuclear receptor (NR) signaling, and stress response (SR) pathways.

Target	ADMETlab3.0^a^			Deep-PK	
		
	**1**	**7**	**9**	**1**	**9**

NR-AhR^b^	++	-	---	0.069	0.079
NR-AR^c^	--	---	---	0.170	0.203
NR-AR-LBD^d^	++	---	---	0.041	0.013
NR-Aromatase	+	--	---	0.010	0.020
NR-ER^e^	++	--	---	0.235	0.039
NR-ER-LBD^f^	---	---	---	0.000	0.000
NR-PPAR-gamma^g^	--	---	---	0.002	0.002
SR-ARE^h^	++	+	---	0.887	**0.953** ^m^
SR-ATAD5^i^	+	--	---	0.005	0.000
SR-HSE^j^	-	--	---	0.000	0.000
SR-MMP^k^	+++	-	---	0.972	0.158
SR-p53^l^	++	---	---	0.000	0.000

^a^Here and below, the prediction probabilities performed by ADMETlab3 are presented in the form of six symbols for the end points of the classification: 0–0.1(---), 0.1–0.3 (--), 0.3–0.5 (-), 0.5–0.7 (+), 0.7–0.9 (++), and 0.9–1.0 (+++); ^b^AhR, aryl hydrocarbon receptor; ^c^AR, androgen receptor; ^d^AR-LBD, androgen receptor ligand-binding domain; ^e^ER, estrogen receptor; ^f^ER-LBD, estrogen receptor ligand-binding domain; ^g^PPAR-gamma, peroxisome proliferator-activated receptor gamma; ^h^ARE, antioxidant response element; ^i^ATAD5, ATPase family AAA domain-containing protein 5; ^j^HSE, heat shock factor response element; ^k^MMP, mitochondrial membrane potential; ^l^p53, a tumor suppressor protein.

The possible molecular targets predicted by the virtual platforms through which the toxic effects of the tested compounds on the human body could be realized and are presented in Table 5.

According to Table 5, compound **1** is expected to interact with three nucleic acid receptors and three stress signaling pathways with high probability. However, the cytotoxicity of compounds **7** and **9** is likely not due to interactions with the tested targets.

It should also be noted that both compounds pass the pan-assay interference compounds (PAINS) filter. That is, they do not belong to chemical compounds that can nonspecifically interfere with biological assays, leading to false-positive results. Consequently, their activity is not due to interference with the assay itself, but to binding to possible targets.

## Conclusion

The results of anticancer screening and virtual analysis suggest that among the synthesized derivatives, compounds **1**, **7**, and **9** showed activity and high relevant selectivity against the HeLa cell line. While compound **1** exhibited a higher degree of cytotoxic potency in comparison to compounds **7** and **9**, the latter compounds demonstrated a substantially higher degree of selectivity. This finding suggests that compounds **7** and **9** would be associated with a reduced risk of severe adverse side effects during clinical usage. This is supported by virtual assay results indicating low human toxicity, low probability of interaction with receptors in the Tox21 pathways, very weak substrate specificity for Pgp, very low likelihood of oxidative metabolism, and inability of compound **9** to cross the blood–brain barrier. Hence, according to the above analysis, compounds **7** and **9** may prove to be more promising agents for further development as a drug candidate against human cervix adenocarcinoma.

## Experimental

### Chemistry

All reagents and solvents used in synthetic procedures were purchased from Sigma-Aldrich and used as received. The reactions were followed by TLC (Silica gel, aluminum sheets 60 F_254_, Merck). Melting points were recorded on a Fisher-Johns apparatus. ^1^H and ^13^C NMR spectra were recorded on a Varian Mercury spectrometer (400 and 101 MHz, respectively) or Bruker Avance DRX 500 spectrometer (500 MHz and 126 MHz, respectively) in CDCl_3_ or CF_3_C(O)OD, taking its residual solvent signal as a standard. Multiplicities were described using the following abbreviations: s = singlet, br s = broad singlet, d = doublet, dd = doublet of doublets, t = triplet, and m = multiplet. LC–MS analysis was performed on an Agilent 1200 Series system equipped with a diode array and a G6130A mass spectrometer (atmospheric pressure electrospray ionization). Combustion elemental analysis was performed in the V.P. Kukhar Institute of Bioorganic Chemistry and Petrochemistry analytical laboratory and the results were found to be in good agreement (±0.4%) with the calculated values. The carbon and hydrogen contents were determined using the Pregl gravimetric method, nitrogen by using Dumas' gasometric micromethod, and chlorine by the mercurimetric method.

*N*-Methyl-ᴅ-glucamine and (−)-cytisine ((1*R*,5*S*)-1,2,3,4,5,6-hexahydro-1,5-methano-8*H*-pyrido[1,2-*a*][1,5]diazocin-8-one) are commercially available reagents from Sigma-Aldrich. Aminoethylamines were synthesized according to the literature method [16] and used as racemic mixtures.

### General procedure for the synthesis of oxazolo[4,5-*d*]pyrimidines **1**–**9**

A solution of 0.01 mol of the corresponding 7-chlorooxazolo[4,5-*d*]pyrimidines **IІ** in 30 mL of anhydrous dioxane was added dropwise to a solution of 0.03 mol of the appropriate amine and 0.01 mol of triethylamine in 20 mL of anhydrous dioxane over 0.5 h. The reaction mixture was heated for 5 h at 100–110 °C and left for 12 h at 20–25 °C. The solvent was removed in vacuo, the residue was treated with water, and the solid precipitate was filtered off and dried. The formed compounds **1**–**9** were purified by crystallization from acetonitrile [17].

### Spectral data

Analytical and spectral data, IR, ^1^H, ^13^C NMR and LC–MS spectra of compounds **1–9** are given in Supporting Information File 1.

### Cytotoxicity assay

Stock solutions of each compound were prepared in DMSO at 100 mM and diluted into medium supplemented with 10% FBS. The final DMSO content in the solutions did not exceed 0.1%.

Cancer cell lines: HeLa (ATCC CCL-2, Human cervix adenocarcinoma cells), A549 (ATCC CCL-185, Human lung carcinoma cells), HepG2 (ATCC HB-8065, Human hepatocellular carcinoma cells), and T98G (ATCC CRL-1690, Human glioblastoma multiforme cells). Non-cancer cell line: NCTC clone 929 (CCL-1, Mus musculus normal subcutaneous connective tissue cells).

Chemicals: Eagles minimum essential medium (MEM, Sigma-Aldrich), fetal bovine serum (FBS, Sigma-Aldrich), antibiotics (Sigma-Aldrich), trypsin (Life Technologies), 3-(4,5-dimethylthiazol-2-yl)-2,5-diphenyltetrazolium bromide (MTT, Sigma-Aldrich), dimethylformamide (DMF, Sigma-Aldrich), sodium dodecylsulfate (SDS, Sigma-Aldrich).

HeLa, HepG2, A549, T98G, and NCTC clone 929 cells were propagated in MEM supplemented with 10% heat-inactivated FBS and 1% penicillin/streptomycin mixture (10 000 units/mL penicillin G with 10 mg/mL streptomycin). Upon reaching 80–90% confluency, cells were harvested with 0.25% trypsin in 1 mM EDTA and seeded into 96-well microplates at 2 × 10^4^ cells/well. After overnight incubation of cells at 37 °C in a humidified atmosphere containing 5% CO_2_, the culture medium was removed and replaced with a 100 μL of freshly prepared solution of compounds diluted with the growth medium supplemented with 10% FBS and 1% penicillin/streptomycin mixture, for obtaining concentrations ranging from 0.1 to 1000 µM. All experiments were carried out in triplicate, and the data are presented as mean ± standard deviation. Paclitaxel (PTX) was used as positive control. Compounds-treated and untreated cells were incubated at 37 °C for 48 h in humidified atmosphere containing 5% CO_2_. After that the cytotoxicity of investigated compounds was analyzed microscopically and cell viability was determined with the MTT assay. After incubation with drugs the cells were treated with 25 μL of 3-(4,5-dimethylthiazol-2-yl)-2,5-diphenyltetrazolium bromide (MTT) dye solution (5 mg/mL) for 2 h and lysed with 100 μL of solvent solution containing: 45 mL of dimethylformamide (DMF), 13.5 g of sodium dodecylsulfate (SDS), and 55 mL distilled water [23]. After overnight incubation at 37 °C optical density at 550 nm and with a reference wavelength of 670 nm was measured in an ELISA reader (VarioskanLux, Thermo Scientific) and CC_50_ (50% cytotoxic concentration) was calculated.

### ADMET analysis

Available online web sites of an integrated online platform for Windows: ADMETlab 3.0 [24] and Deep-PK [25] were applied to explore ADMET properties of the studied molecules. The pharmacokinetic, pharmacodynamic, and toxic targets for the test compounds were predicted. The conversion of molecular structures into SMILES strings, which is required for the operation of ADMET platforms, was done using the Marvin JS widget [26].

## Supporting Information

File 1 Characterization data and copies of spectra.

## Data Availability

All data that supports the findings of this study is available in the published article and/or the supporting information of this article.
