# Supplementary material for: Design, synthesis and biological evaluation of 2,5-diaryloxazolo[4,5-d]pyrimidin-7-ylamines as selective cytotoxic agents against HeLa cells
Source: Beilstein J Org Chem. 2026 Mar 3;22:390–8. doi: 10.3762/bjoc.22.27 (PMC12973442; doi:10.3762/bjoc.22.27)
Supplement: File 1 — Characterization data and copies of spectra. [file Beilstein_J_Org_Chem-22-390-s001.pdf]

BEILSTEIN JOURNAL OF ORGANIC CHEMISTRY

Supporting Information

for

Design, synthesis and biological evaluation of 2,5-diaryloxazolo[4,5-*d*]pyrimidin-7-ylamines as selective cytotoxic agents against HeLa cells

Maryna V. Kachaeva, Agnieszka B. Olejniczak, Marta Denel-Bobrowska, Victor V. Zhirnov, Yevheniia S. Velihina, Stepan G. Pilyo and Volodymyr S. Brovarets

Beilstein J. Org. Chem. **2026,** *22,* 390–398. doi:10.3762/bjoc.22.27

Characterization data and copies of spectra

License and Terms: This is a supporting information file under the terms of the Creative Commons Attribution License (https://creativecommons.org/licenses/by/4.0). Please note that the reuse, redistribution and reproduction in particular requires that the author(s) and source are credited and that individual graphics may be subject to special legal provisions.
The license is subject to the *Beilstein Journal of Organic Chemistry* terms and conditions: (https://www.beilstein-journals.org/bjoc/terms)

Table of contents

Analytical and spectral data of compounds **1**–**9**	S2–S4
IR, ^{1}H, ^{13}C NMR and LC–MS spectra of products	S5
Figures S1–S36. IR, ^{1}H, ^{13}C NMR and LC–MS spectra of compounds **1**–**9**.	S5–S51

Analytical and spectral data of compounds 1–9

2,5-Diphenyl-*N*-(2-piperidin-1-ylpropyl)-[1,3]oxazolo[4,5-*d*]pyrimidin-7-amine (1)

Yield 80%. White solid, mp 161–163 °C. IR (KBr, cm^{-1}) ν_{max}: 3336-2936 (NH, Ar-CH), 1647, 1553, 1483, 1381, 1367, 771, 702. ^{1}H NMR (400 MHz, $CDCl_3$) δ 8.54 (d, 2H, J_{HH} = 8.0 Hz, ArH), 8.31 (d, 2H, J_{HH} = 8.0 Hz, ArH), 7.60-7.56 (m, 3H, ArH), 7.47-7.45 (m, 3H, ArH), 6.35 (br s, 1H, NH), 4.08-3.88 (m, 1H, CH), 3.44 (br s, 1H, CH), 3.00 (br s, 1H, CH), 2.69 (br s, 2H, CH_2), 2.46 (br s, 2H, CH_2), 1.80-1.66 (m, 4H, $2CH_2$), 1.53 (br s, 2H, CH_2), 1.13 (d, 3H, J_{HH} = 8.0 Hz, CH_3). ^{13}C NMR (126 MHz, $CDCl_3$) δ 165.0, 161.3, 148.2, 138.3, 132.3, 130.7, 129.9, 129.0, 128.4, 128.2, 128.1, 128.0, 126.6, 58.5, 49.1, 26.6, 26.5, 24.8, 11.1. LC-MS: *m/z* (%) 414.2 (96.7) $[M+1]^+$. Anal. Calcd for $C_{25}H_{27}N_5O$: C, 72.61; H, 6.58; N, 16.94. Found: C, 72.53; H, 6.52; N, 16.76.

***N*-(2-Morpholin-4-ylpropyl)-2,5-diphenyl-[1,3]oxazolo[4,5-*d*]pyrimidin-7-amine (2)**

Yield 80%. White solid, mp 186-188 °C. IR (KBr, cm^{-1}) ν_{max}: 3307-2971 (NH, Ar-CH), 2807, 1645, 1581, 1512, 1480, 1449, 1368, 1311, 1265, 1217, 1144, 1114, 1024, 966, 923, 872, 773, 703. ^{1}H NMR (400 MHz, $CDCl_3$) δ 8.50-8.49 (m, 2H, ArH), 8.23-8.22 (m, 2H, ArH), 7.55-7.43 (m, 6H, ArH), 6.05 (br s, 1H, NH), 4.02-3.78 (m, 5H, ArH), 3.44 (t, 1H, J_{HH} = 8 Hz, CH), 2.95 (br s, 1H, CH), 2.70 (br s, 2H, CH_2), 2.54 (br s, 2H, CH_2), 1.13 (d, 3H, J_{HH} = 8.0 Hz, CH_3). ^{13}C NMR (126 MHz, $CDCl_3$) δ 165.0, 161.1, 148.0, 138.2, 132.5, 132.4, 130.0, 129.9, 129.0, 128.3, 128.2, 128.0, 126.4, 67.4, 58.3, 48.4, 42.8, 11.5. LC-MS: *m/z* (%) 416.2 (93.2) $[M+1]^+$. Anal. Calcd for $C_{24}H_{25}N_5O_2$: C, 69.38; H, 6.06; N, 16.86. Found: C, 69.30; H, 6.00; N, 16.99.

***N*-[2-(4-Ethylpiperazin-1-yl)propyl]-2,5-diphenyl-[1,3]oxazolo[4,5-*d*]pyrimidin-7-amine (3)**

Yield 76%. White solid, mp 145–147 °C. IR (KBr, cm^{-1}) ν_{max}: 3381-2966 (NH, Ar-CH), 2811, 1642, 1502, 1369, 702. ^{1}H NMR (400 MHz, $CDCl_3$) δ 8.53 (d, 2H, J_{HH} = 4 Hz, ArH), 8.29 (d, 2H, J_{HH} = 8 Hz, ArH), 7.59-7.53 (m, 3H, ArH), 7.46-7.45 (m, 3H, ArH), 6.12 (br s, 1H, NH), 3.93-3.87 (m, 1H, CH), 3.51-3.45 (m, 1H, CH), 3.04-3.01 (m, 1H, CH), 2.79 (br s, 2H, CH_2), 2.61 (br s, 5H, $2CH_2$, CH), 2.52-2.47 (m, 3H, CH_2, CH), 1.16-1.12 (m, 6H, $2CH_3$). ^{13}C NMR (126 MHz, $CDCl_3$) δ 165.1, 161.3, 148.2, 138.2, 132.4, 129.9, 129.1, 129.0, 128.4, 128.2, 128.1, 128.0, 126.6, 57.9, 53.3, 52.4, 11.9, 11.6. LC-MS: *m/z* (%) 443.2 (100) $[M+1]^+$. Anal. Calcd for $C_{28}H_{23}N_5O_2$: C, 72.87; H, 5.02; N, 15.17. Found: C, 72.76; H, 5.09; N, 15.41.

***N*-[2-(4-Chlorophenyl)-2-piperidin-1-ylethyl]-5-(4-methylphenyl)-2-phenyl-[1,3]oxazolo[4,5-*d*]pyrimidin-7-amine (4)**

Yield 79%. White solid, mp 198–200 °C. IR (KBr, cm^{-1}) ν_{max}: 3326-2928 (NH, Ar-CH), 1680, 1636, 1552, 1481, 1366, 1159, 1093, 777, 690. ^{1}H NMR (400 MHz, $CDCl_3$) δ 8.40 (d, 2H,

J_{HH} = 8 Hz, ArH), 8.26 (d, 2H, J_{HH} = 8 Hz, ArH), 7.61-7.52 (m, 3H, ArH), 7.37 (d, 2H, J_{HH} = 8 Hz, ArH), 7.27-7.25 (m, 4H, ArH), 5.95 (br s, 1H, NH), 4.13-4.01 (m, 2H, CH_2), 3.84-3.80 (m, 1H, CH), 2.57-2.53 (m, 2H, CH_2), 2.41-2.40 (m, 5H, CH_3, CH_2), 1.69-1.61 (m, 4H, $2CH_2$), 1.44 (dd, 2H, J_{HH} = 12, 8 Hz, CH_2). ^{13}C NMR (101 MHz, $CDCl_3$) δ 164.7, 161.3, 145.6, 140.1, 135.4, 135.3, 133.6, 132.4, 130.0, 129.0, 128.9, 128.5, 128.3, 128.0, 126.5, 125.3, 36.9, 26.5, 23.8, 21.5, 17.5. LC-MS: *m/z* (%) 525.2 (95.8) $[M+1]^+$. Anal. Calcd for $C_{31}H_{30}ClN_5O$: C, 71.05; H, 5.77; Cl, 6.76; N, 13.36. Found: C, 71.13; H, 5.80; Cl, 6.87; N, 13.56.

N-[2-(4-Chlorophenyl)-2-morpholin-4-ylethyl]-5-(4-methylphenyl)-2-phenyl-[1,3]oxazolo[4,5-*d*]pyrimidin-7-amine (5)

Yield 76%. White solid, mp 225–227 °C. IR (KBr, cm^{-1}) ν_{max}: 3301-2821 (NH, Ar-CH), 1641, 1552, 1480, 1368, 1110. ^{1}H NMR (500 MHz, $CDCl_3$) δ 8.36 (d, 2H, J_{HH} = 8 Hz, ArH), 8.21 (d, 2H, J_{HH} = 8 Hz, ArH), 7.57 (t, 1H, J_{HH} = 8 Hz, ArH), 7.52 (t, 2H, J_{HH} = 8 Hz, ArH), 7.37 (d, 2H, J_{HH} = 4 Hz, ArH), 7.28-7.23 (m, 4H, ArH), 5.60 (1H, NH), 4.24-4.19 (m, 1H, CH), 4.09-3.77 (m, 6H, CH, $2CH_2$), 2.57-2.54 (m, 4H, $2CH_2$), 2.40 (s, 3H, CH_3). ^{13}C NMR (126 MHz, $CDCl_3$) δ 165.2, 161.3, 147.8, 140.2, 135.3, 132.4, 130.0, 129.0, 128.8, 128.3, 128.1, 126.4, 68.2, 67.2, 50.5, 37.1, 21.4. LC-MS: *m/z* (%) 526.2 (95.6) $[M+1]^+$. Anal. Calcd for $C_{30}H_{28}ClN_5O_2$: C, 68.50; H, 5.37; Cl, 6.74; N, 13.31. Found: C, 68.45; H, 5.31; Cl, 6.89; N, 13.44.

11-(2,5-Diphenyl-[1,3]oxazolo[4,5-*d*]pyrimidin-7-yl)-7,11-diazatricyclo[7.3.1.02,7]trideca-2,4-dien-6-one (6)

Yield 75%. White solid, mp 225–227 °C. IR (KBr, cm^{-1}) ν_{max}: 3057, 2847, 1654, 1613, 1603 (C=O), 1545, 1475, 1373, 1067, 771, 698. ^{1}H NMR (400 MHz, $CDCl_3$) δ 8.42-8.40 (m, 2H, ArH), 8.21 (d, 2H, J_{HH} = 8 Hz, ArH), 7.61-7.52 (m, 3H, ArH), 7.44-7.43 (m, 3H, ArH), 7.14 (t, J_{HH} = 8 Hz, 1H, ArH), 6.27 (t, J_{HH} = 8 Hz, 1H, ArH), 6.16 (t, J_{HH} = 8 Hz, 1H, ArH), 5.03 (dd, J_{HH} = 28, 12 Hz, 2H, CH_2), 4.21 (d, 1H, J_{HH} = 16 Hz, CH), 3.97 (dd, J_{HH} = 16, 8 Hz, 1H, CH), 3.46 (d, 2H, J_{HH} = 12 Hz, CH_2), 3.27 (br s, 1H, CH), 2.73 (br s, 1H, CH), 2.17 (br s, 2H, CH_2). ^{13}C NMR (101 MHz, $CDCl_3$) δ 164.9, 163.3, 162.5, 160.4, 148.6, 148.2, 138.6, 137.8, 132.6, 130.0, 129.1, 128.3, 128.2, 128.2, 128.0, 127.8, 126.1, 117.6, 105.3, 49.1, 34.9, 27.8, 26.4. LC-MS: *m/z* (%) 462.2 (97.2) $[M+1]^+$. Anal. Calcd for $C_{26}H_{30}N_6O$: C, 70.50; H, 6.75; N, 18.76. Found: C, 70.56; H, 6.83; N, 18.99.

11-[5-(4-Methylphenyl)-2-phenyl-[1,3]oxazolo[4,5-*d*]pyrimidin-7-yl]-7,11-diazatricyclo[7.3.1.02,7]trideca-2,4-dien-6-one (7)

Yield 71%. White solid, mp 263–265 °C. IR (KBr, cm^{-1}) ν_{max}: 3057, 2946, 1817, 1652, 1601 (C=O), 1572, 1547, 1449, 1371, 1144, 810, 777, 712, 689. ^{1}H NMR (400 MHz, $CDCl_3$) δ 8.29 (d, 2H, J_{HH} = 8 Hz, ArH), 8.19 (d, 2H, J_{HH} = 8 Hz, ArH), 7.59-7.50 (m, 3H, ArH), 7.23 (d, 2H, J_{HH} = 8 Hz, ArH), 7.13 (t, 1H, J_{HH} = 8 Hz, ArH), 6.26 (d, 2H, J_{HH} = 8 Hz, ArH), 6.15 (d, 2H,

J_{HH} = 8 Hz, ArH), 5.00 (q, 2H, J_{HH} = 12 Hz, CH_2), 4.19 (d, 2H, J_{HH} = 16 Hz, CH), 3.96 (dd, 1H, J_{HH} = 16, 8 Hz, CH), 3.43 (d, 2H, J_{HH} = 8 Hz, CH_2), 3.26 (br s, 1H, CH), 2.72 (br s, 1H, CH), 2.40 (s, 3H, CH_3), 2.15 (br s, 1H, CH_2). ^{13}C NMR (101 MHz, $CDCl_3$) δ 164.8, 163.3, 162.4, 148.7, 148.1, 140.1, 138.6, 135.2, 132.5, 129.0, 128.9, 128.3, 127.9, 127.7, 126.1, 117.6, 105.3, 51.5, 49.1, 34.8, 27.8, 26.3, 21.5. LC-MS: *m/z* (%) 476.2 (100) $[M+1]^+$. Anal. Calcd for $C_{29}H_{25}N_5O_2$: C, 73.25; H, 5.30; N, 14.73. Found: C, 73.31; H, 5.38; N, 14.89.

11-[2,5-Bis(4-methylphenyl)-[1,3]oxazolo[4,5-*d*]pyrimidin-7-yl]-7,11-diazatricyclo[7.3.1.02,7]trideca-2,4-dien-6-one (8)

Yield 70%. White solid, mp > 250 °C. IR (KBr, cm^{-1}) ν_{max}: 2921, 2242, 1610 (C=O), 1552, 1494, 1470, 1414, 1319, 1277, 1125, 1088, 1010, 822, 736. ^{1}H NMR (500 MHz, $CDCl_3$) δ 8.40 (d, 2H, J_{HH} = 10 Hz, ArH), 8.07 (d, 2H, J_{HH} = 10 Hz, ArH), 7.41 (d, 2H, J_{HH} = 10 Hz, ArH), 7.31 (d, 2H, J_{HH} = 10 Hz, ArH), 7.13 (t, 1H, J_{HH} = 10 Hz, ArH), 6.27 (d, 1H, J_{HH} = 10 Hz, ArH), 6.16 (d, 1H, J_{HH} = 5 Hz, ArH), 5.02 (dd, J_{HH} = 35, 10 Hz, 2H, CH_2), 4.21 (d, J_{HH} = 15 Hz, 1H, CH), 3.97 (dd, J_{HH} = 15, 10 Hz, 1H, CH), 3.44 (d, J_{HH} = 10 Hz, 2H, CH_2), 3.27 (s, 1H, CH), 2.73 (s, 1H, CH), 2.47 (s, 3H, CH_3), 2.44 (s, 3H, CH_3), 2.16 (s, 2H, CH_2). ^{13}C NMR (101 MHz, $CDCl_3$) δ 165.2, 163.2, 148.7, 143.4, 138.6, 131.1, 130.0, 130.0, 129.8, 129.0, 128.6, 128.6, 128.3, 128.1, 127.9, 127.6, 123.2, 117.5, 49.1, 38.0, 34.8, 27.8, 26.3, 21.9. LC-MS: *m/z* (%) 490.2 (95.9) $[M+1]^+$. Anal. Calcd for $C_{30}H_{27}N_5O_2$: C, 73.60; H, 5.56; N, 14.30. Found: C, 73.67; H, 5.65; N, 14.57.

6-[(2,5-Diphenyl[1,3]oxazolo[4,5-*d*]pyrimidin-amino-7-yl)]-*N*-methyl-(2R,3R,4S,5S)-hexane-1,2,3,4,5-pentaol (9)

Yield 65%. White solid, mp 210–212 °C. IR (KBr, cm^{-1}) ν_{max}: 3396 (OH), 1631, 1550, 1389, 1110, 1092, 1071, 1022, 769, 703. ^{1}H NMR (500 MHz, $CF_3C(O)OD$) δ 7.58-7.50 (m, 4H, ArH), 7.13-7.04 (m, 2H, ArH), 7.00-6.96 (m, 4H, ArH), 4.35-4.00 (m, 3H, CH_2, CH), 3.85-3.80 (m, 1H, CH), 3.76-3.72 (m, 1H, CH), 3.64-3.37 (m, 3H, CH_2, CH), 3.26, 3.05 (s, 3H, CH_3). ^{13}C NMR (126 MHz, $CF_3C(O)OD$) δ 169.3, 154.2, 1495, 135.0, 134.0, 128.9, 128.7, 127.8, 127.7, 127.1, 126.8, 126.8, 121.3, 71.5, 70.8, 68.6, 66.8, 53.5, 52.5, 37.8. LC-MS: *m/z* (I_{rel}, %): 467.2 $[M+H]^+$ (100). Found, %: C 61.65; H 5.60; N 12.45. $C_{24}H_{26}N_4O_6$. Calculated, %: C 61.79; H 5.62; N 12.01.

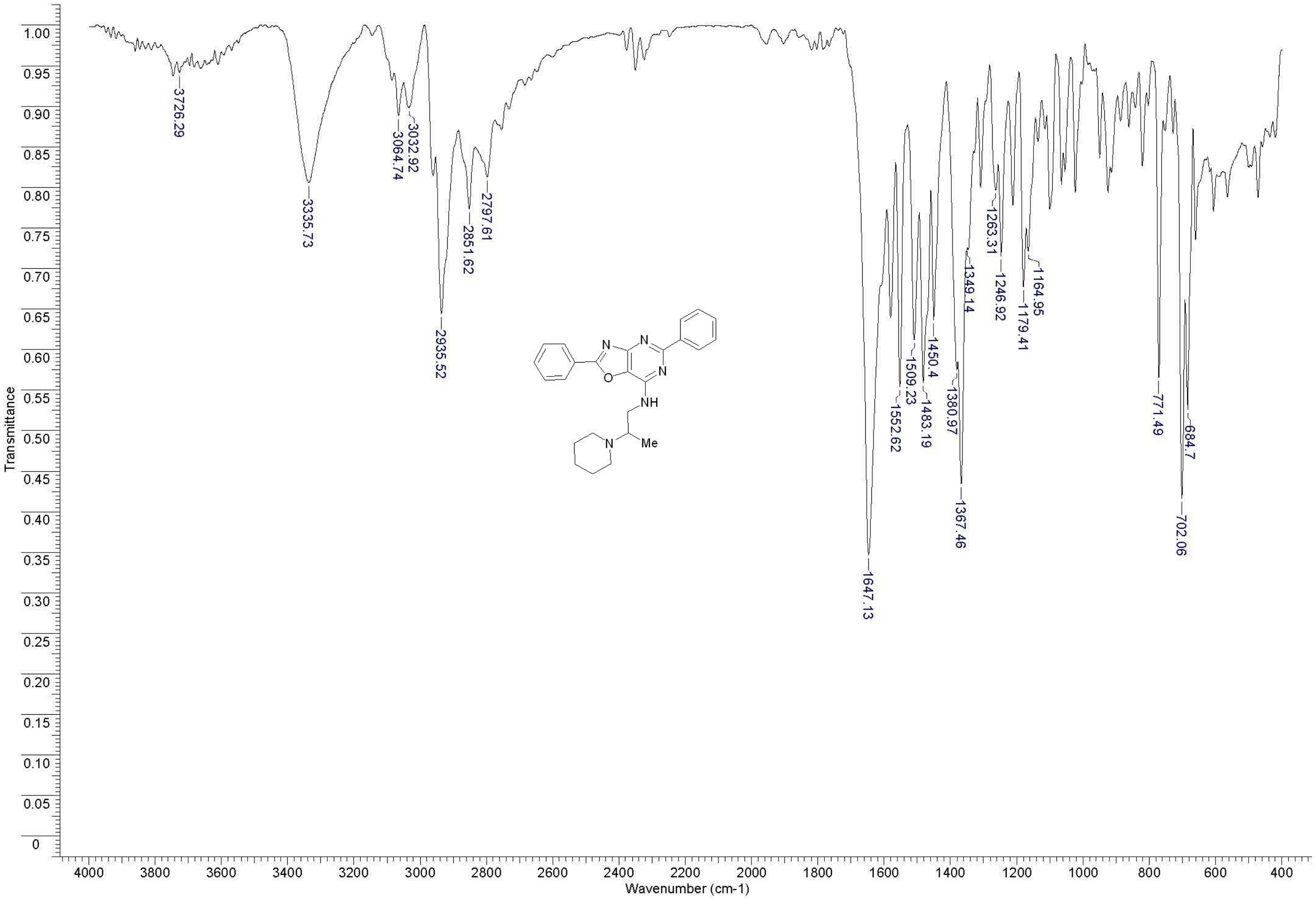

Figure S1. IR spectrum of 2,5-diphenyl-*N*-(2-piperidin-1-ylpropyl)-[1,3]oxazolo[4,5-*d*]pyrimidin-7-amine (**1**)

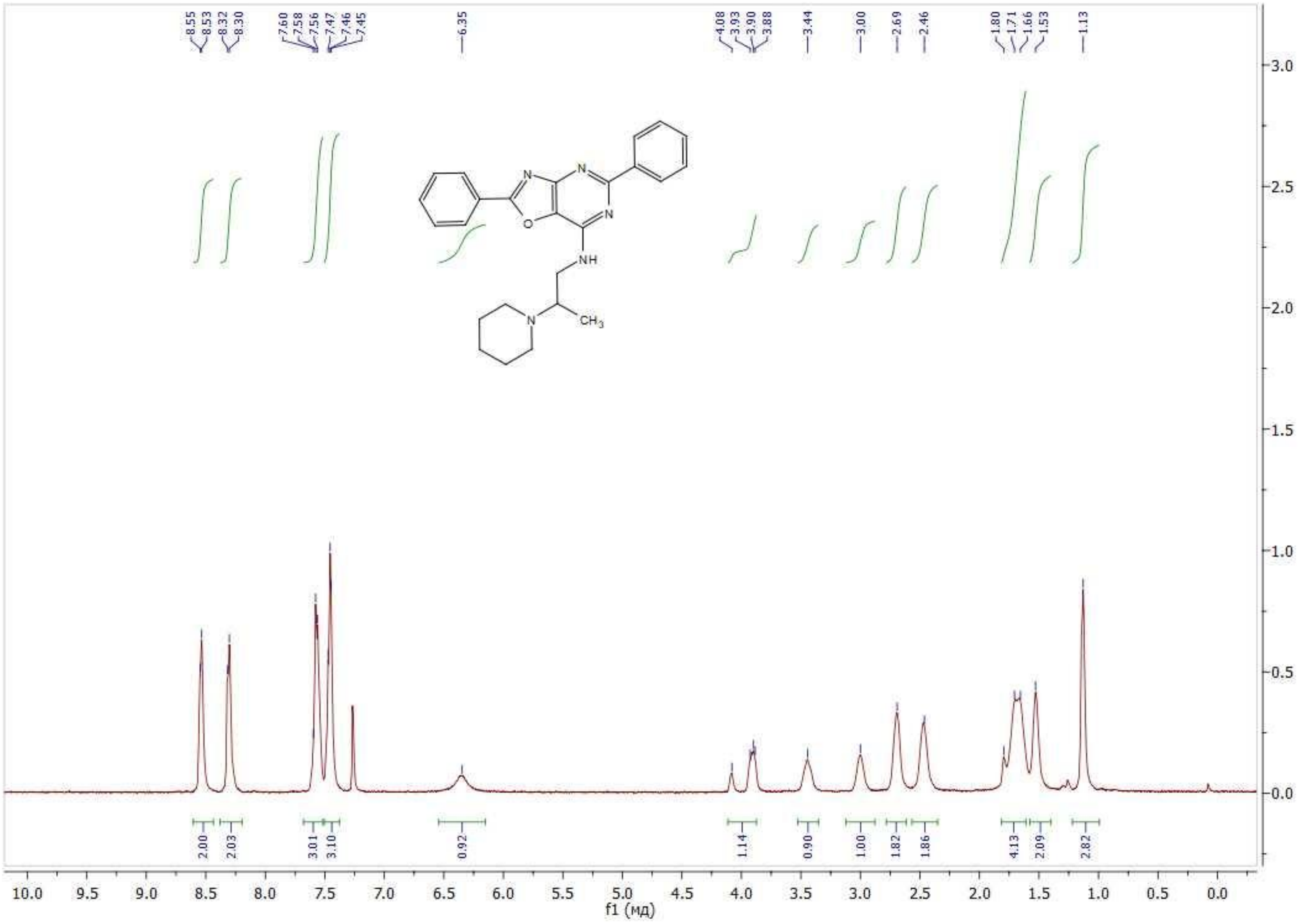

Figure S2. ^{1}H NMR spectrum of 2,5-diphenyl-*N*-(2-piperidin-1-ylpropyl)-[1,3]oxazolo[4,5-*d*]pyrimidin-7-amine (**1**)

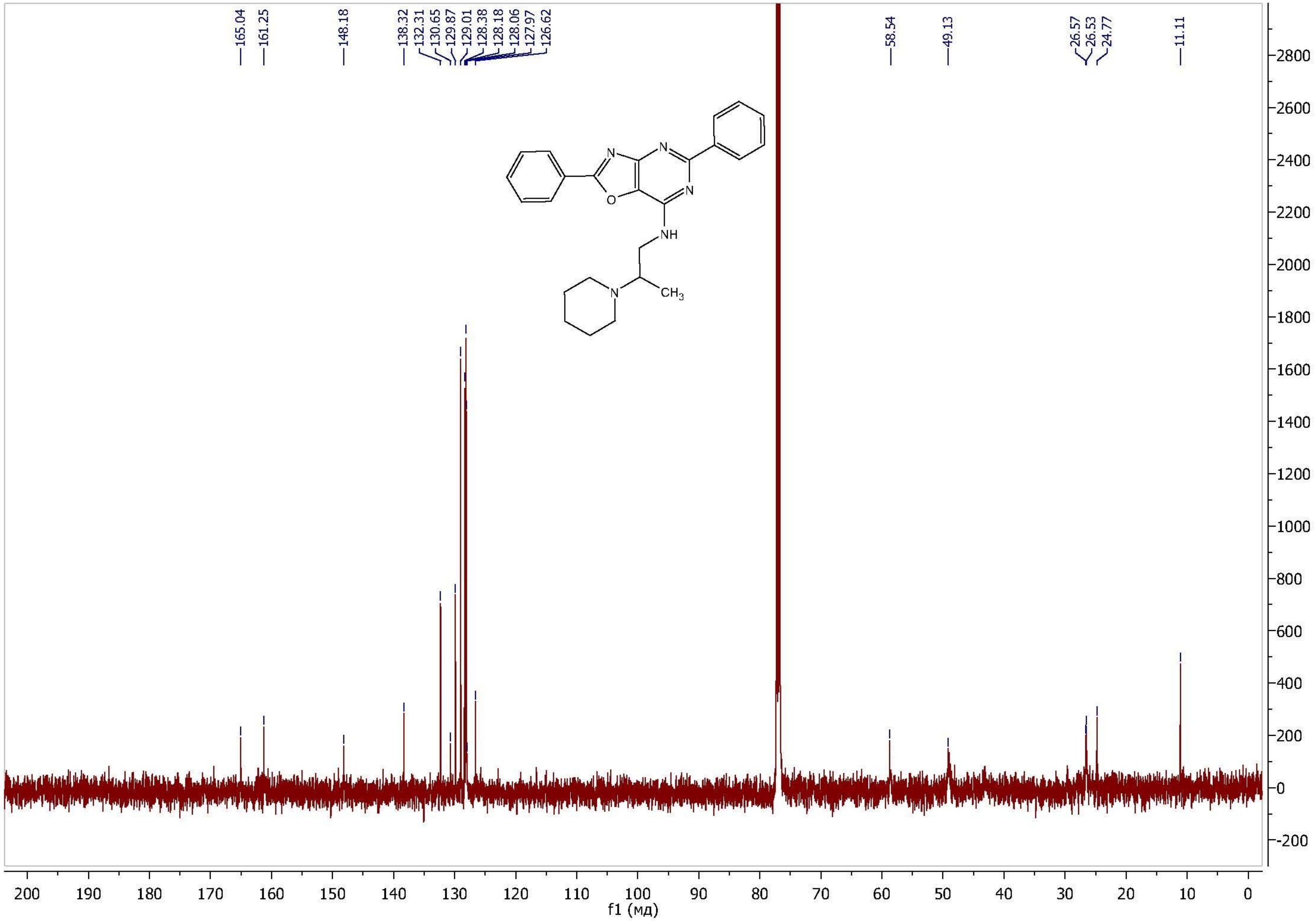

Figure S3. ^{13}C NMR spectrum of 2,5-diphenyl-*N*-(2-piperidin-1-ylpropyl)-[1,3]oxazolo[4,5-*d*]pyrimidin-7-amine (**1**)

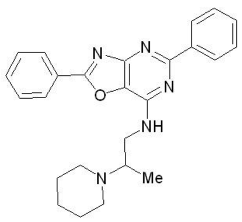

CLQ262119

L969949D
LCMS-27
SUPOR_30.M
19:42 26.08.2025
Product not found
No product

#	RT	DAD1A	DAD1B	MSD1	MSD2	ELSD	MSD1 ions	MSD1 rt	MSD2 ions	MSD2 rt	Info
1	1.216	96.7%	94.2%	79.3%	100.0%	---	414.2(97),207.6(3)	1.223	412.2(80),448.0(20)	1.223	
2	1.249	3.3%	---	---	---	---	---	---	---	---	
3	1.794	---	5.8%	20.7%	---	---	357.2(100)	1.800	---	---	

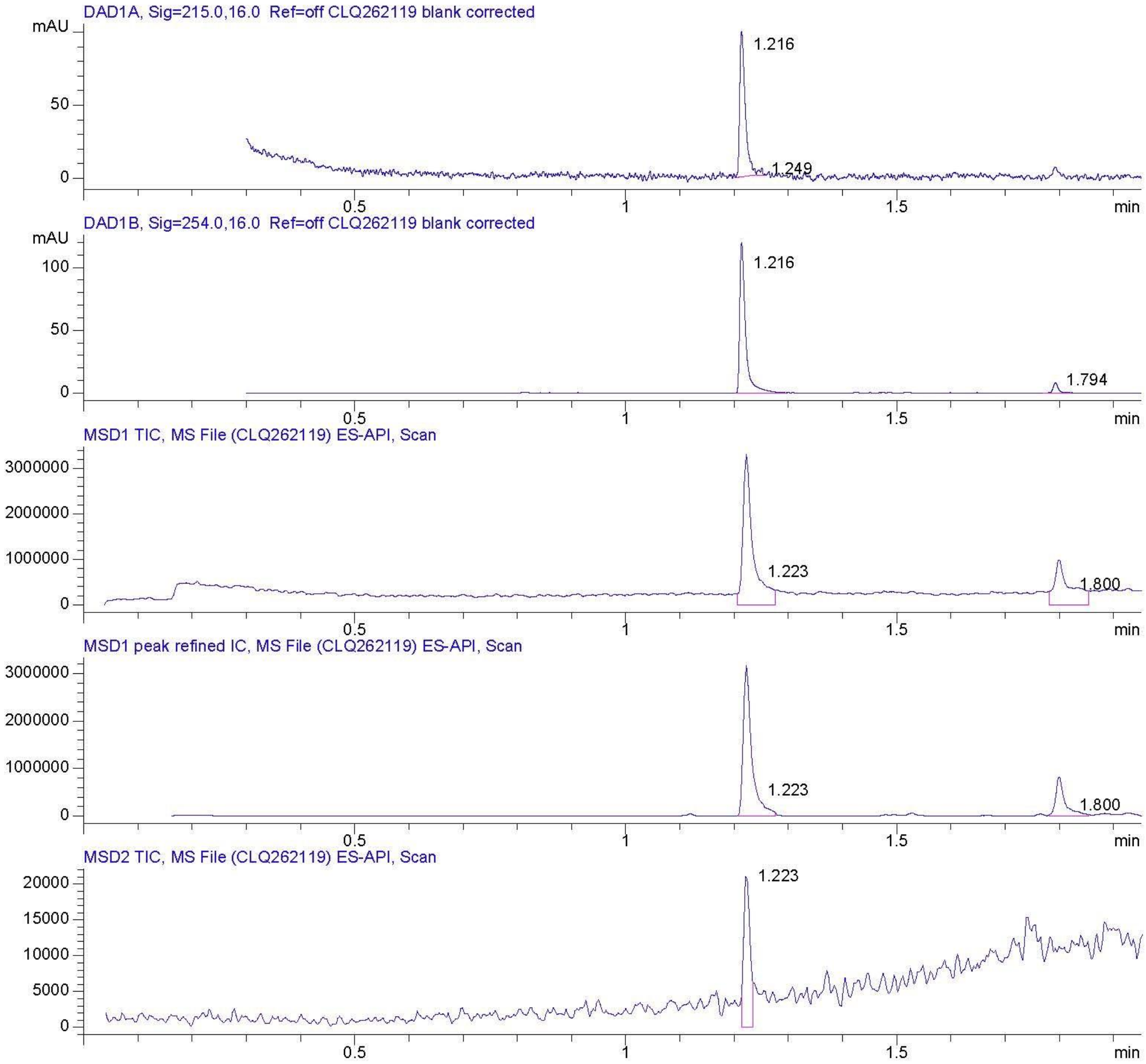

ELSD shift [illegible] | +1000 / -1000
MSD shift [illegible] | +120 / -120
f:\ABCH\2025\27\08\08_26\L969949D\001-NV-B.D
f:\ABCH\2025\27\08\08_26\L969949D\019-D6P-C1-CLQ262119.D
https://spectra.enamine.net/alpha?instrument=LCMS-27&acquiredDate=[illegible]&barcode=CLQ262119

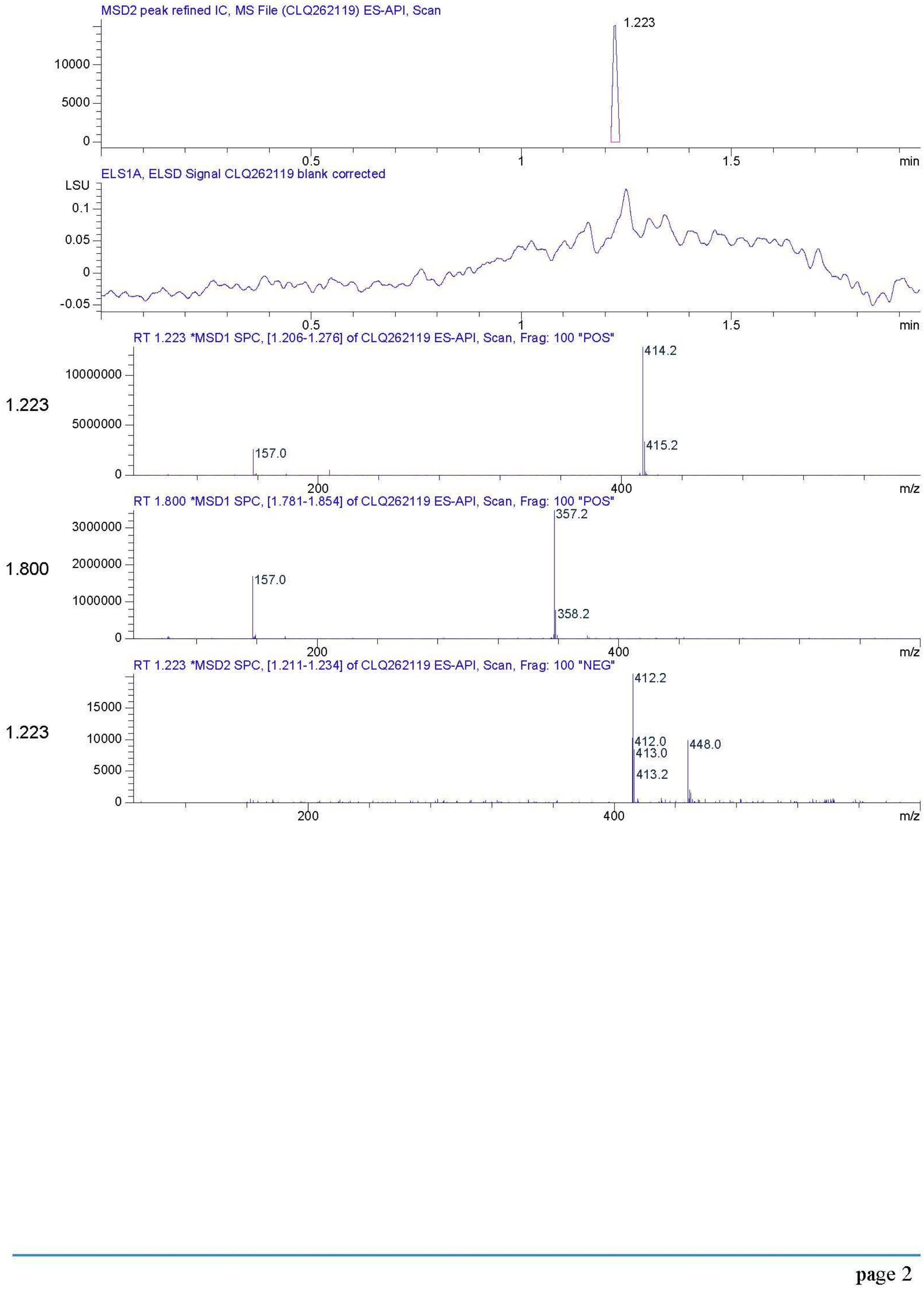

Figure S4. LC–MS spectrum of 2,5-diphenyl-*N*-(2-piperidin-1-ylpropyl)-[1,3]oxazolo[4,5-*d*]pyrimidin-7-amine (**1**)

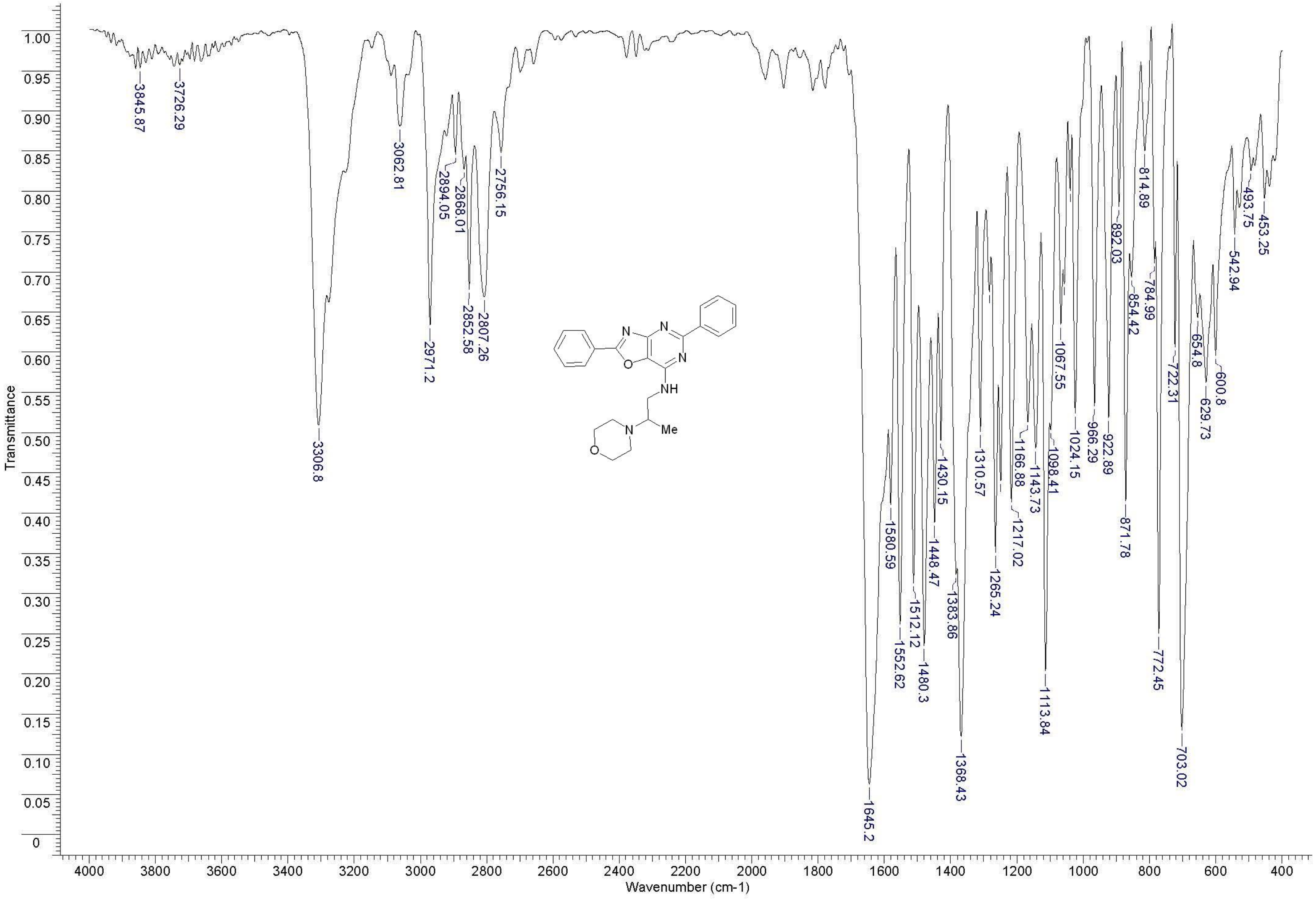

Figure S5. IR spectrum of *N*-(2-morpholin-4-ylpropyl)-2,5-diphenyl-[1,3]oxazolo[4,5-*d*]pyrimidin-7-amine (**2**)

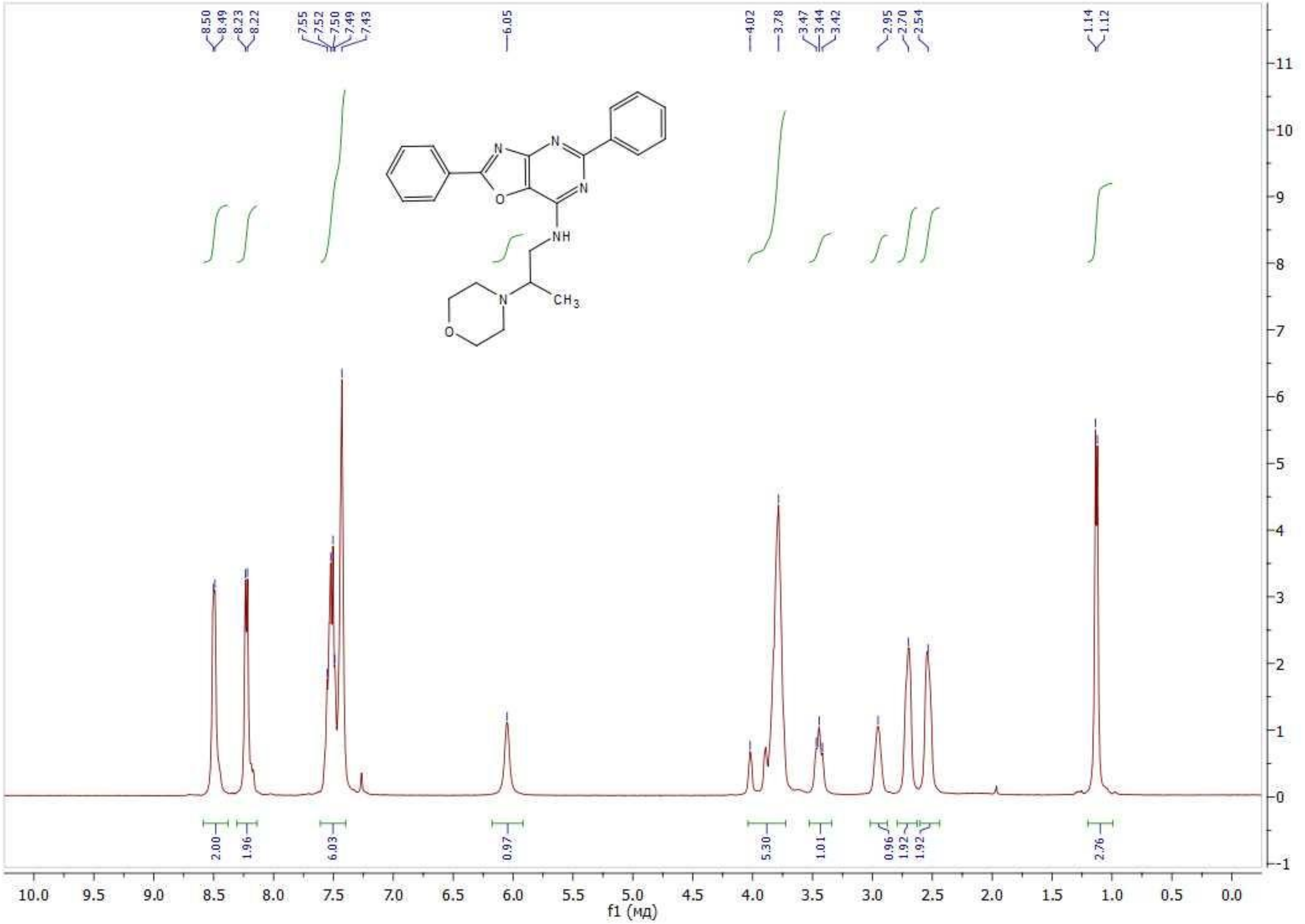

Figure S6. ^{1}H NMR spectrum of *N*-(2-morpholin-4-ylpropyl)-2,5-diphenyl-[1,3]oxazolo[4,5-*d*]pyrimidin-7-amine (**2**)

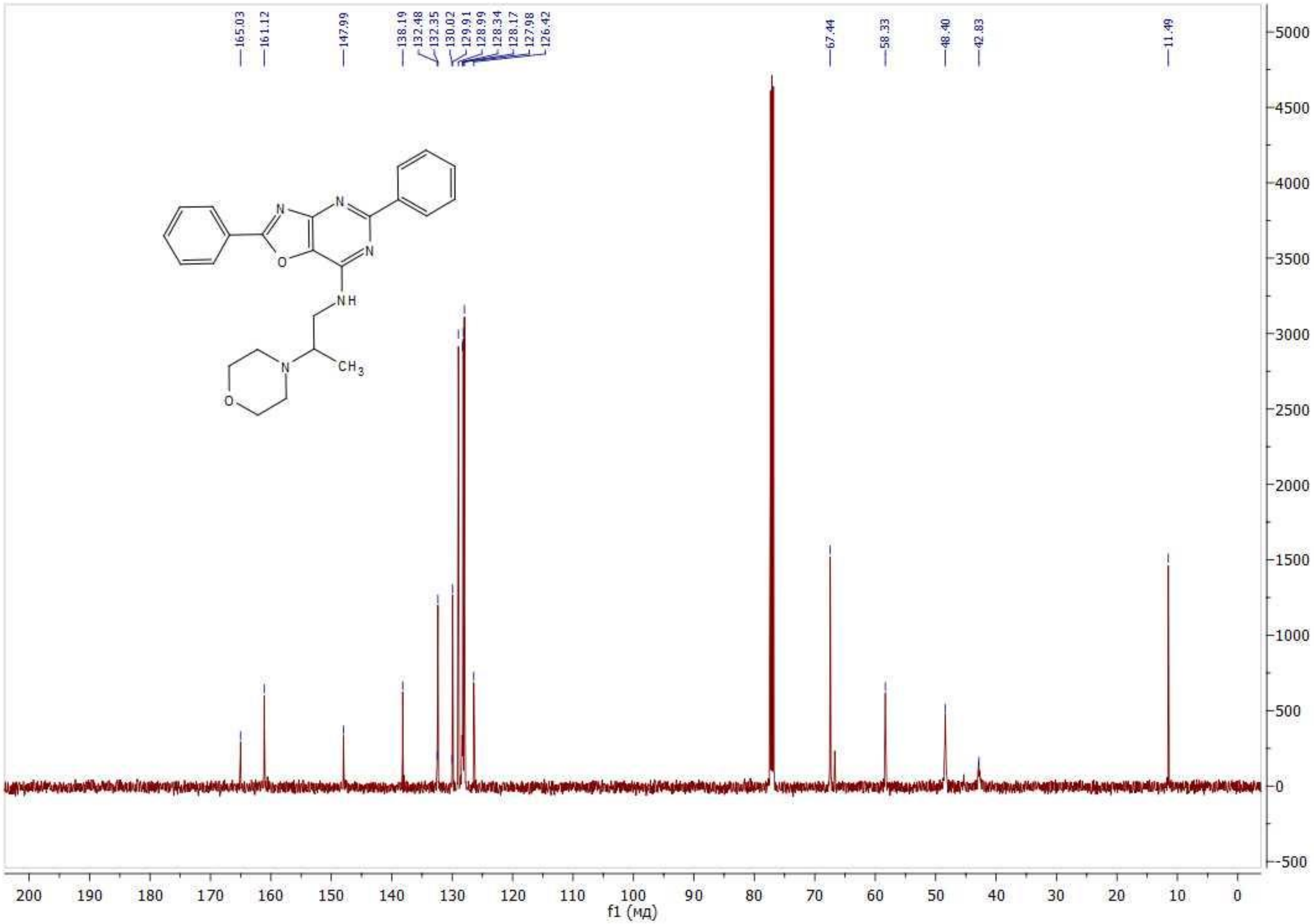

Figure S7. ^{13}C NMR spectrum of *N*-(2-morpholin-4-ylpropyl)-2,5-diphenyl-[1,3]oxazolo[4,5-*d*]pyrimidin-7-amine (**2**)

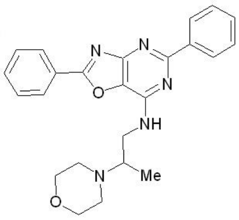

CLQ394866

L969949D
LCMS-27
SUPOR_30.M
19:33 26.08.2025
Product not found
No product

#	RT	DAD1A	DAD1B	MSD1	MSD2	ELSD	MSD1 ions	MSD1 rt	MSD2 ions	MSD2 rt	Info
1	1.140	93.2%	90.5%	83.5%	100.0%	100.0%	416.2(98),208.6(2)	1.145	414.2(50),450.2(50)	1.146	
2	1.194	---	1.8%	1.7%	---	---	430.2(100)	1.200	---	---	
3	1.588	6.8%	7.7%	14.9%	---	---	359.2(100)	1.594	---	---	

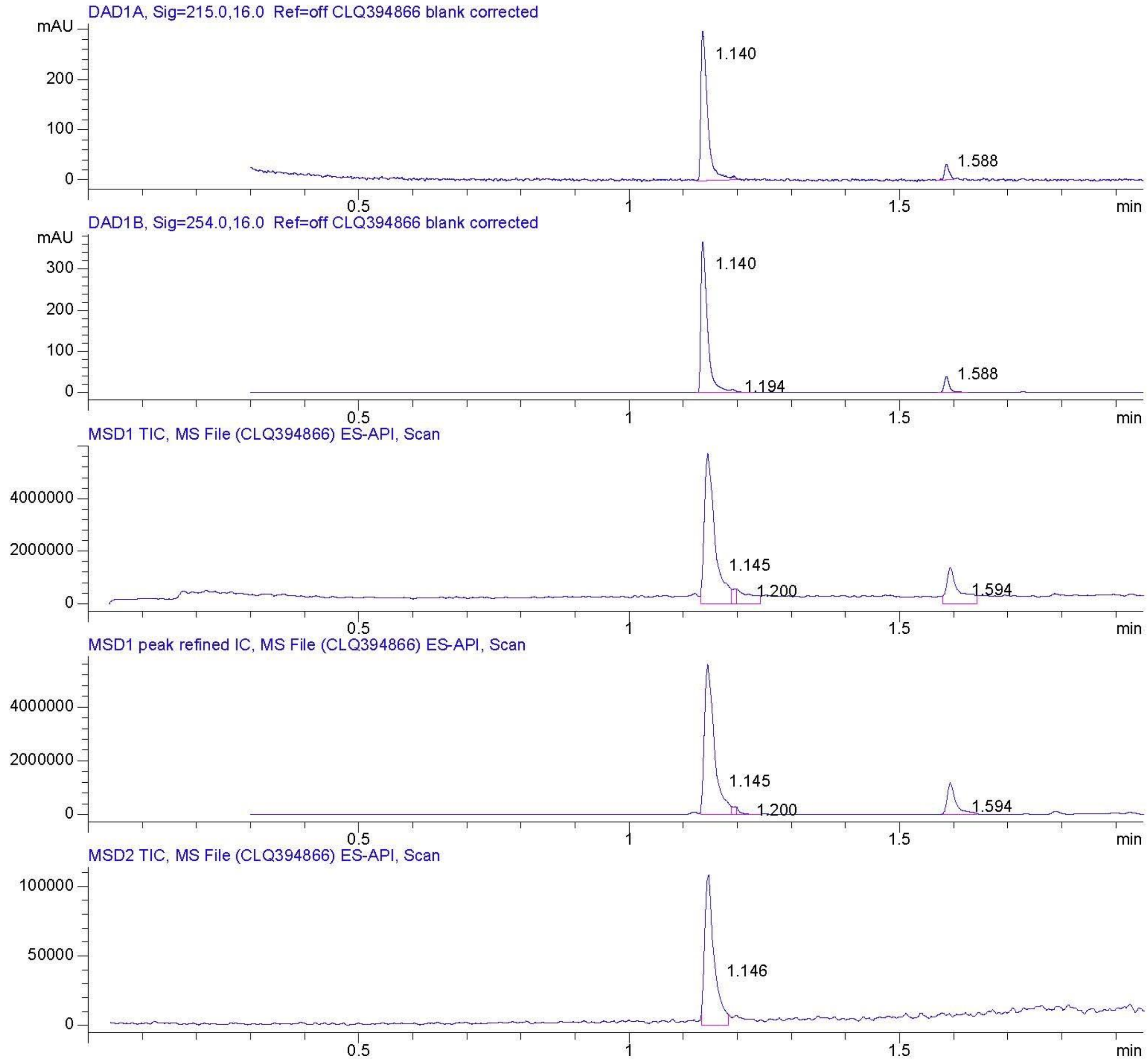

ELSD shift 2235 | +1000 / -1000
MSD shift 334 | +120 / -120
f:\ABCH\2025\27\08\08_26\L969949D\001-NV-B.D
f:\ABCH\2025\27\08\08_26\L969949D\016-D6F-B6-CLQ394866.D
https://spectra.enamine.net/alpha?instrument=LCMS-27&acquiredDate=1756226086000&barcode=CLQ394866

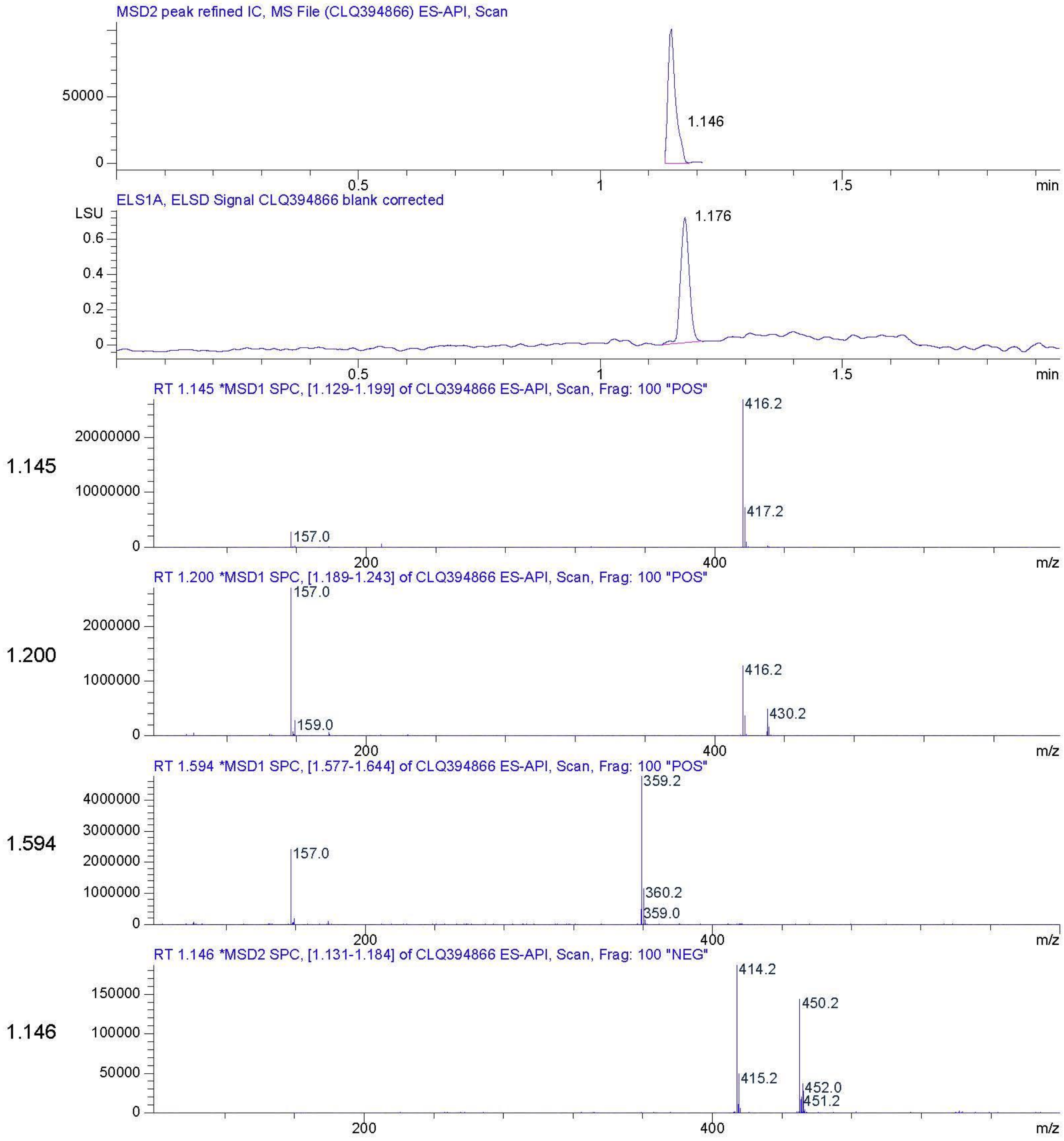

page 2

Figure S8. LC–MS spectrum of *N*-(2-morpholin-4-ylpropyl)-2,5-diphenyl-[1,3]oxazolo[4,5-*d*]pyrimidin-7-amine (**2**)

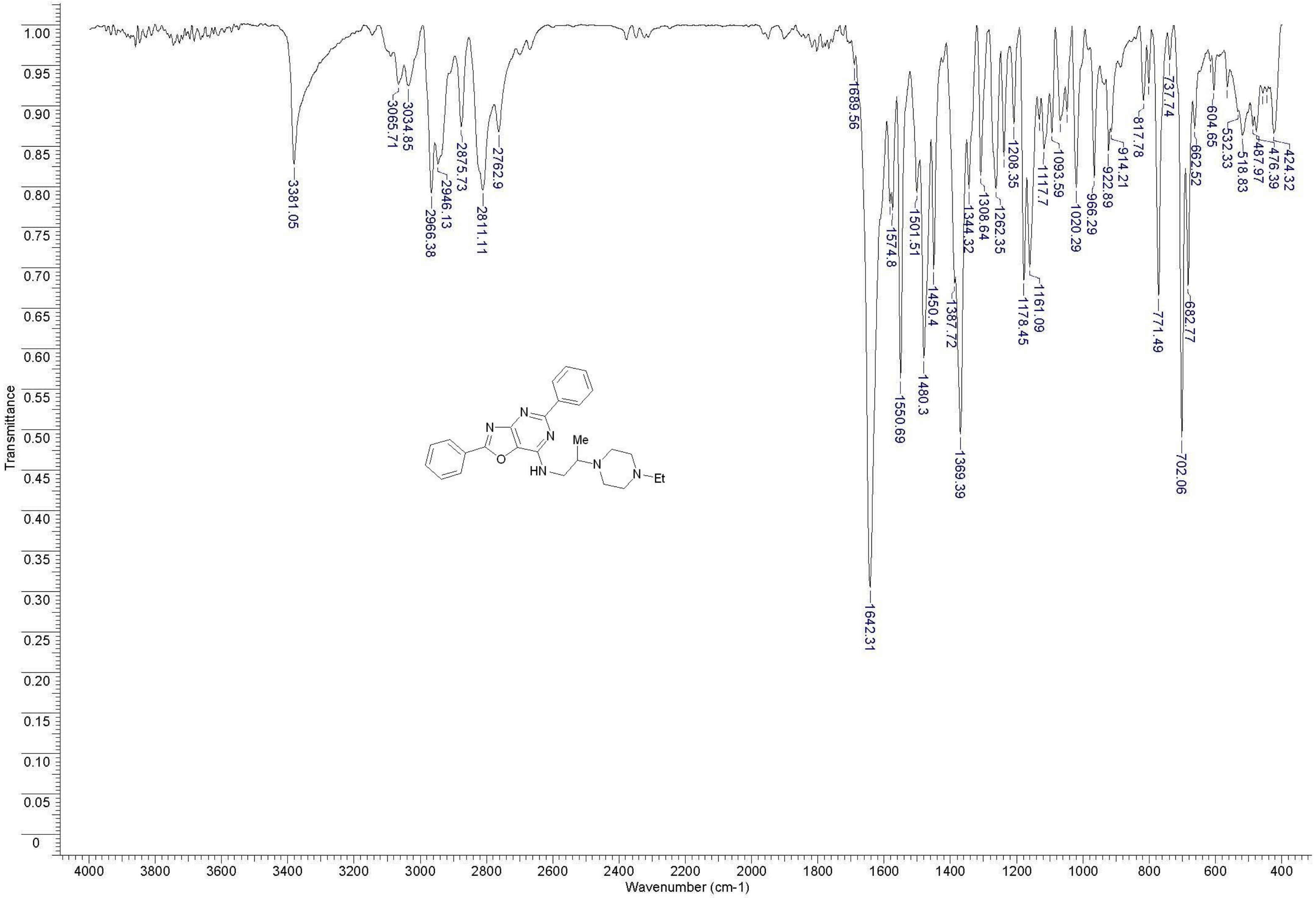

Figure S9. IR spectrum of *N*-[2-(4-ethylpiperazin-1-yl)propyl]-2,5-diphenyl-[1,3]oxazolo[4,5-*d*]pyrimidin-7-amine (**3**)

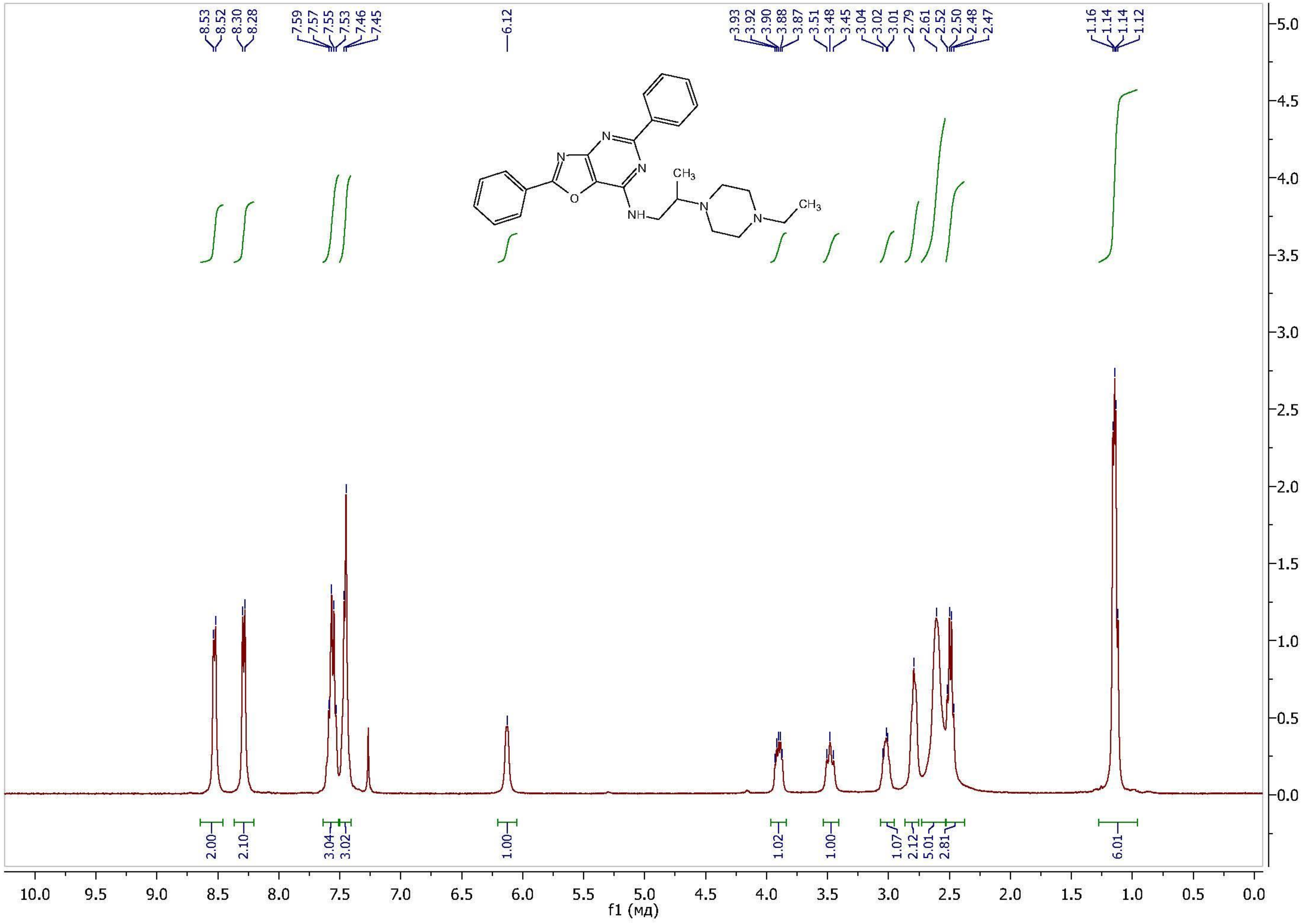

Figure S10. ^{1}H NMR spectrum of *N*-[2-(4-ethylpiperazin-1-yl)propyl]-2,5-diphenyl-[1,3]oxazolo[4,5-*d*]pyrimidin-7-amine (**3**)

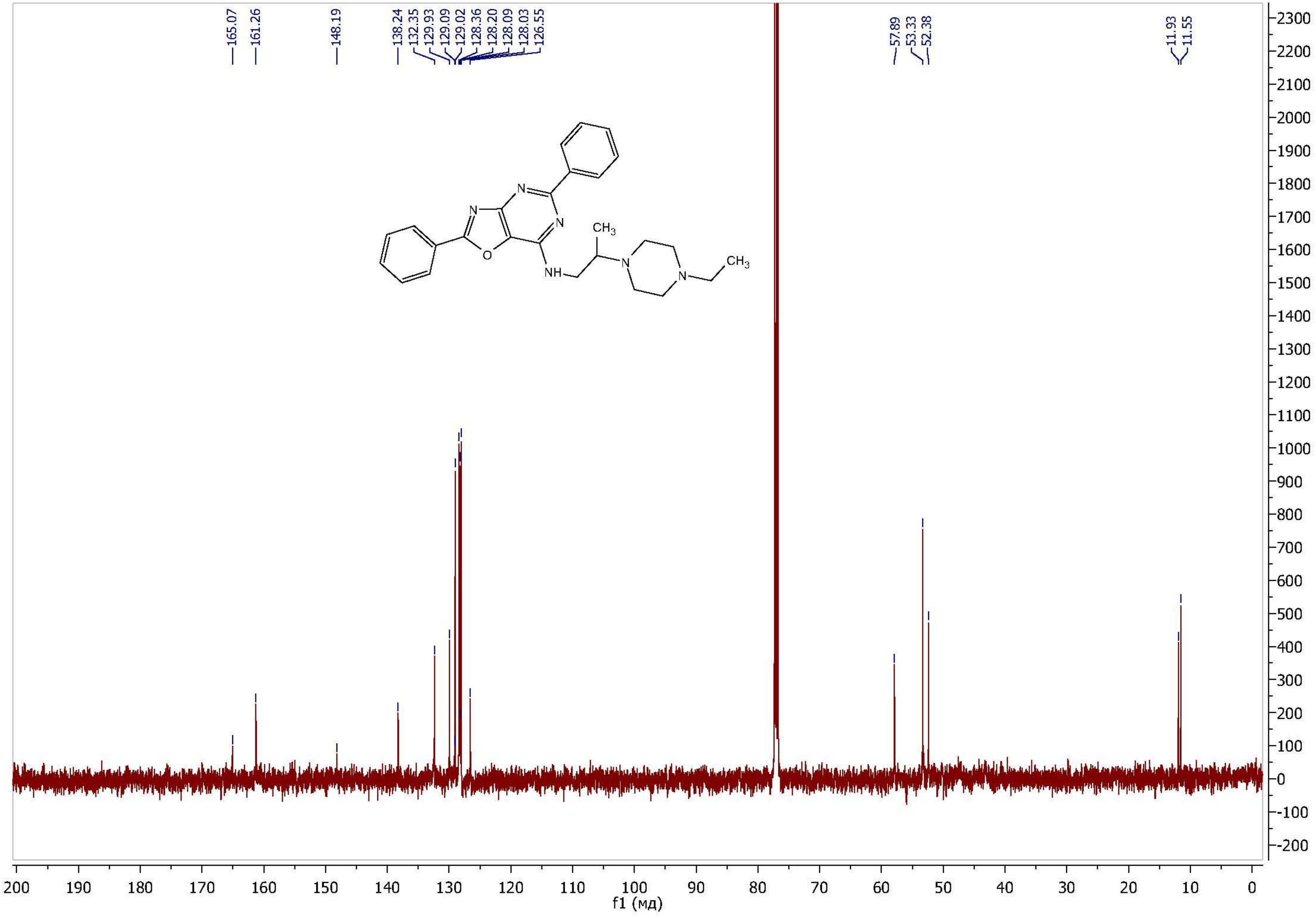

Figure S11. ^{13}C NMR spectrum of *N*-[2-(4-ethylpiperazin-1-yl)propyl]-2,5-diphenyl-[1,3]oxazolo[4,5-*d*]pyrimidin-7-amine (**3**)

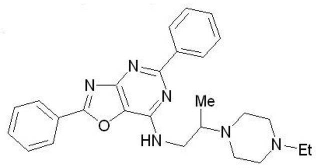

CLQ394867

L969949D
LCMS-27
SUPOR_30.M
19:36 26.08.2025
Product not found
No product

#	RT	DAD1A	DAD1B	MSD1	MSD2	ELSD	MSD1 ions	MSD1 rt	MSD2 ions	MSD2 rt	Info
1	1.200	---	---	---	17.7%	---	---	---	477.2(100)	1.206	
2	1.202	100.0%	100.0%	100.0%	71.8%	100.0%	443.2(66),222.2(34)	1.207	441.2(87),479.0(13)	1.206	
3	1.210	---	---	---	10.5%	---	---	---	442.2(56),478.0(44)	1.216	

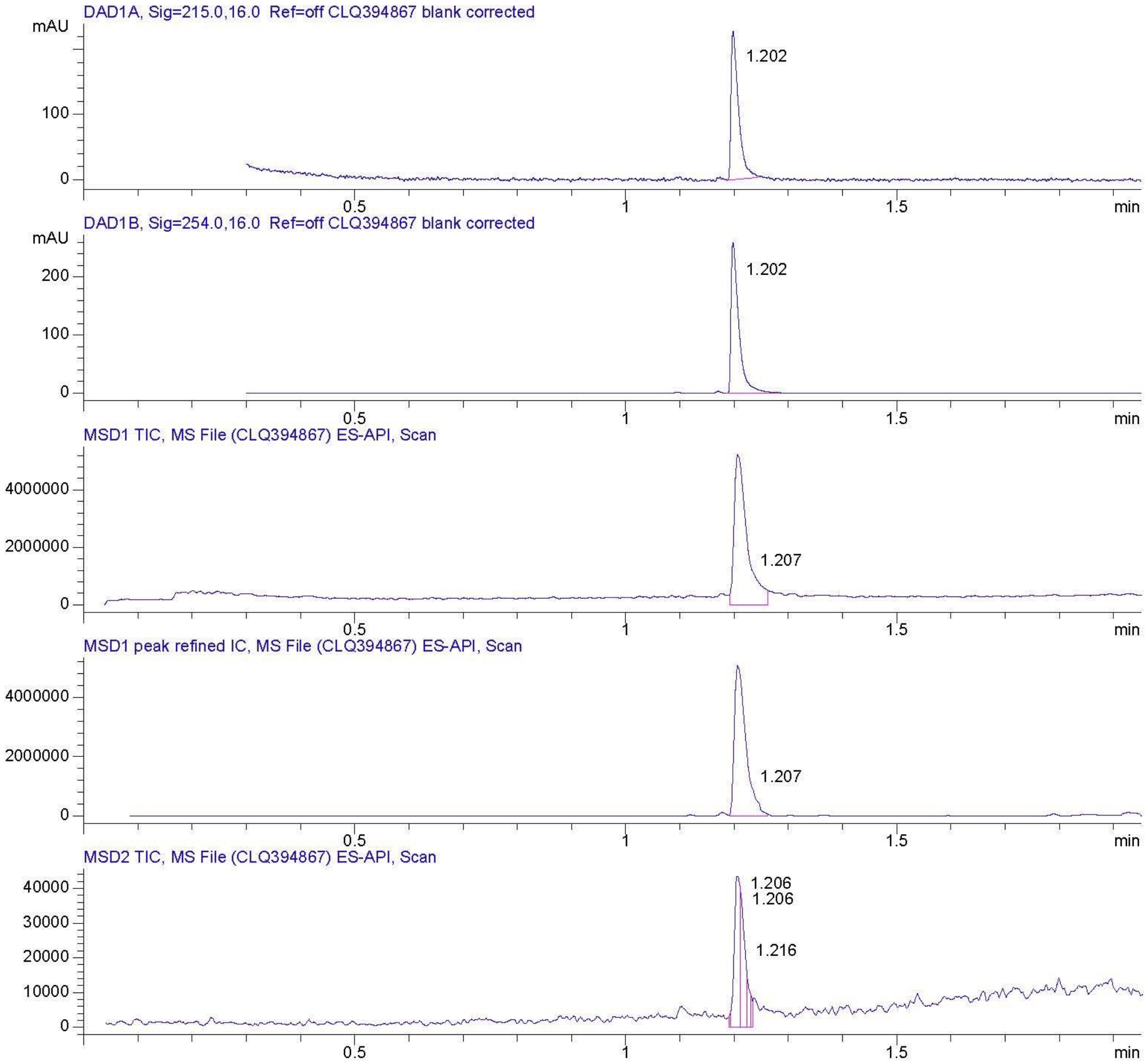

ELSD shift 225.7 | +1000 /-1000
MSD shift 3.29 | +120 /-120
f:\ABCH\2025\27\08\08_26\L969949D\001-NV-B.D
f:\ABCH\2025\27\08\08_26\L969949D\017-D6F-B7-CLQ394867.D
https://spectra.enamine.net/alpha?instrument=LCMS-27&acquiredDate=1756226211000&barcode=CLQ394867

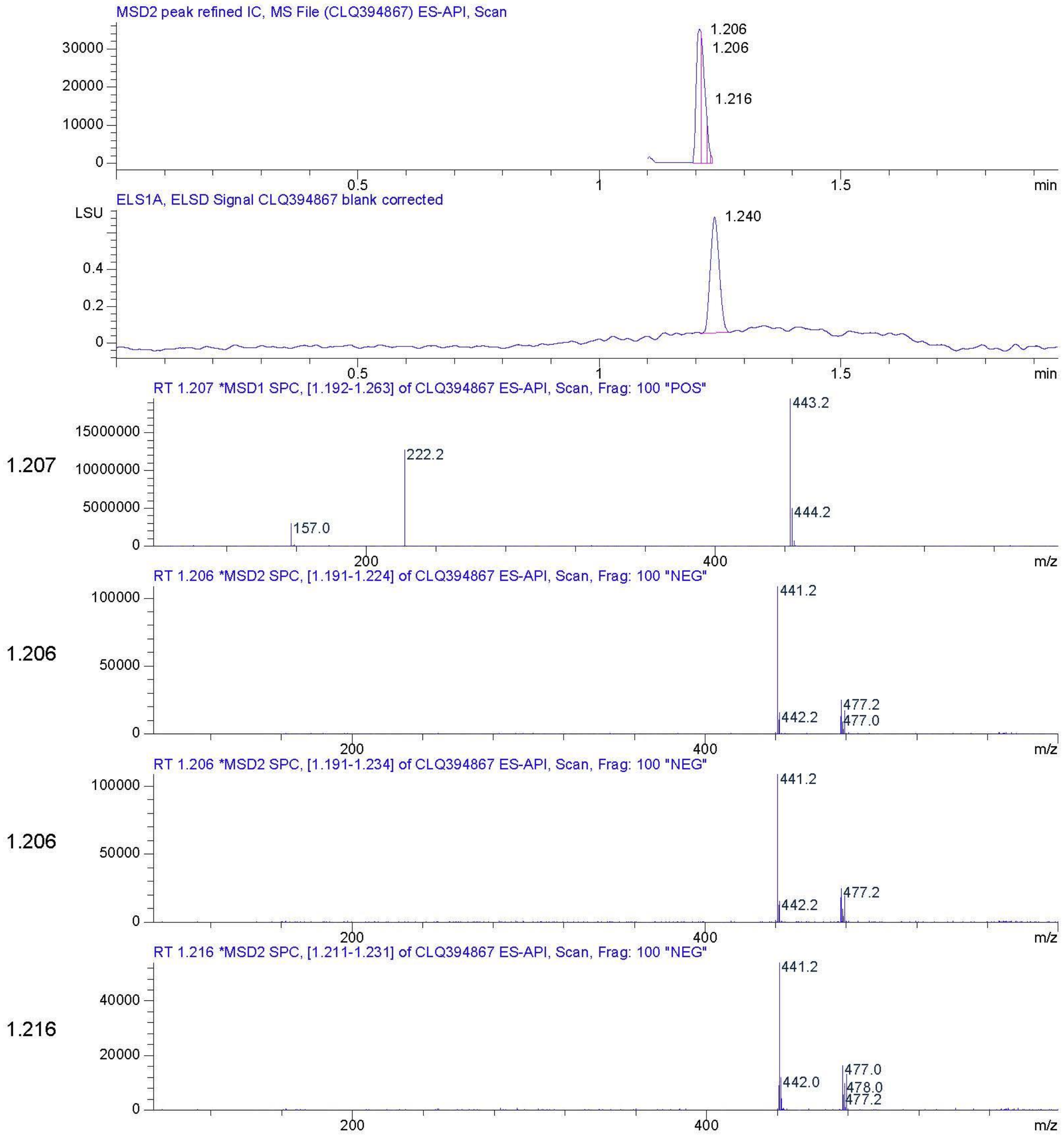

page 2

Figure S12. LC–MS spectrum of *N*-[2-(4-ethylpiperazin-1-yl)propyl]-2,5-diphenyl-[1,3]oxazolo[4,5-*d*]pyrimidin-7-amine (**3**)

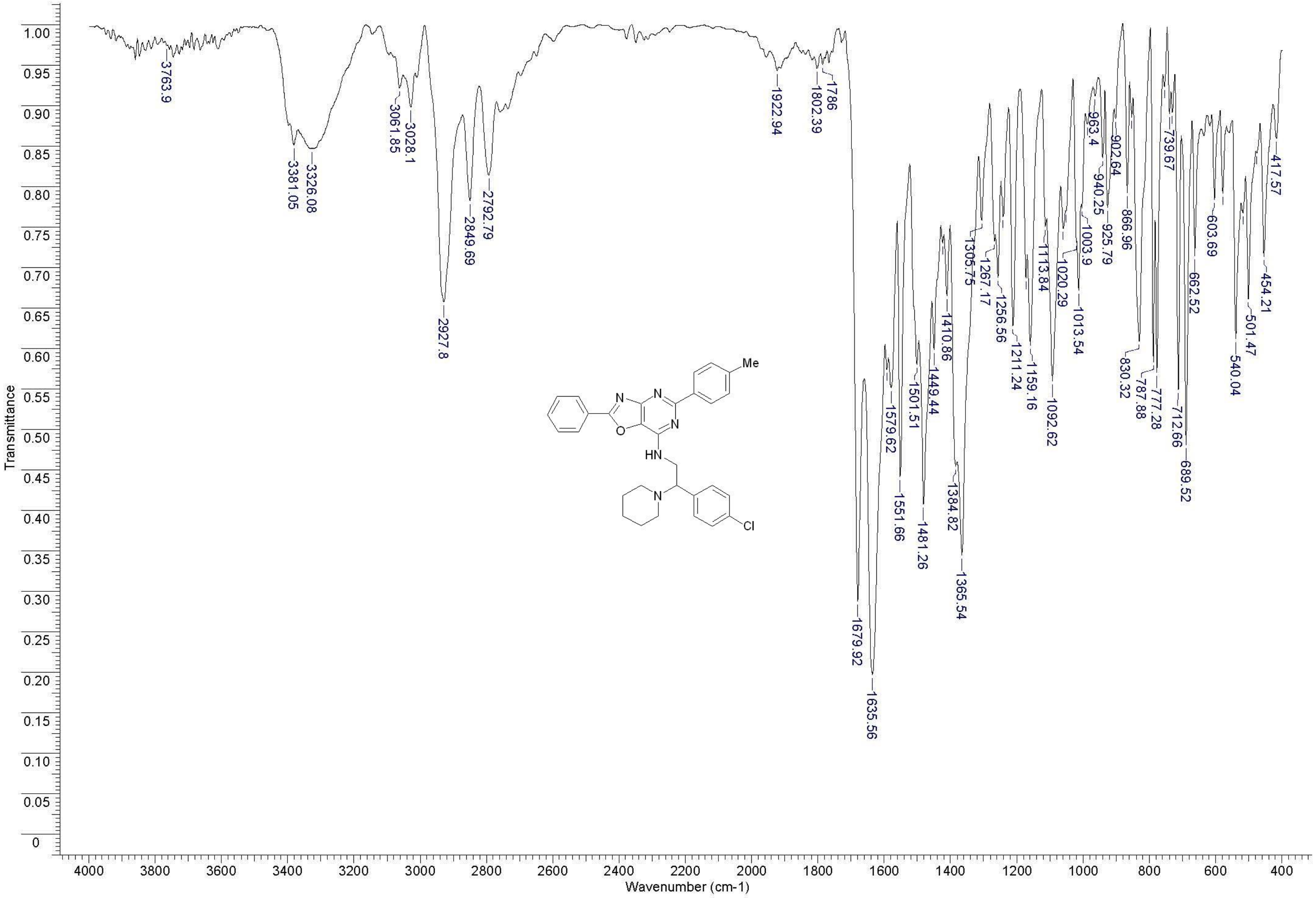

Figure S13. IR spectrum of *N*-[2-(4-chlorophenyl)-2-piperidin-1-ylethyl]-5-(4-methylphenyl)-2-phenyl-[1,3]oxazolo[4,5-*d*]pyrimidin-7-amine (**4**)

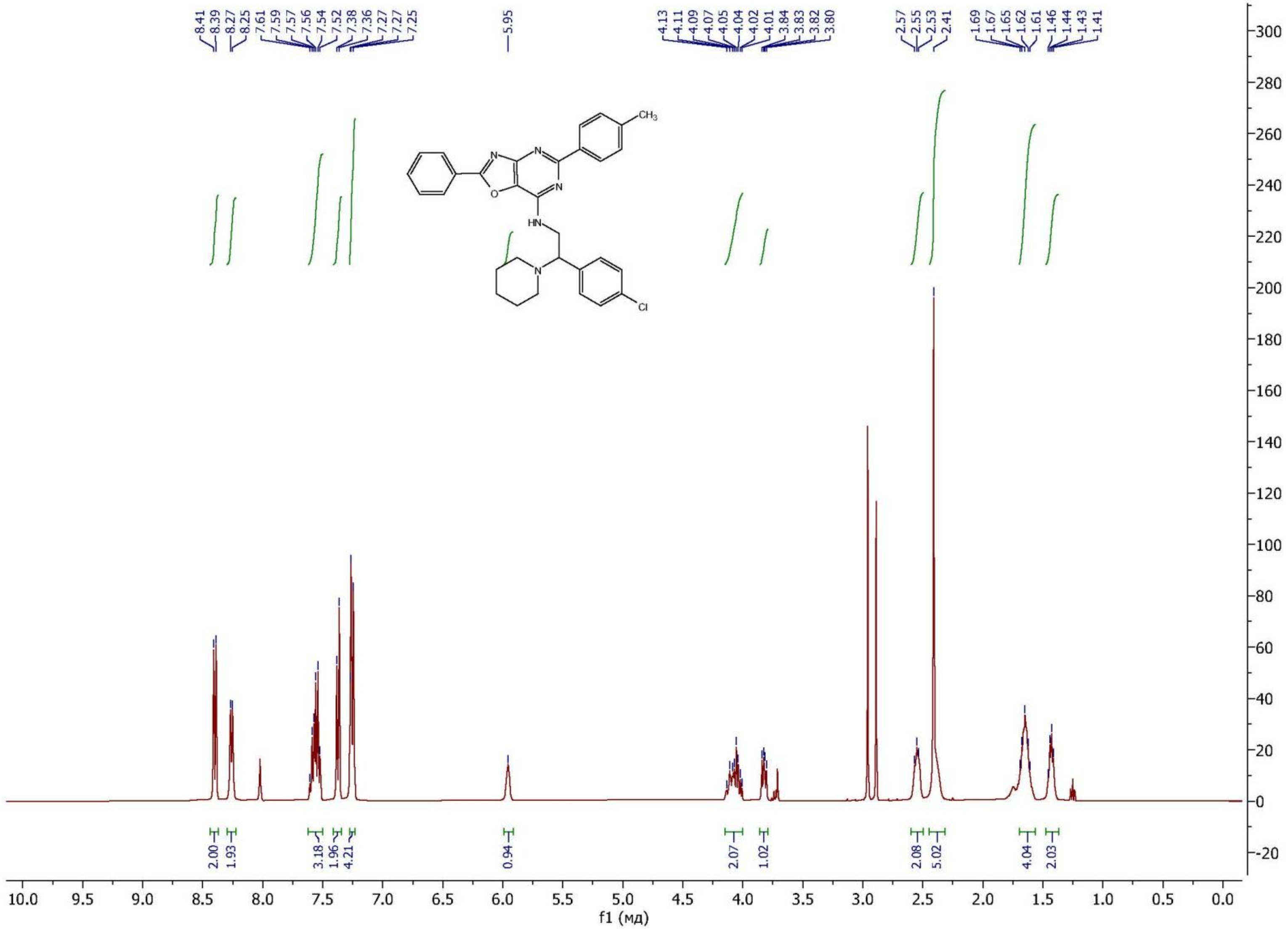

Figure S14. ^{1}H NMR spectrum of *N*-[2-(4-chlorophenyl)-2-piperidin-1-ylethyl]-5-(4-methylphenyl)-2-phenyl-[1,3]oxazolo[4,5-*d*]pyrimidin-7-amine (**4**)

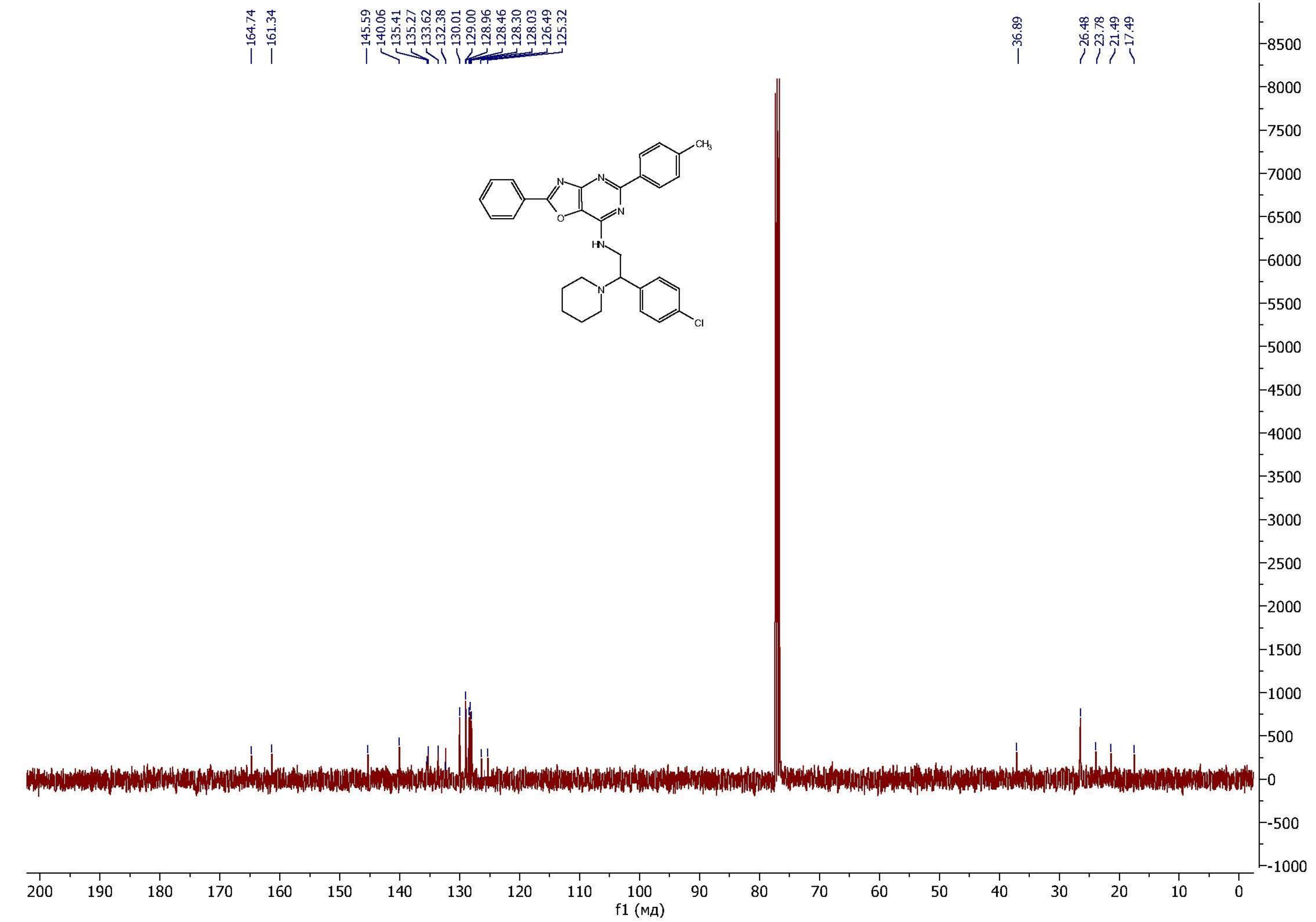

Figure S15. ^{13}C NMR spectrum of *N*-[2-(4-chlorophenyl)-2-piperidin-1-ylethyl]-5-(4-methylphenyl)-2-phenyl-[1,3]oxazolo[4,5-*d*]pyrimidin-7-amine (**4**)

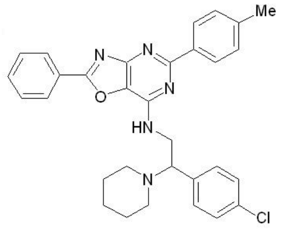

CLQ394868

L969947
LCMS-27
SUPOR_30.M
19:32 26.08.2025
Product not found
No product

#	RT	DAD1A	DAD1B	MSD1	MSD2	ELSD	MSD1 ions	MSD1 rt	MSD2 ions	MSD2 rt	Info
1	1.511	95.8%	93.2%	92.1%	41.9%	100.0%	525.2(88),528.2(7),263.6(5)	1.518	560.2(52),562.2(48)	1.518	
2	1.514	---	---	---	58.1%	---	---	---	524.2(100)	1.520	
3	1.652	4.4%	5.4%	7.2%	---	---	373.2(100)	1.656	---	---	
4	1.792	---	1.1%	0.7%	---	---	322.0(100)	1.798	---	---	

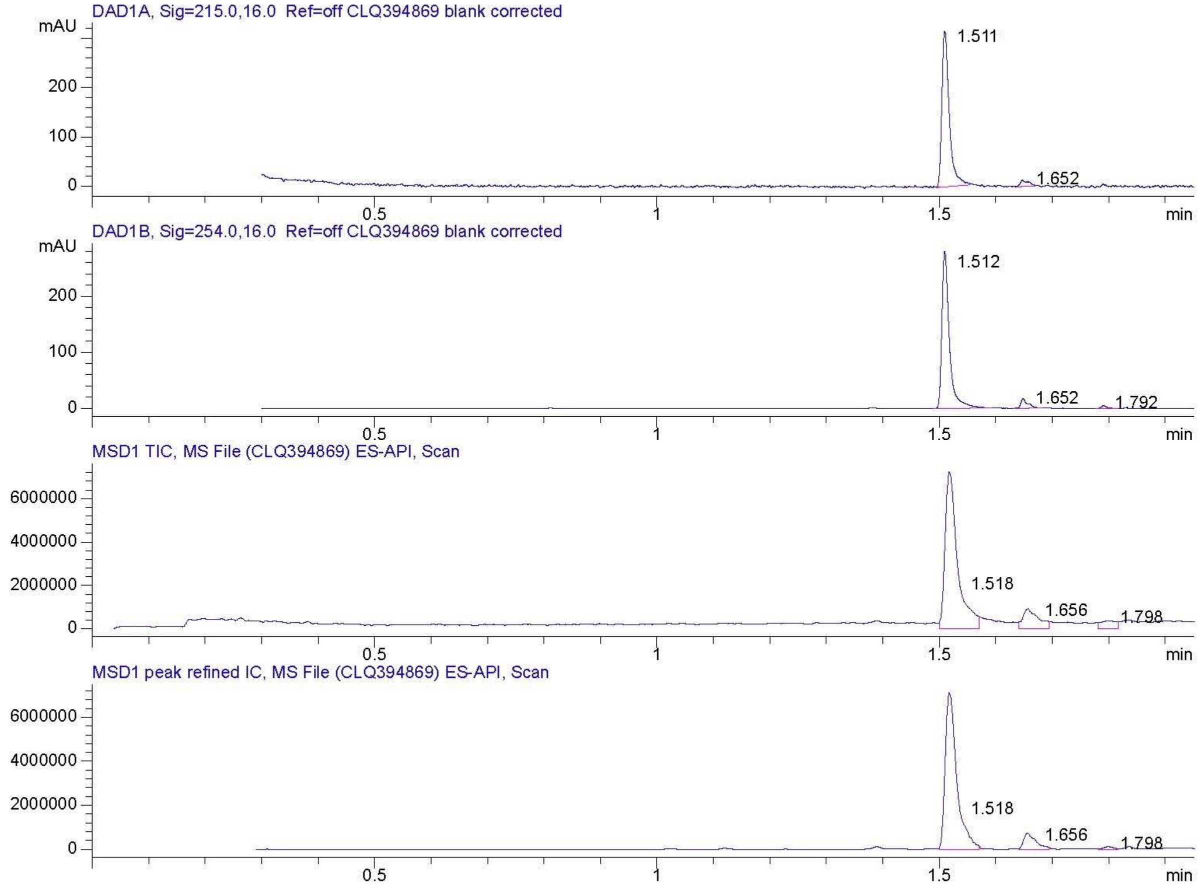

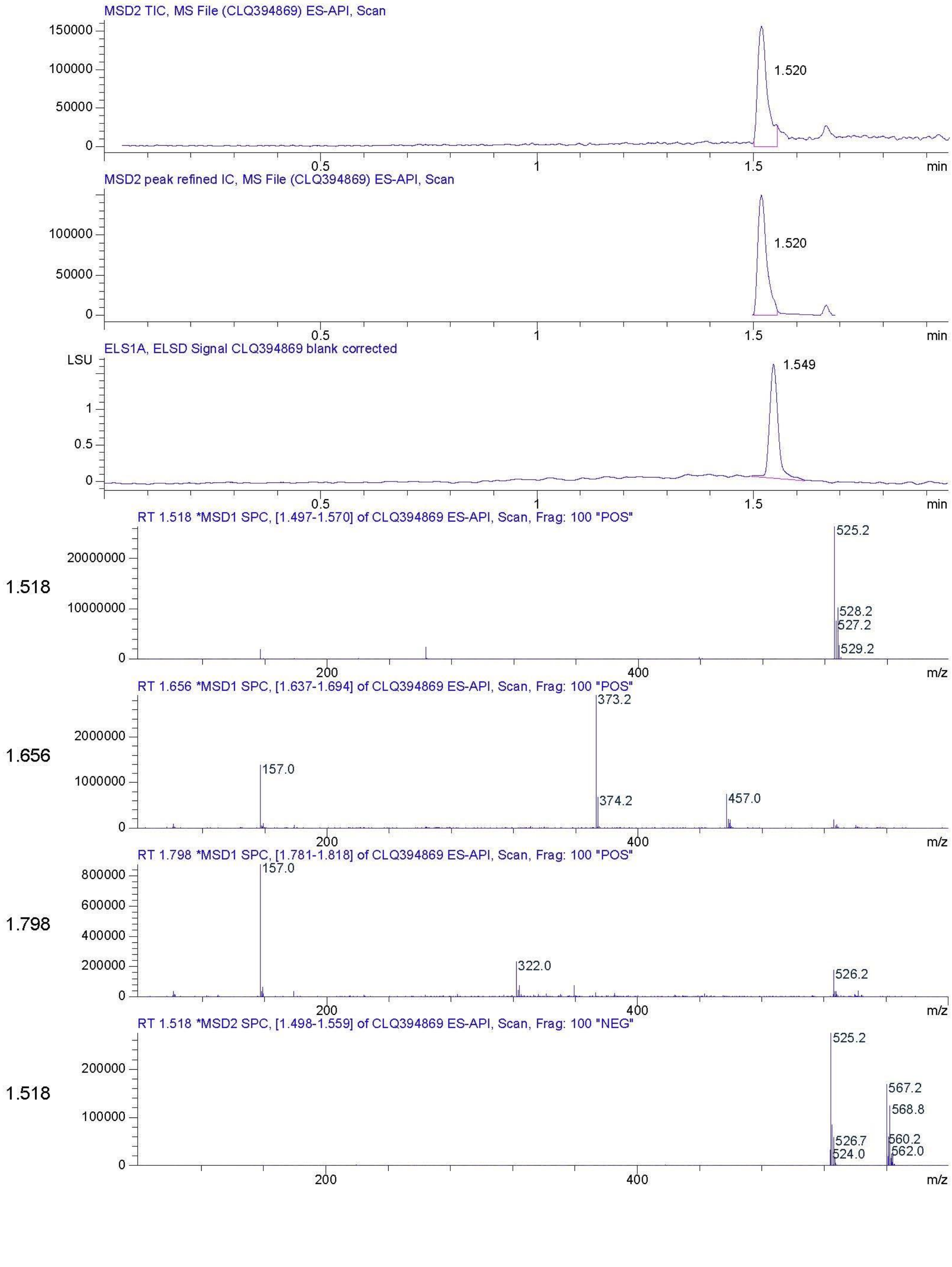
MSD2 TIC, MS File (CLQ394869) ES-API, Scan
150000
100000
50000
0
1.520
0.5
1
1.5
min
MSD2 peak refined IC, MS File (CLQ394869) ES-API, Scan
100000
50000
0
1.520
0.5
1
1.5
min
ELS1A, ELSD Signal CLQ394869 blank corrected
LSU
1
0.5
0
1.549
0.5
1
1.5
min
RT 1.518 *MSD1 SPC, [1.497-1.570] of CLQ394869 ES-API, Scan, Frag: 100 "POS"
1.518
20000000
10000000
0
525.2
528.2
527.2
529.2
200
400
m/z
RT 1.656 *MSD1 SPC, [1.637-1.694] of CLQ394869 ES-API, Scan, Frag: 100 "POS"
1.656
2000000
1000000
0
373.2
157.0
374.2
457.0
200
400
m/z
RT 1.798 *MSD1 SPC, [1.781-1.818] of CLQ394869 ES-API, Scan, Frag: 100 "POS"
1.798
800000
600000
400000
200000
0
157.0
322.0
526.2
200
400
m/z
RT 1.518 *MSD2 SPC, [1.498-1.559] of CLQ394869 ES-API, Scan, Frag: 100 "NEG"
1.518
200000
100000
0
525.2
567.2
568.8
526.7
524.0
560.2
562.0
200
400
m/z

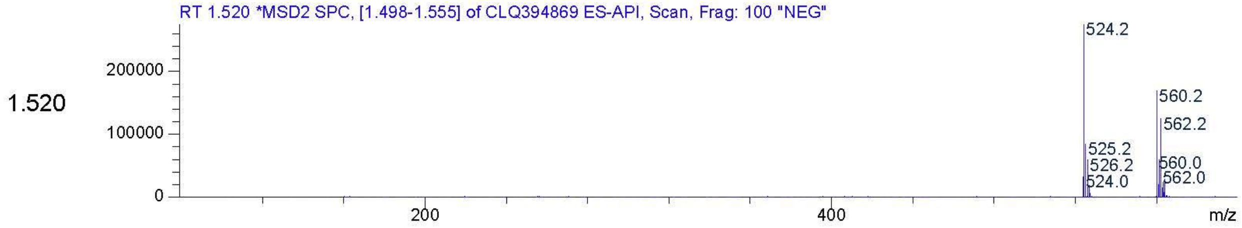

page 3

Figure S16. LC–MS spectrum of *N*-[2-(4-chlorophenyl)-2-piperidin-1-ylethyl]-5-(4-methylphenyl)-2-phenyl-[1,3]oxazolo[4,5-*d*]pyrimidin-7-amine (**4**)

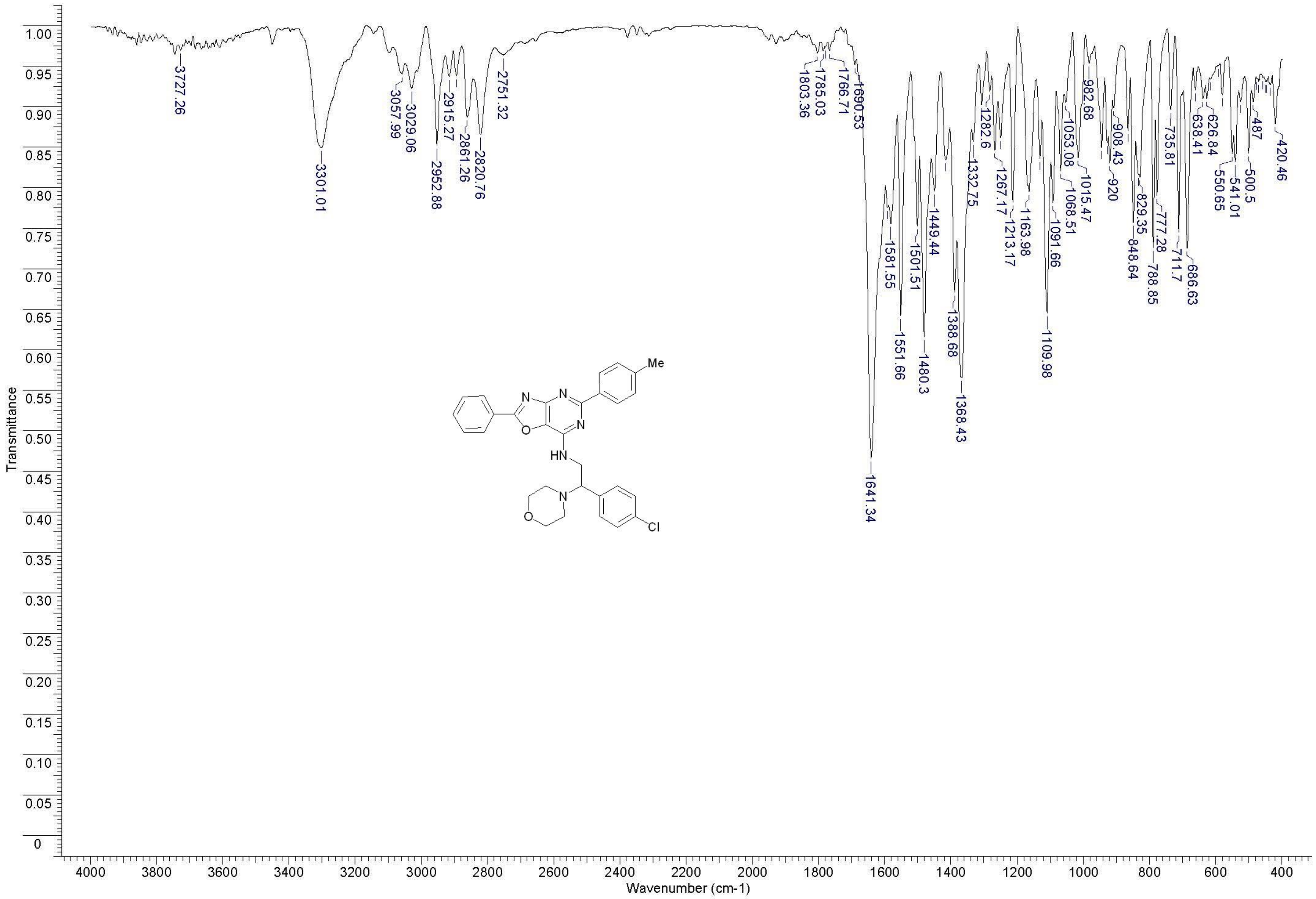

Figure S17. IR spectrum of *N*-[2-(4-chlorophenyl)-2-morpholin-4-ylethyl]-5-(4-methylphenyl)-2-phenyl[1,3]oxazolo[4,5-*d*]pyrimidin-7-amine (**5**)

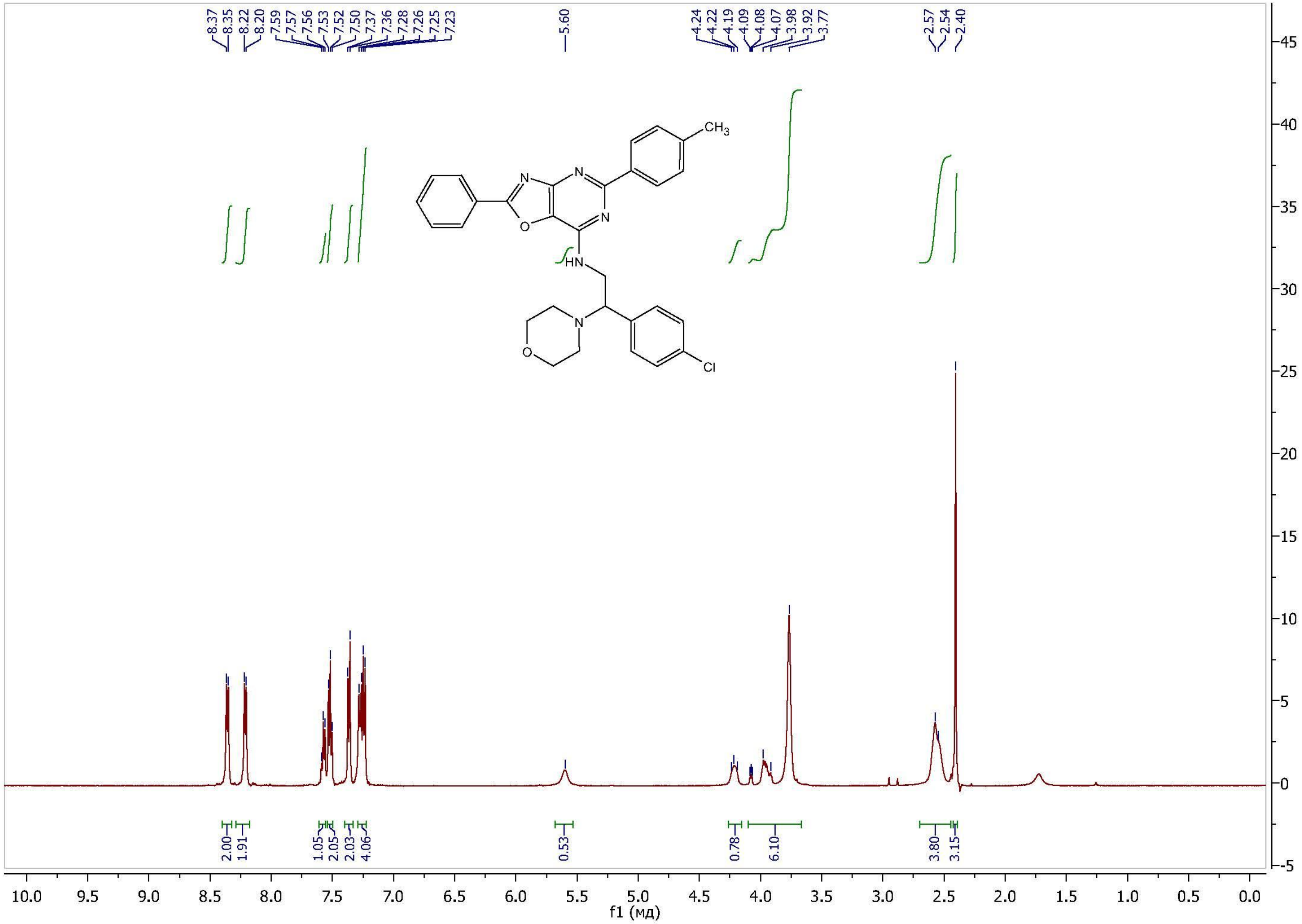

Figure S18. ^{1}H NMR spectrum of *N*-[2-(4-chlorophenyl)-2-morpholin-4-ylethyl]-5-(4-methylphenyl)-2-phenyl[1,3]oxazolo[4,5-*d*]pyrimidin-7-amine (**5**)

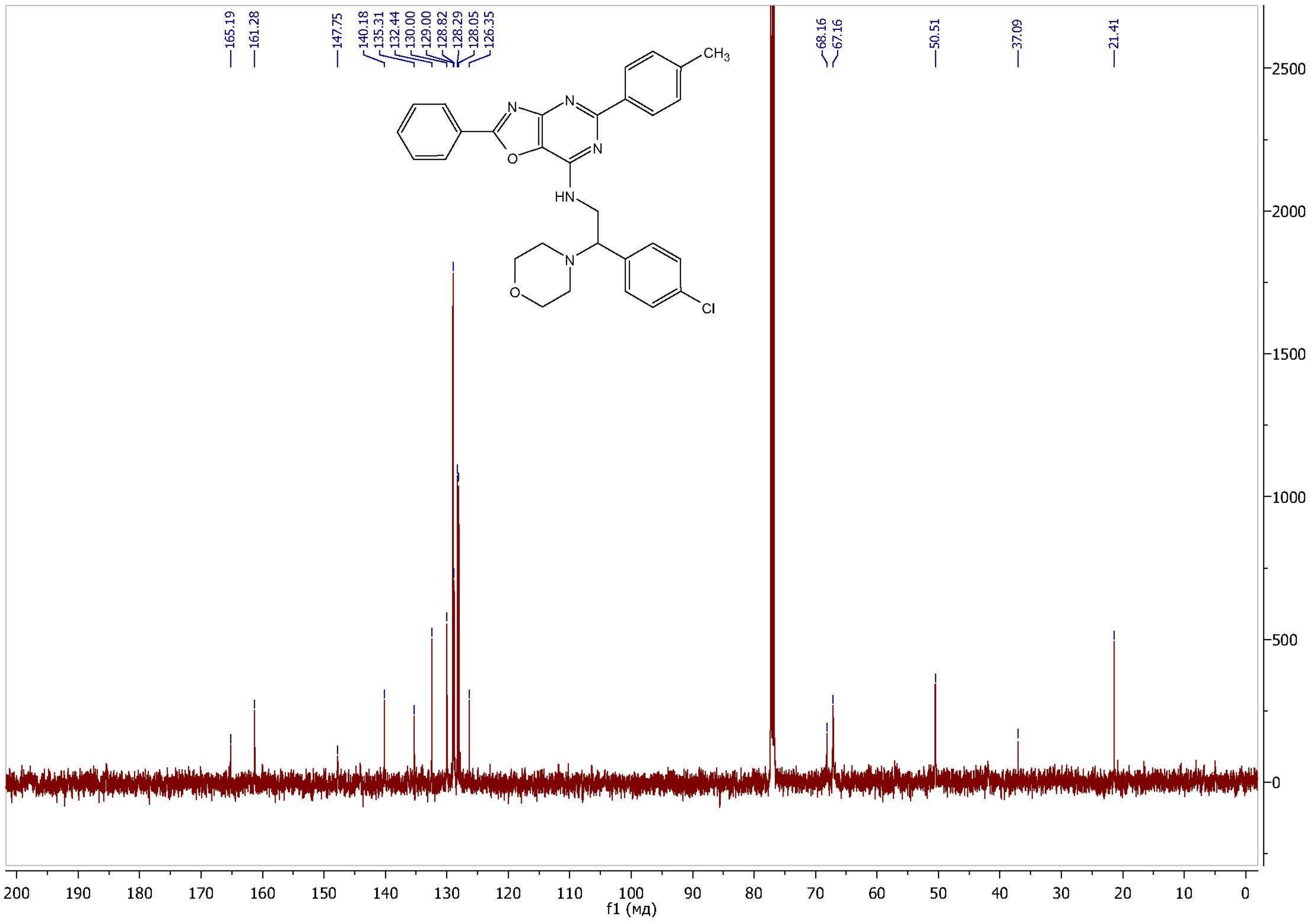

Figure S19. ^{13}C NMR spectrum of *N*-[2-(4-chlorophenyl)-2-morpholin-4-ylethyl]-5-(4-methylphenyl)-2-phenyl[1,3]oxazolo[4,5-*d*]pyrimidin-7-amine (**5**)

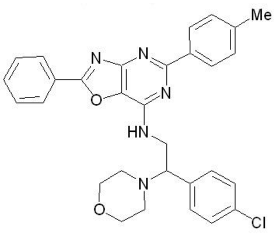

CLQ394869

L969949D
LCMS-27
SUPOR_30.M
19:39 26.08.2025
Product not found
No product

#	RT	DAD1A	DAD1B	MSD1	MSD2	ELSD	MSD1 ions	MSD1 rt	MSD2 ions	MSD2 rt	Info
1	1.512	95.6%	93.4%	92.1%	41.9%	100.0%	526.2(88),529.2(7),263.6(5)	1.518	560.2(52),562.2(48)	1.518	
2	1.514	---	---	---	58.1%	---	---	---	524.2(100)	1.520	
3	1.652	4.4%	5.4%	7.2%	---	---	373.2(100)	1.656	---	---	
4	1.792	---	1.1%	0.7%	---	---	322.0(100)	1.798	---	---	

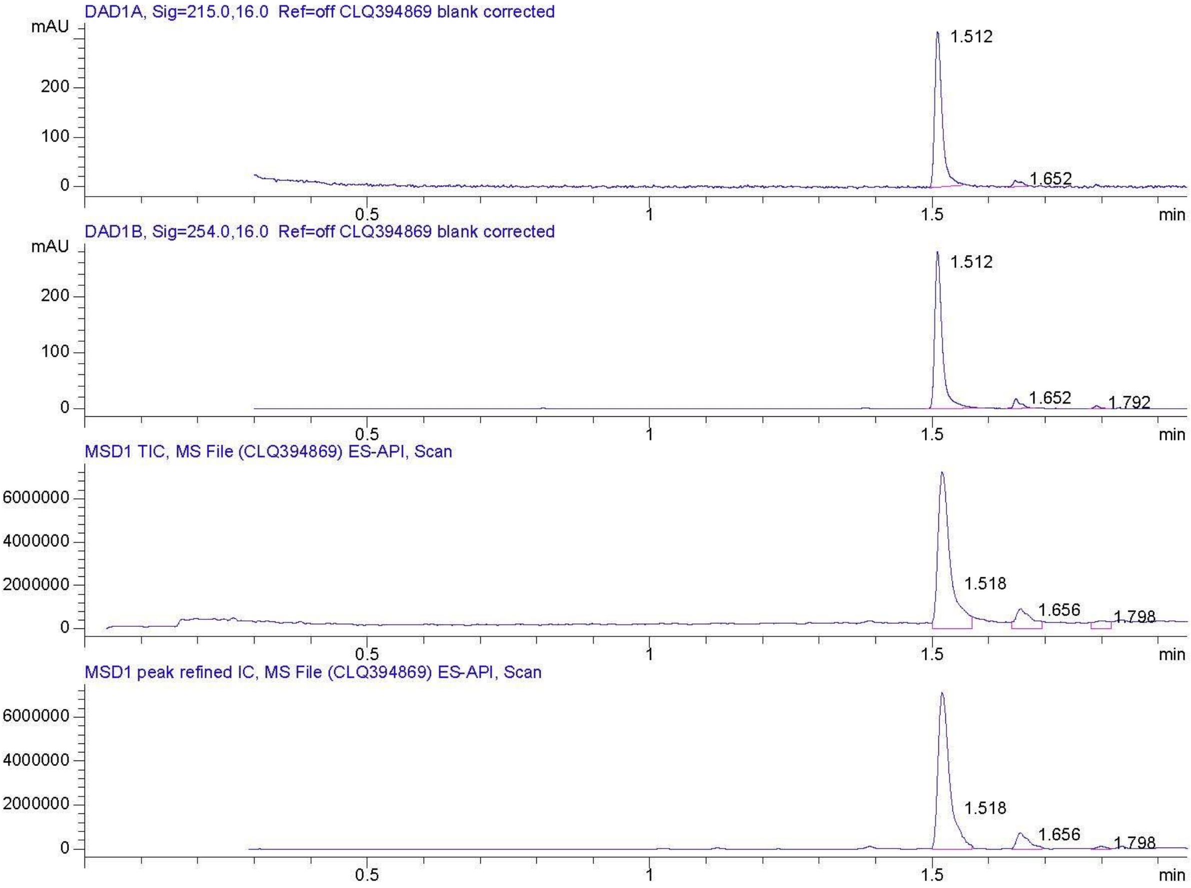

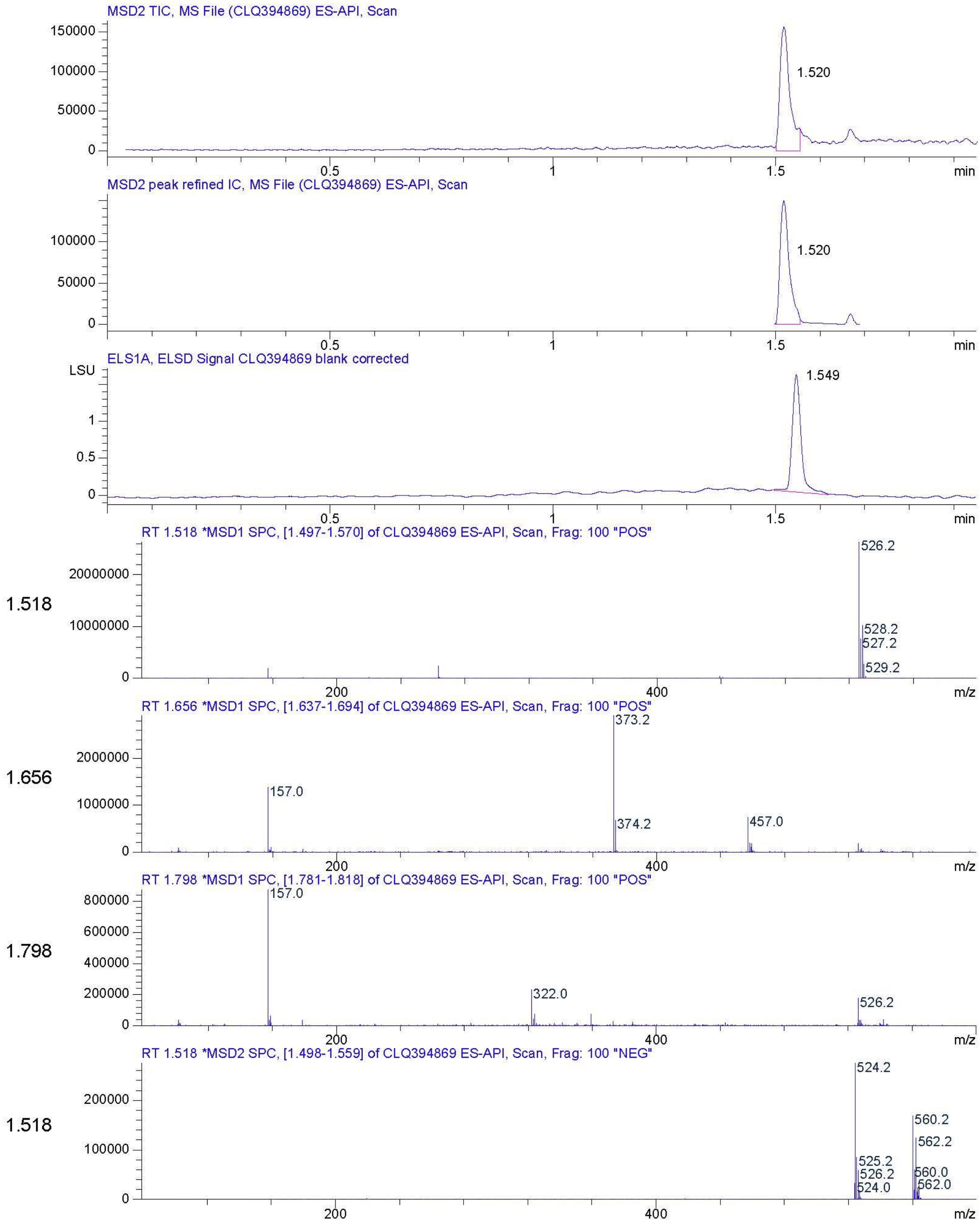
MSD2 TIC, MS File (CLQ394869) ES-API, Scan
1.520
MSD2 peak refined IC, MS File (CLQ394869) ES-API, Scan
1.520
ELS1A, ELSD Signal CLQ394869 blank corrected
LSU
1.549
min
RT 1.518 *MSD1 SPC, [1.497-1.570] of CLQ394869 ES-API, Scan, Frag: 100 "POS"
1.518
526.2
528.2
527.2
529.2
RT 1.656 *MSD1 SPC, [1.637-1.694] of CLQ394869 ES-API, Scan, Frag: 100 "POS"
1.656
373.2
157.0
374.2
457.0
RT 1.798 *MSD1 SPC, [1.781-1.818] of CLQ394869 ES-API, Scan, Frag: 100 "POS"
1.798
157.0
322.0
526.2
RT 1.518 *MSD2 SPC, [1.498-1.559] of CLQ394869 ES-API, Scan, Frag: 100 "NEG"
1.518
524.2
560.2
562.2
525.2
526.2
524.0
560.0
562.0
m/z

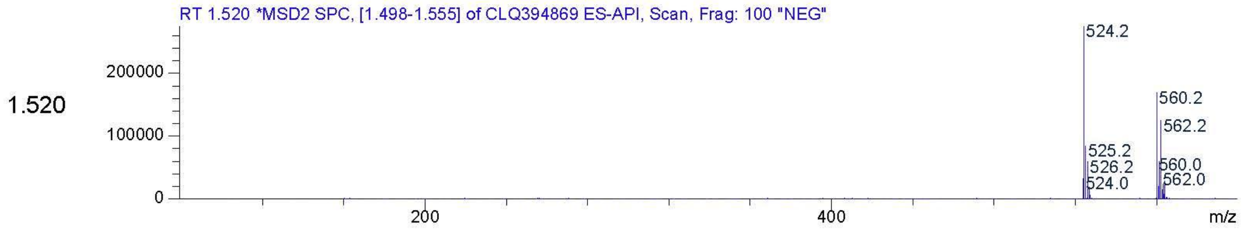

page 3

Figure S20. LC–MS spectrum of *N*-[2-(4-chlorophenyl)-2-morpholin-4-ylethyl]-5-(4-methylphenyl)-2-phenyl[1,3]oxazolo[4,5-*d*]pyrimidin-7-amine (**5**)

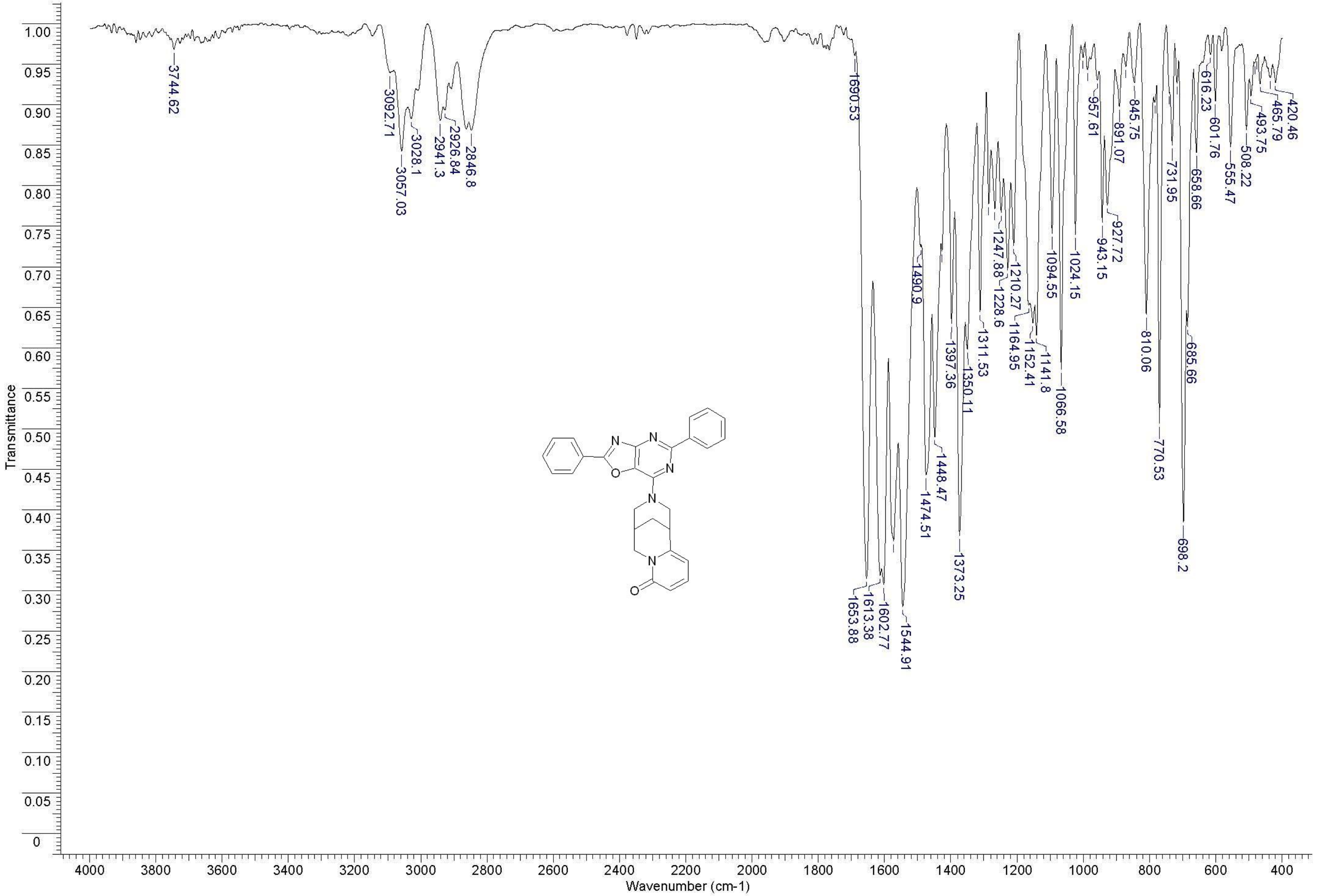

Figure S21. IR spectrum of 11-(2,5-diphenyl-[1,3]oxazolo[4,5-*d*]pyrimidin-7-yl)-7,11-diazatricyclo[7.3.1.02,7]trideca-2,4-dien-6-one (**6**).

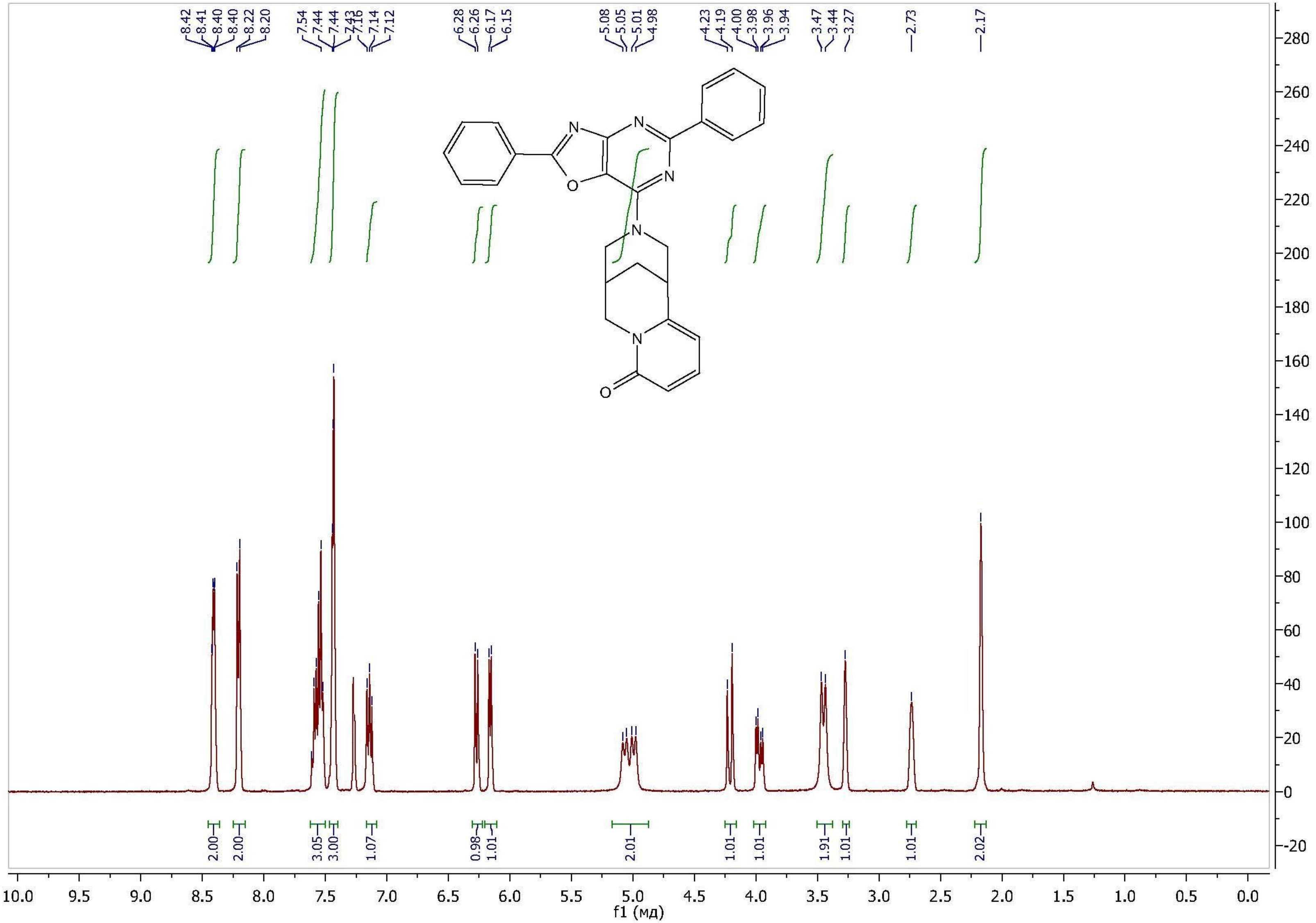

Figure S22. ^{1}H NMR spectrum of 11-(2,5-diphenyl-[1,3]oxazolo[4,5-*d*]pyrimidin-7-yl)-7,11-diazatricyclo[7.3.1.02,7]trideca-2,4-dien-6-one (**6**)

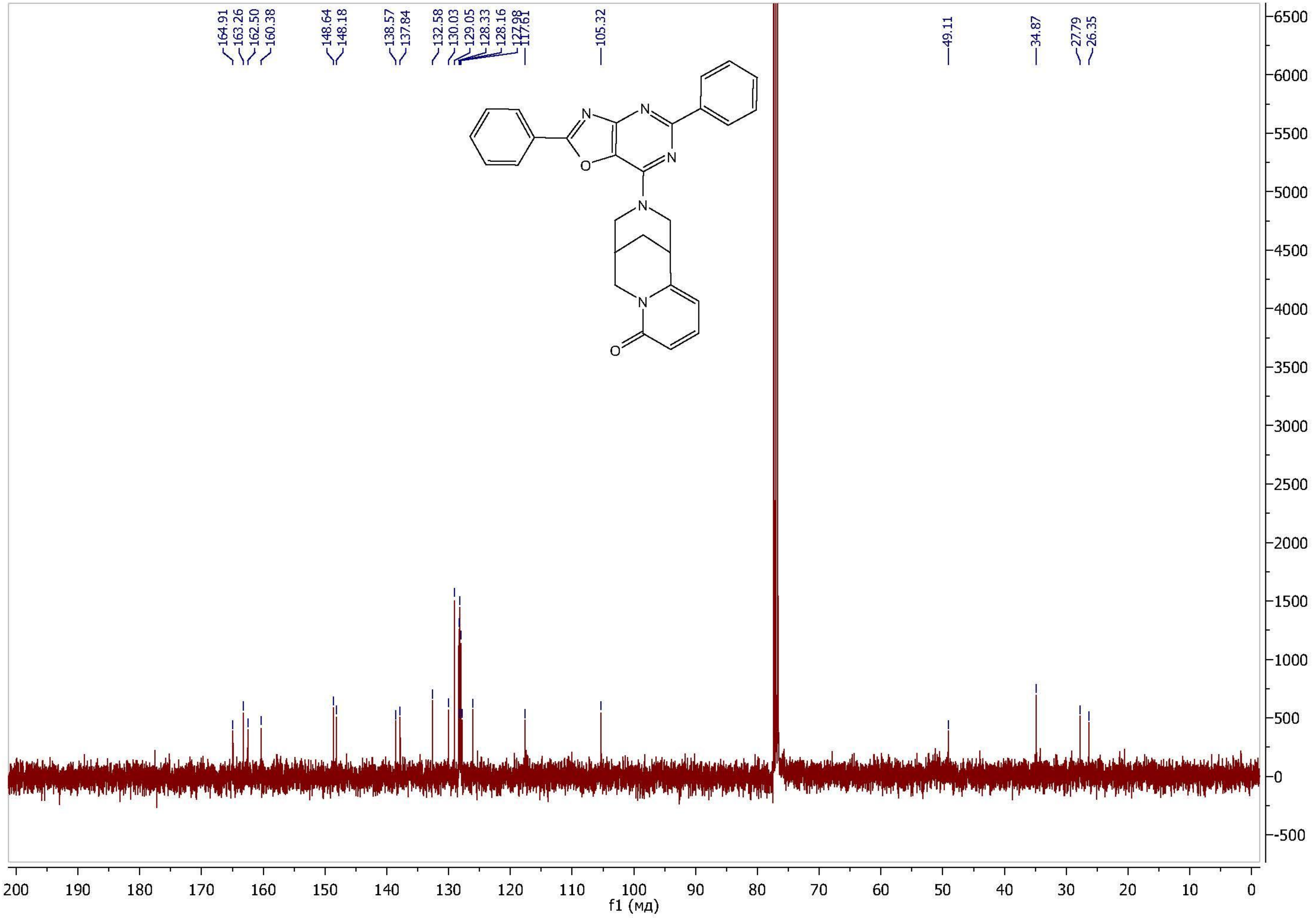

Figure S23. ^{13}C NMR spectrum of 11-(2,5-diphenyl-[1,3]oxazolo[4,5-*d*]pyrimidin-7-yl)-7,11-diazatricyclo[7.3.1.02,7]trideca-2,4-dien-6-one (**6**)

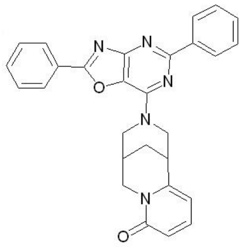

CLQ394875

L970615D
LCMS-37
SUPOR_30.M
16:47 27.08.2025
Product not found
No product

#	RT	DAD1A	DAD1B	MSD1	MSD2	ELSD	MSD1 ions	MSD1 rt	MSD2 ions	MSD2 rt	Info
1	1.326	2.8%	---	---	---	---	---	---	---	---	
2	1.434	97.2%	100.0%	100.0%	100.0%	100.0%	462.2(100)	1.444	496.0(81),506.0(12),248.6(7)	1.448	

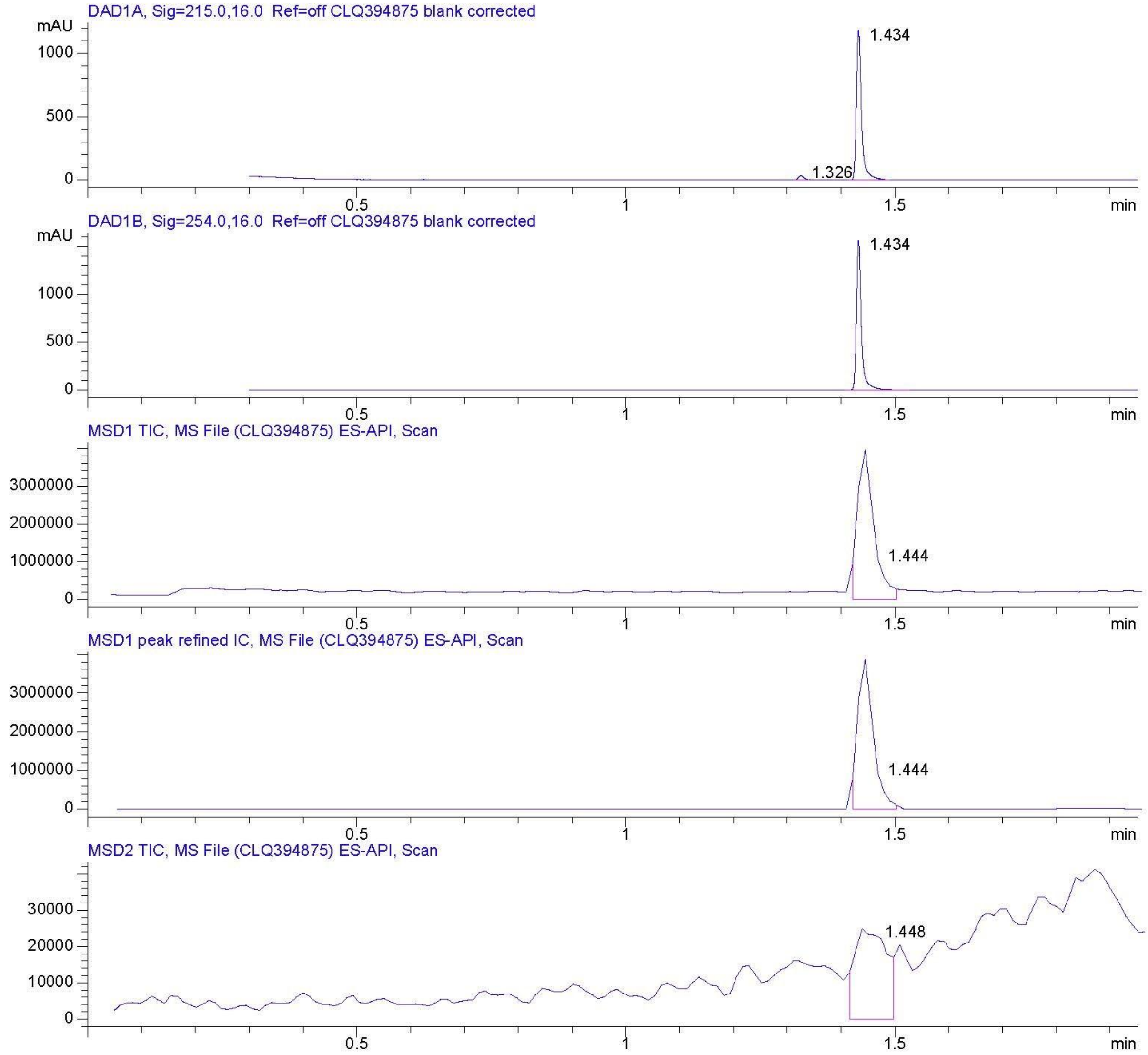

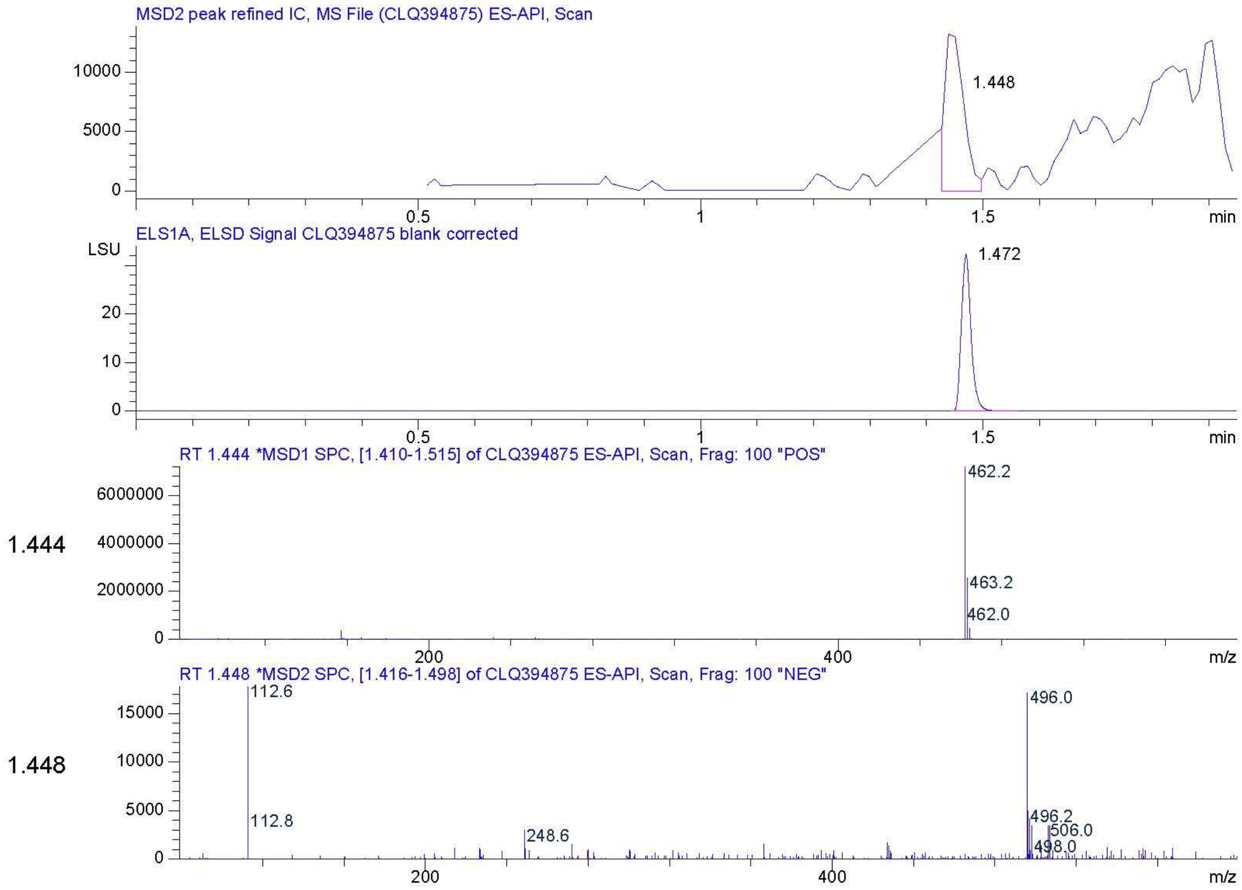

page 2

Figure S24. LC–MS spectrum of 11-(2,5-diphenyl-[1,3]oxazolo[4,5-*d*]pyrimidin-7-yl)-7,11-diazatricyclo[7.3.1.02,7]trideca-2,4-dien-6-one (**6**)

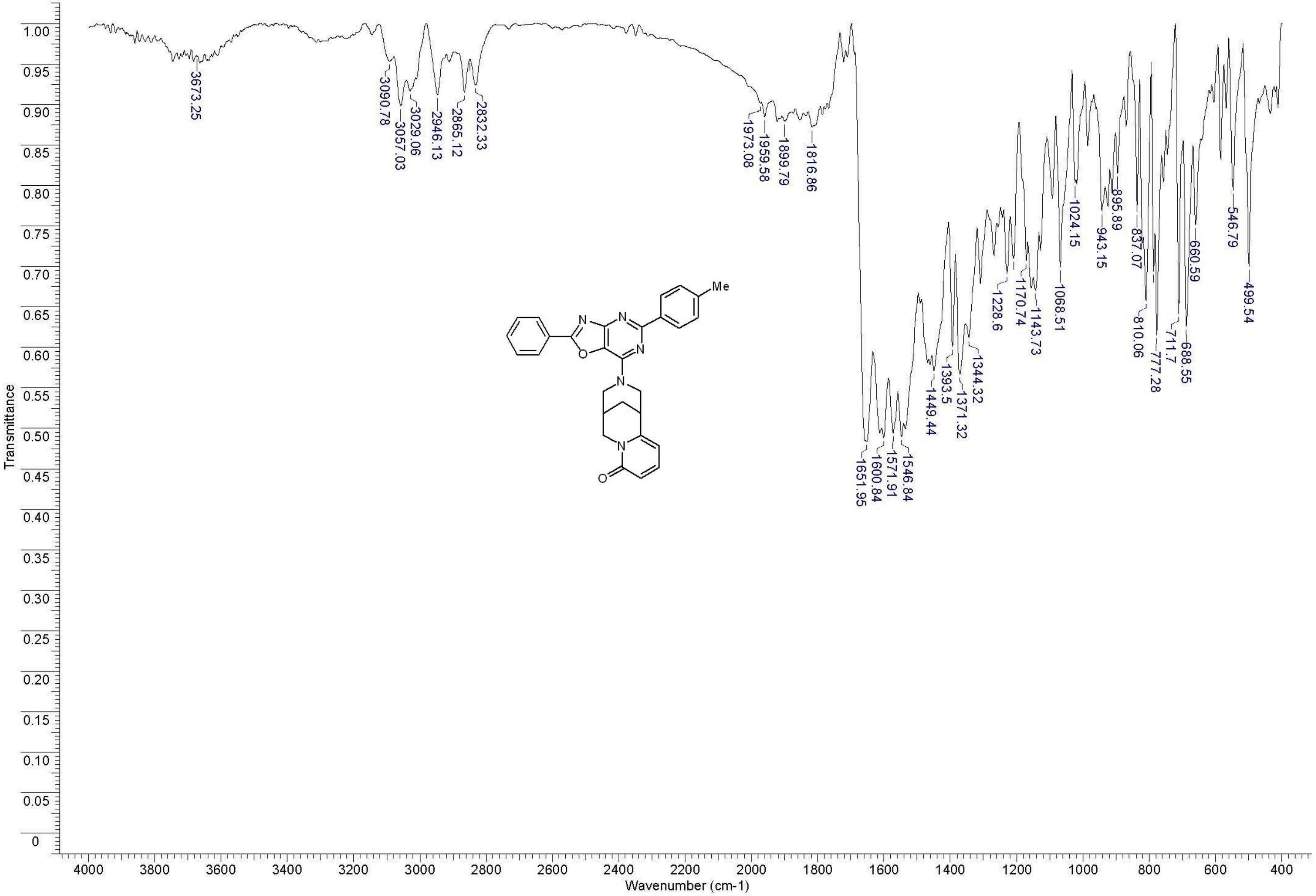

Figure S25. IR spectrum of 11-[5-(4-methylphenyl)-2-phenyl-[1,3]oxazolo[4,5-*d*]pyrimidin-7-yl]-7,11-diazatricyclo[7.3.1.02,7]trideca-2,4-dien-6-one (**7**)

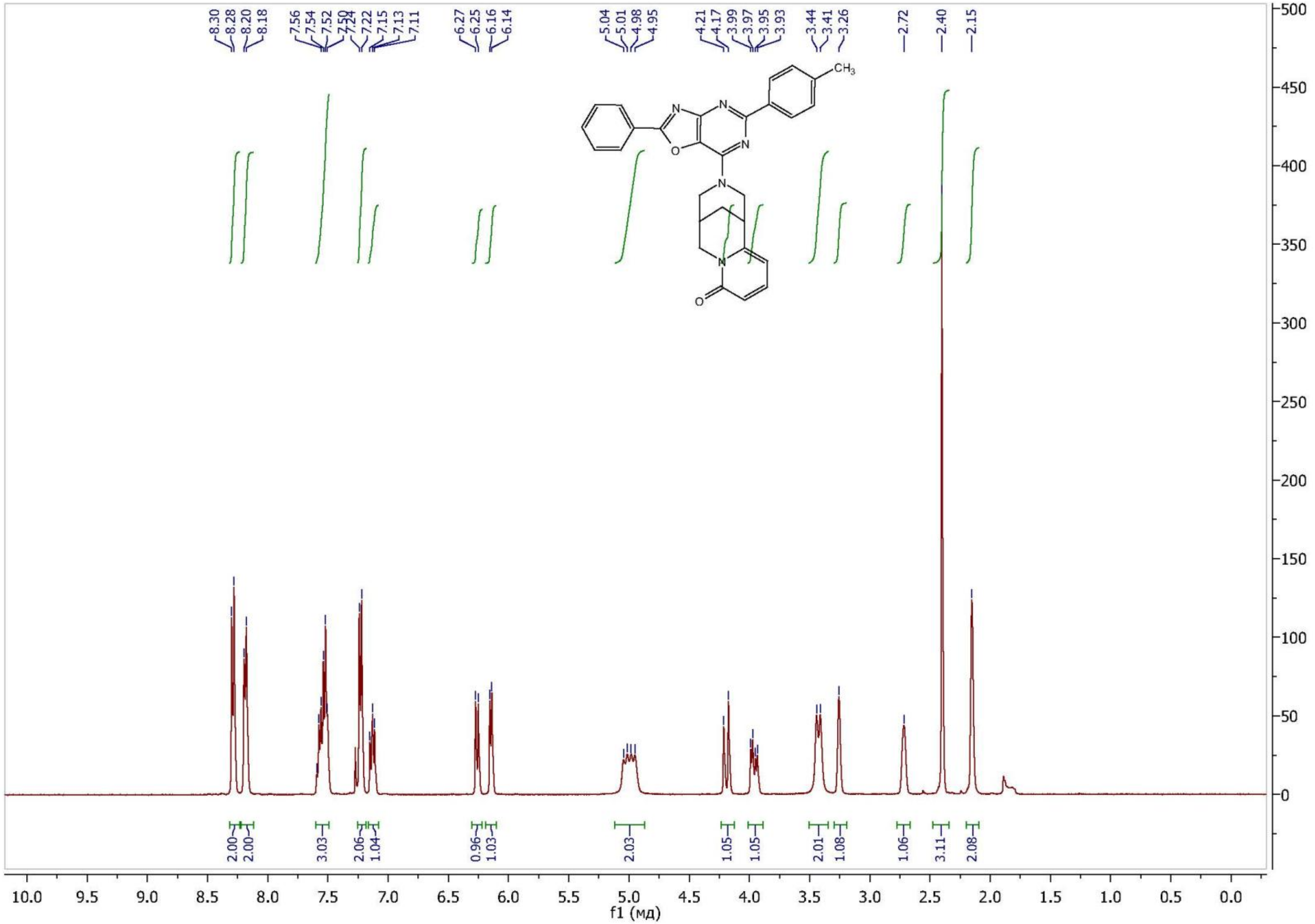

Figure S26. ^{1}H NMR spectrum of 11-[5-(4-methylphenyl)-2-phenyl-[1,3]oxazolo[4,5-*d*]pyrimidin-7-yl]-7,11-diazatricyclo[7.3.1.02,7]trideca-2,4-dien-6-one (**7**)

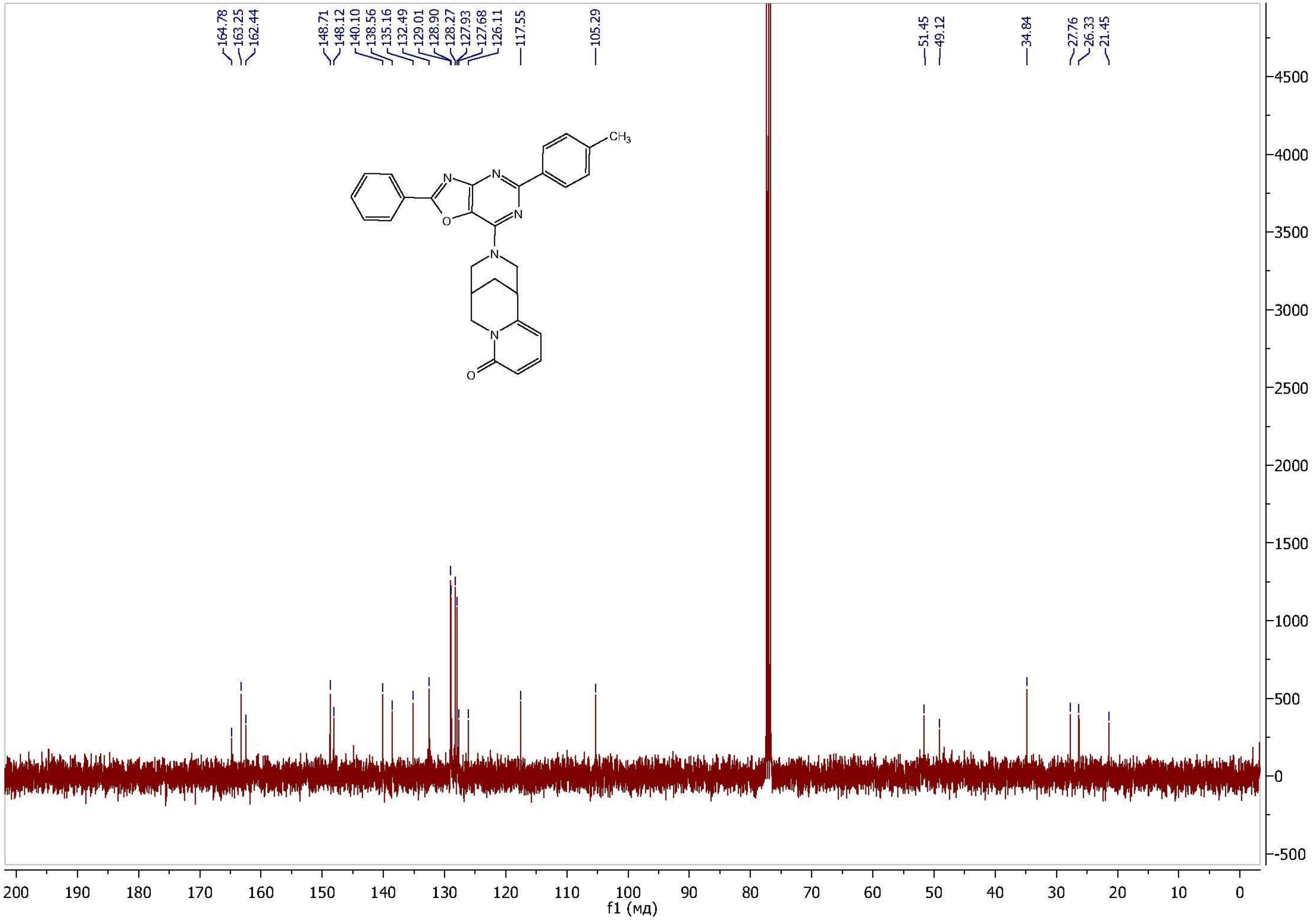

Figure S27. ^{13}C NMR spectrum of 11-[5-(4-methylphenyl)-2-phenyl-[1,3]oxazolo[4,5-*d*]pyrimidin-7-yl]-7,11-diazatricyclo[7.3.1.02,7]trideca-2,4-dien-6-one (**7**)

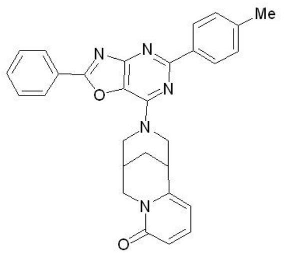

CLQ394876

L970615D
LCMS-37
SUPOR_30.M
16:50 27.08.2025
Product not found
No product

#	RT	DAD1A	DAD1B	MSD1	MSD2	ELSD	MSD1 ions	MSD1 rt	MSD2 ions	MSD2 rt	Info
1	1.496	100.0%	100.0%	100.0%	27.6%	100.0%	476.2(100)	1.506	510.2(100)	1.504	
2	1.893	---	---	---	72.4%	---	---	---	489.2(58),461.2(10),536.0(8)	1.901	

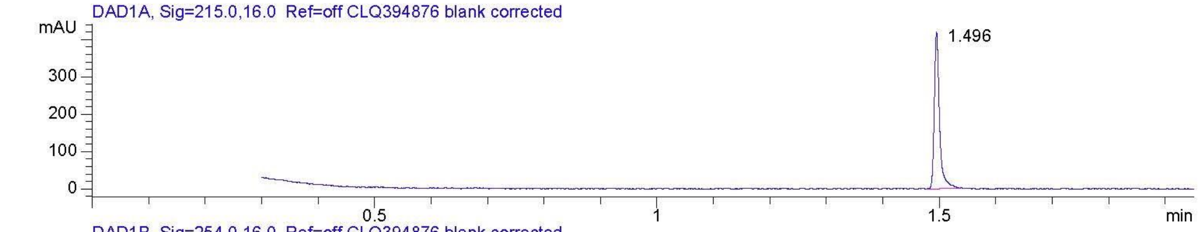

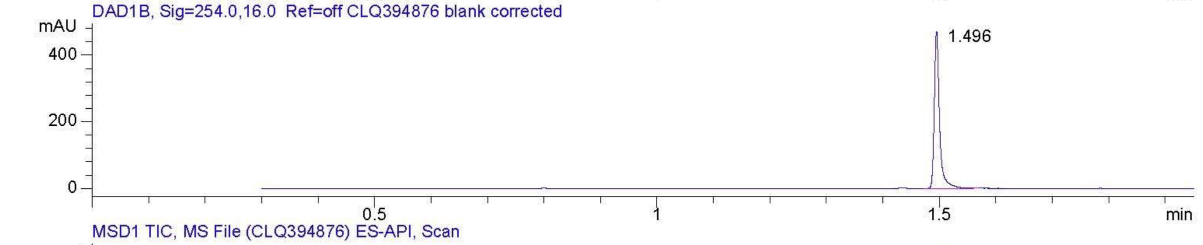

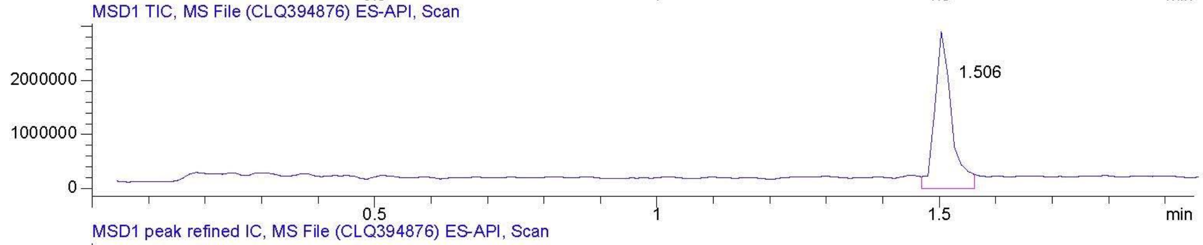

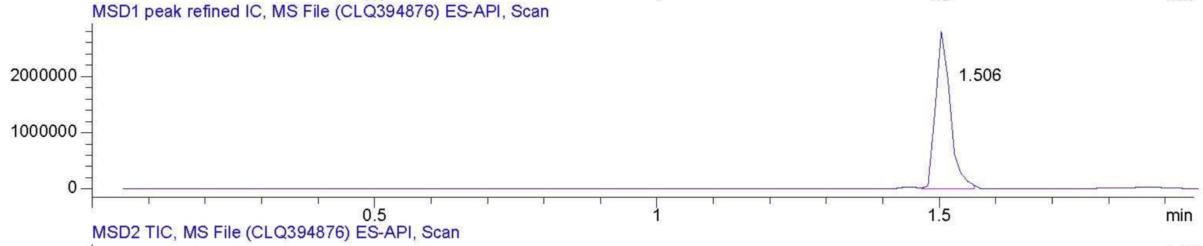

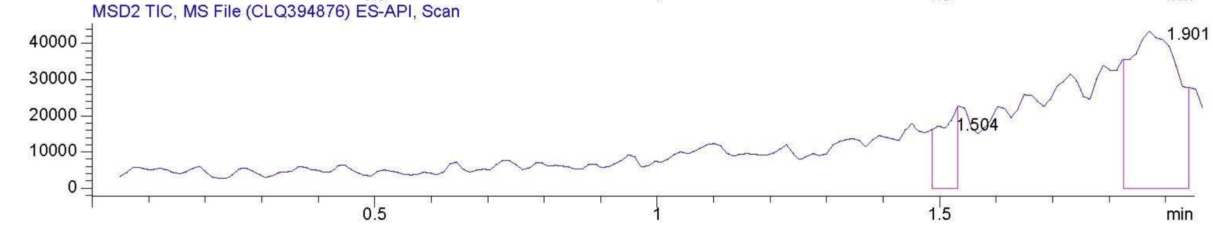

ELSD shift 229.1 | +1000 / -1000
MSD shift 488 | +420 / -420
f:\ABCH\2025\37\8\08_27\L970615D\002-NV-B.D
f:\ABCH\2025\37\8\08_27\L970615D\004-D4F-A3-CLQ394876.D
https://spectra.enamine.net/alpha?instrument=LCMS-37&acquiredDate=1756302652000&barcode=CLQ394876

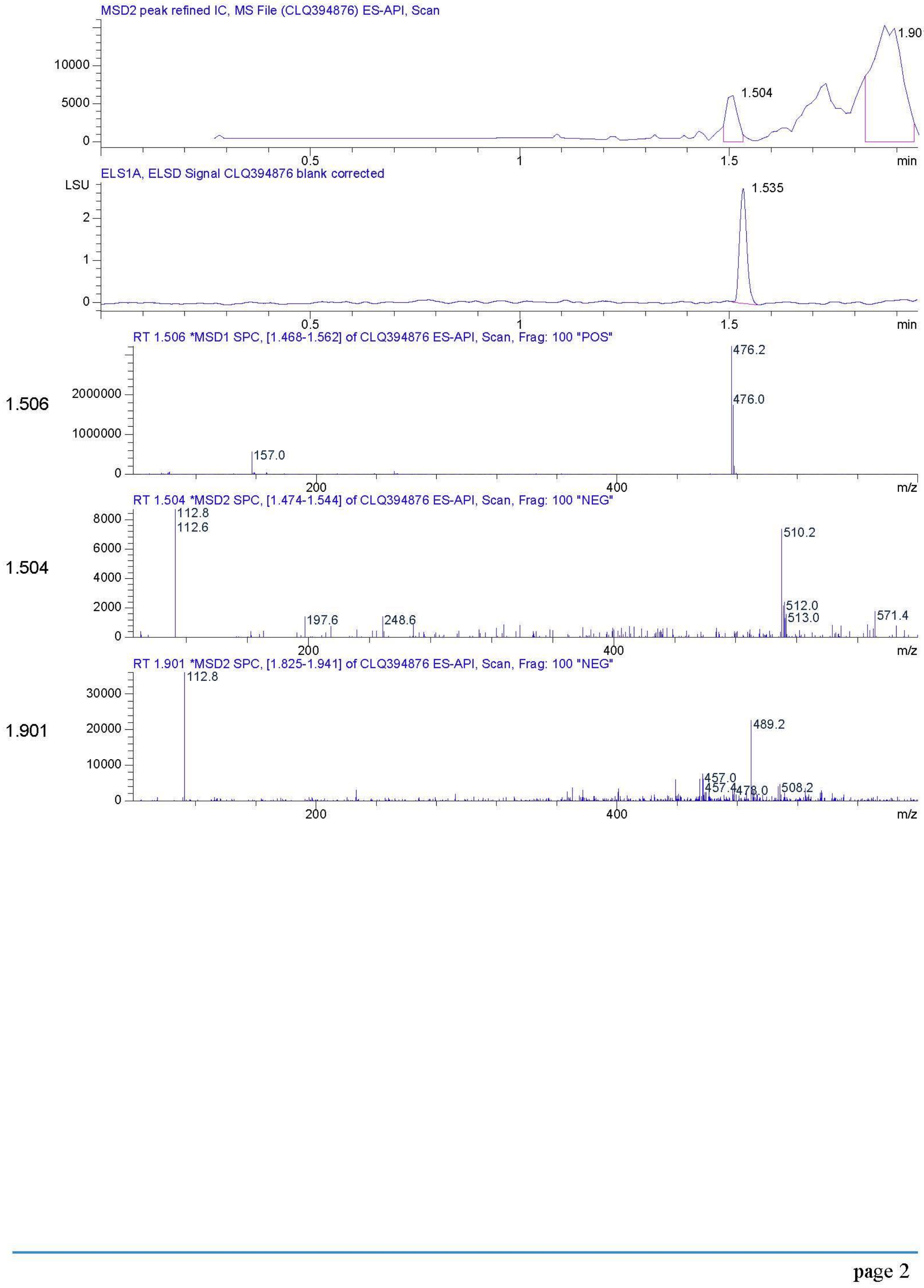

Figure S28. LC–MS spectrum of 11-[5-(4-methylphenyl)-2-phenyl-[1,3]oxazolo[4,5-*d*]pyrimidin-7-yl]-7,11-diazatricyclo[7.3.1.02,7]trideca-2,4-dien-6-one (**7**)

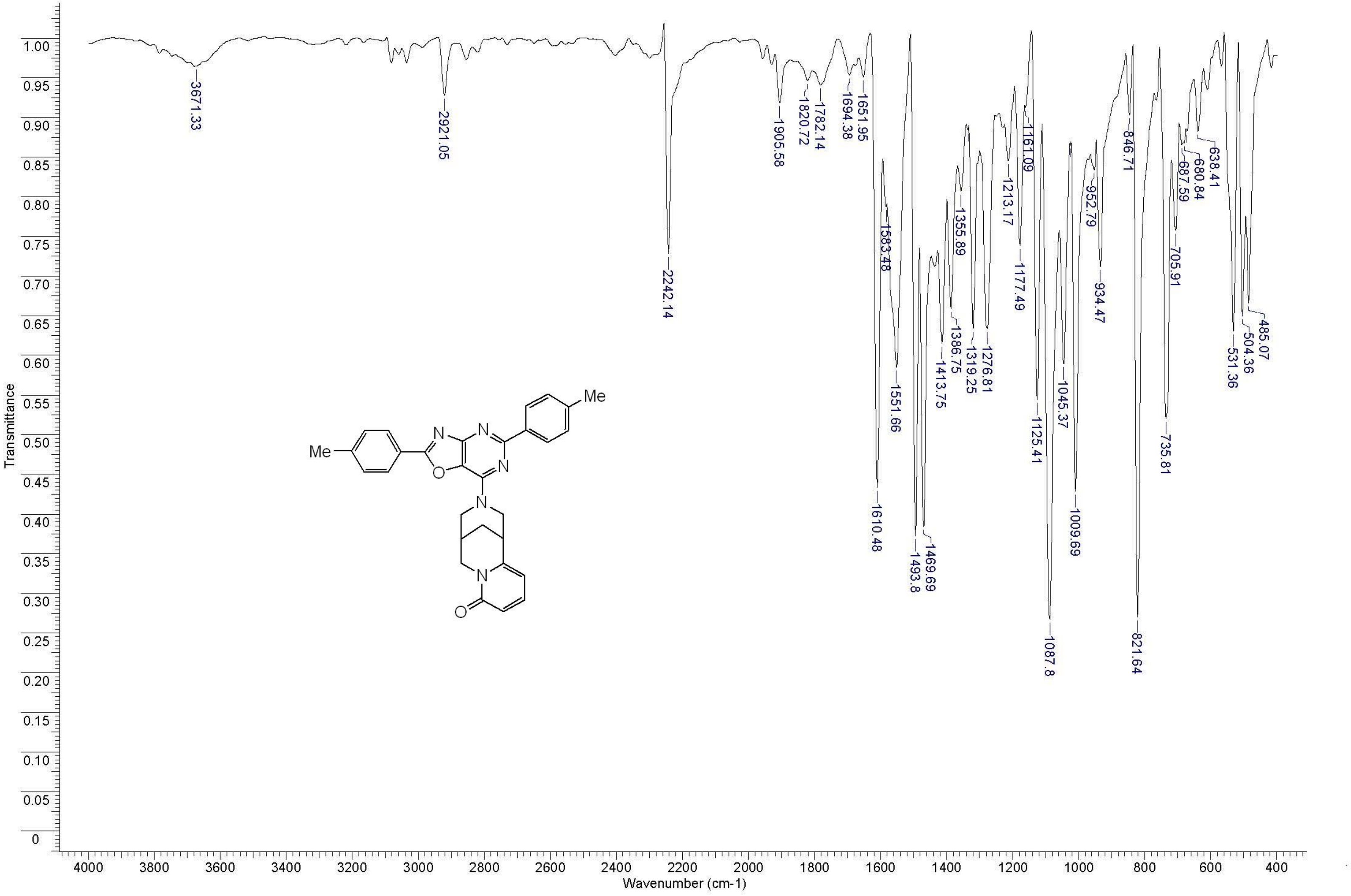

Figure S29. IR spectrum of 11-[2,5-bis(4-methylphenyl)-[1,3]oxazolo[4,5-*d*]pyrimidin-7-yl]-7,11-diazatricyclo[7.3.1.02,7]trideca-2,4-dien-6-one (**8**)

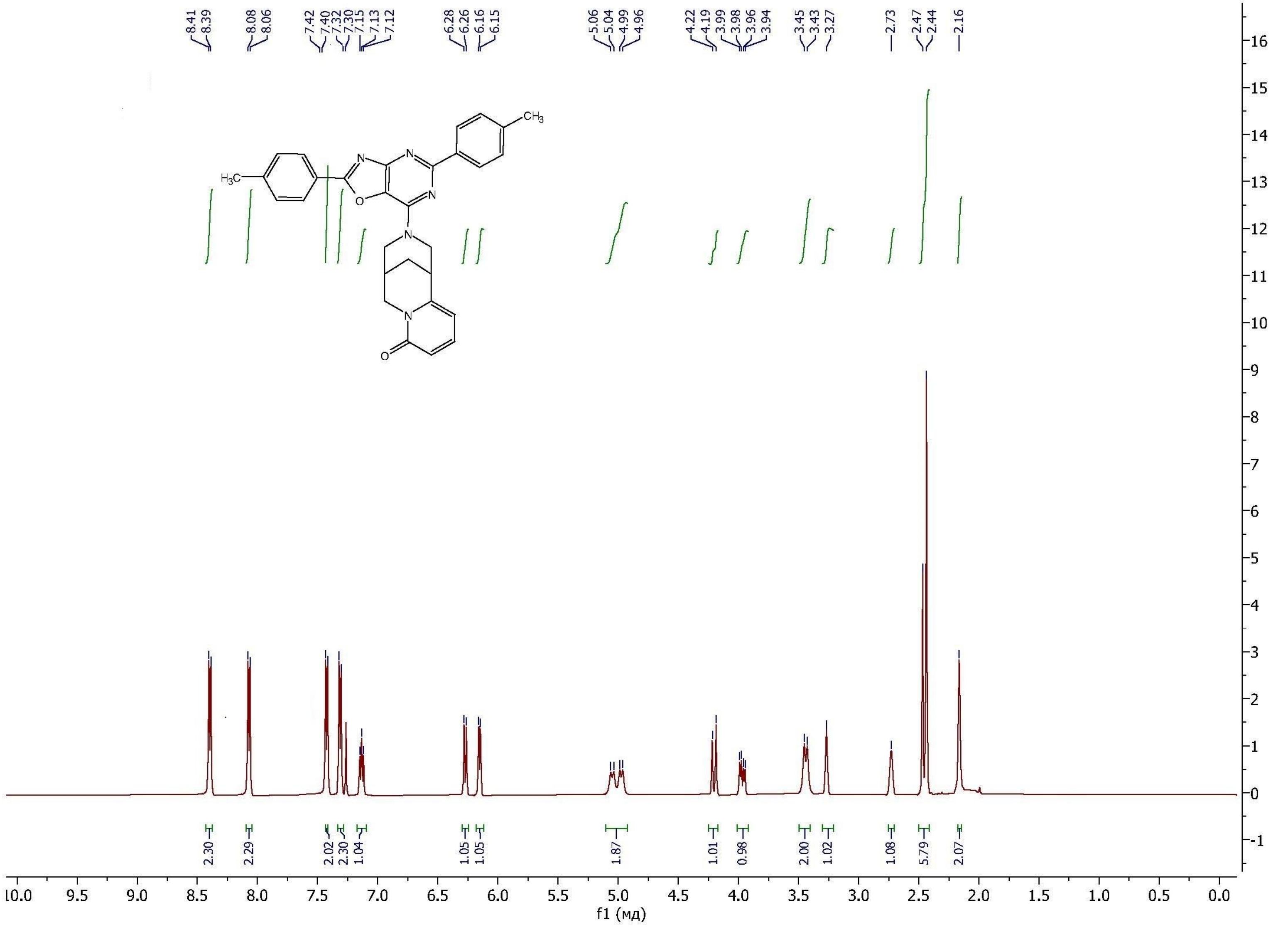

Figure S30. ^{1}H NMR spectrum of 11-[2,5-bis(4-methylphenyl)-[1,3]oxazolo[4,5-*d*]pyrimidin-7-yl]-7,11-diazatricyclo[7.3.1.02,7]trideca-2,4-dien-6-one (**8**)

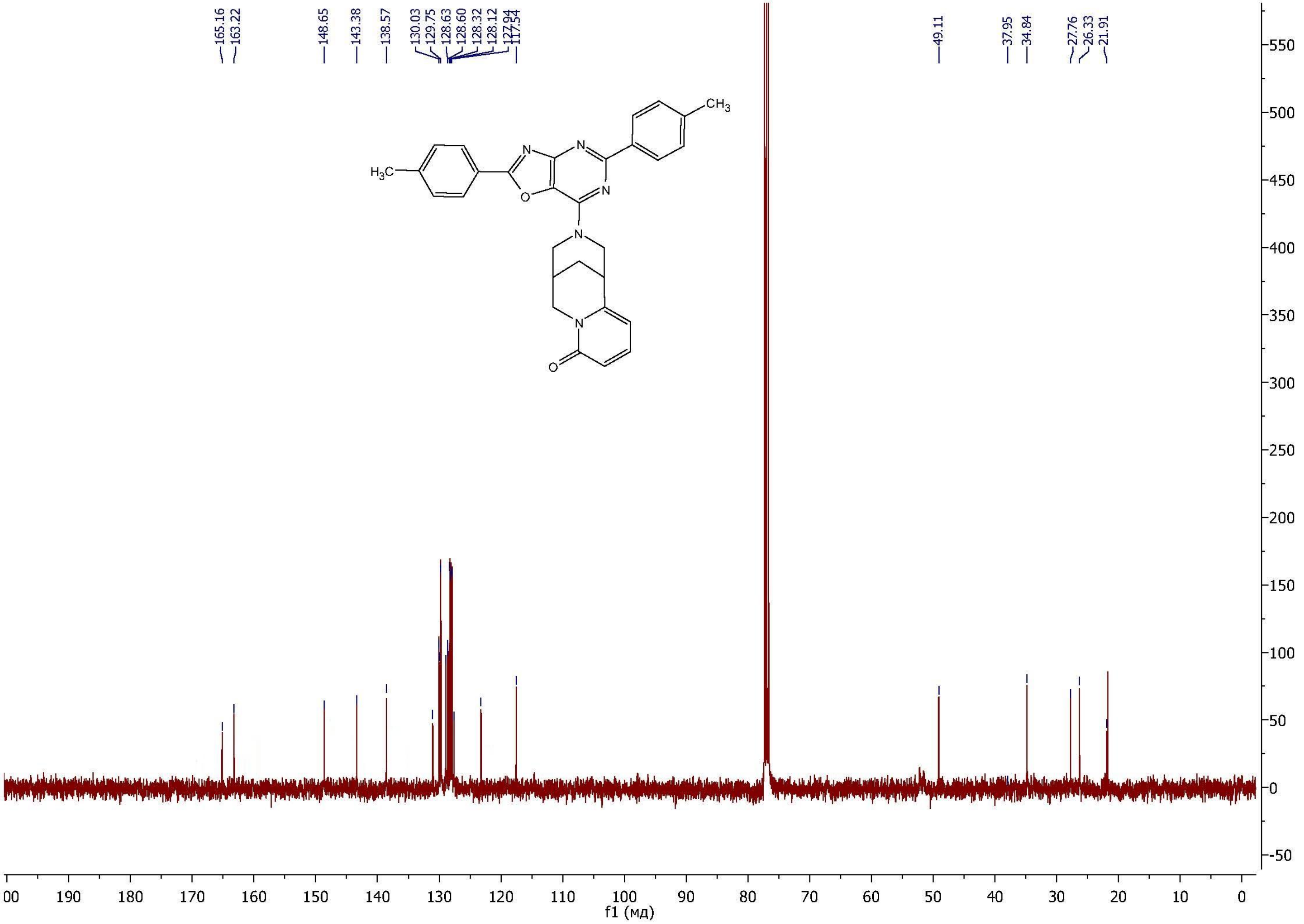

Figure S31. ^{13}C NMR spectrum of 11-[2,5-bis(4-methylphenyl)-[1,3]oxazolo[4,5-*d*]pyrimidin-7-yl]-7,11-diazatricyclo[7.3.1.02,7]trideca-2,4-dien-6-one (**8**)

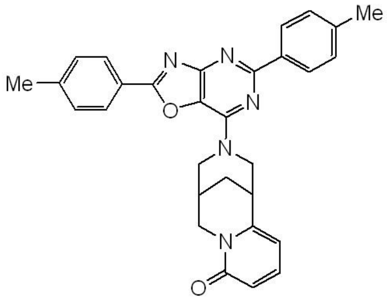

CLQ394878

L970615D
LCMS-37
SUPOR_30.M
16:56 27.08.2025
Product not found
No product

#	RT	DAD1A	DAD1B	MSD1	MSD2	ELSD	MSD1 ions	MSD1 rt	MSD2 ions	MSD2 rt	Info
1	1.559	95.9%	96.7%	89.2%	4.9%	89.6%	490.2(100)	1.566	248.6(57),526.0(43)	1.567	
2	1.847	4.1%	3.3%	10.8%	67.7%	10.4%	336.0(100)	1.857	457.2(29),489.0(12),455.2(8)	1.856	
3	1.901	---	---	---	27.4%	---	---	---	457.2(29),441.2(26),377.2(18)	1.909	

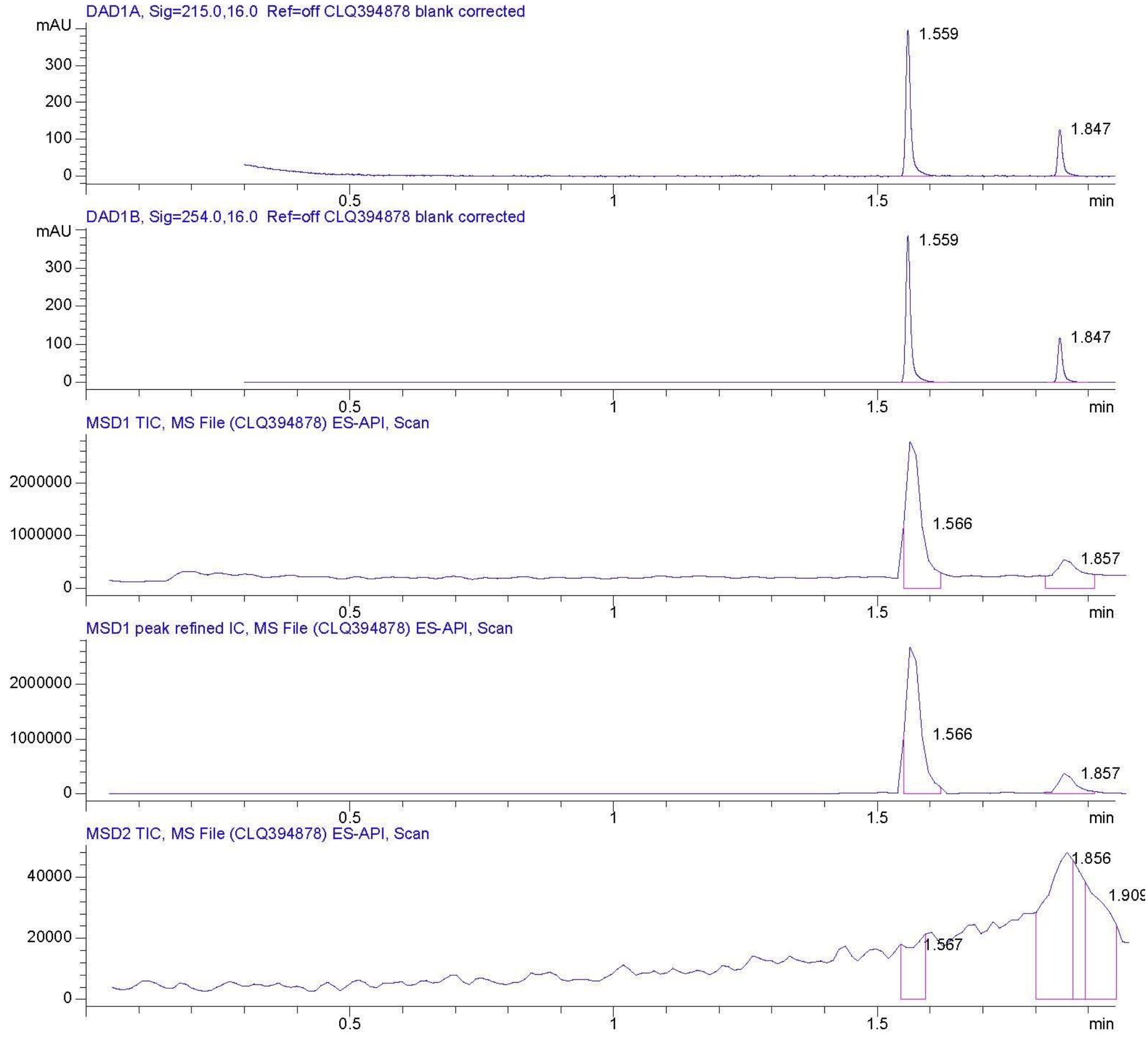

ELSD shift 2288 | +1000 / -1000
MSD shift 483 | +420 / -420
f:\ABCH\2025\37\08\08_27\L970615D\002-NV-B.D
f:\ABCH\2025\37\08\08_27\L970615D\006-D4F-A4-CLQ394878.D
https://spectra.enamine.net/alpha?instrument=LCMS-37&acquiredDate=1756303000000&barcode=CLQ394878

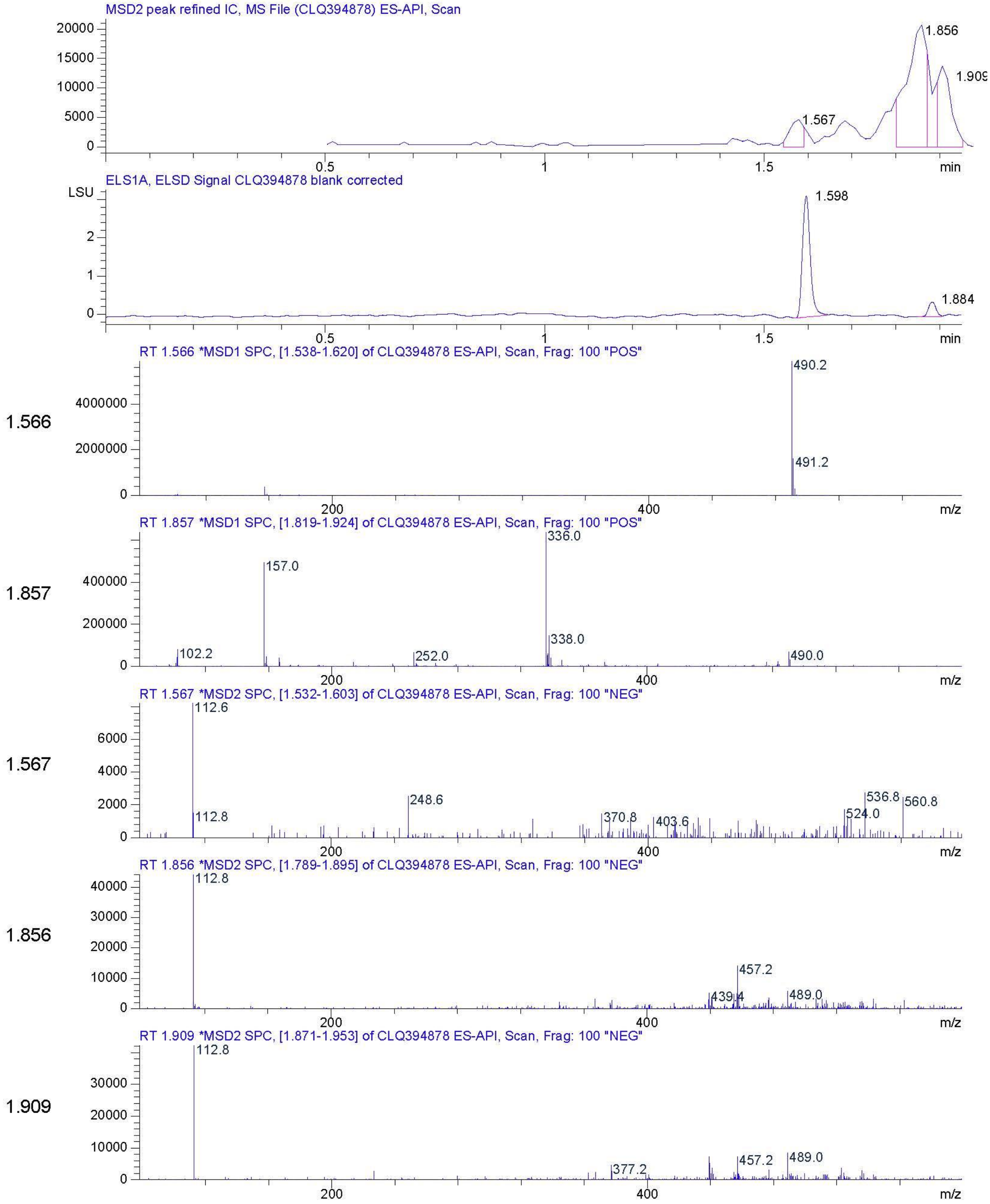

page 2

Figure S32. LC–MS spectrum of 11-[2,5-bis(4-methylphenyl)-[1,3]oxazolo[4,5-*d*]pyrimidin-7-yl]-7,11-diazatricyclo[7.3.1.02,7]trideca-2,4-dien-6-one (**8**)

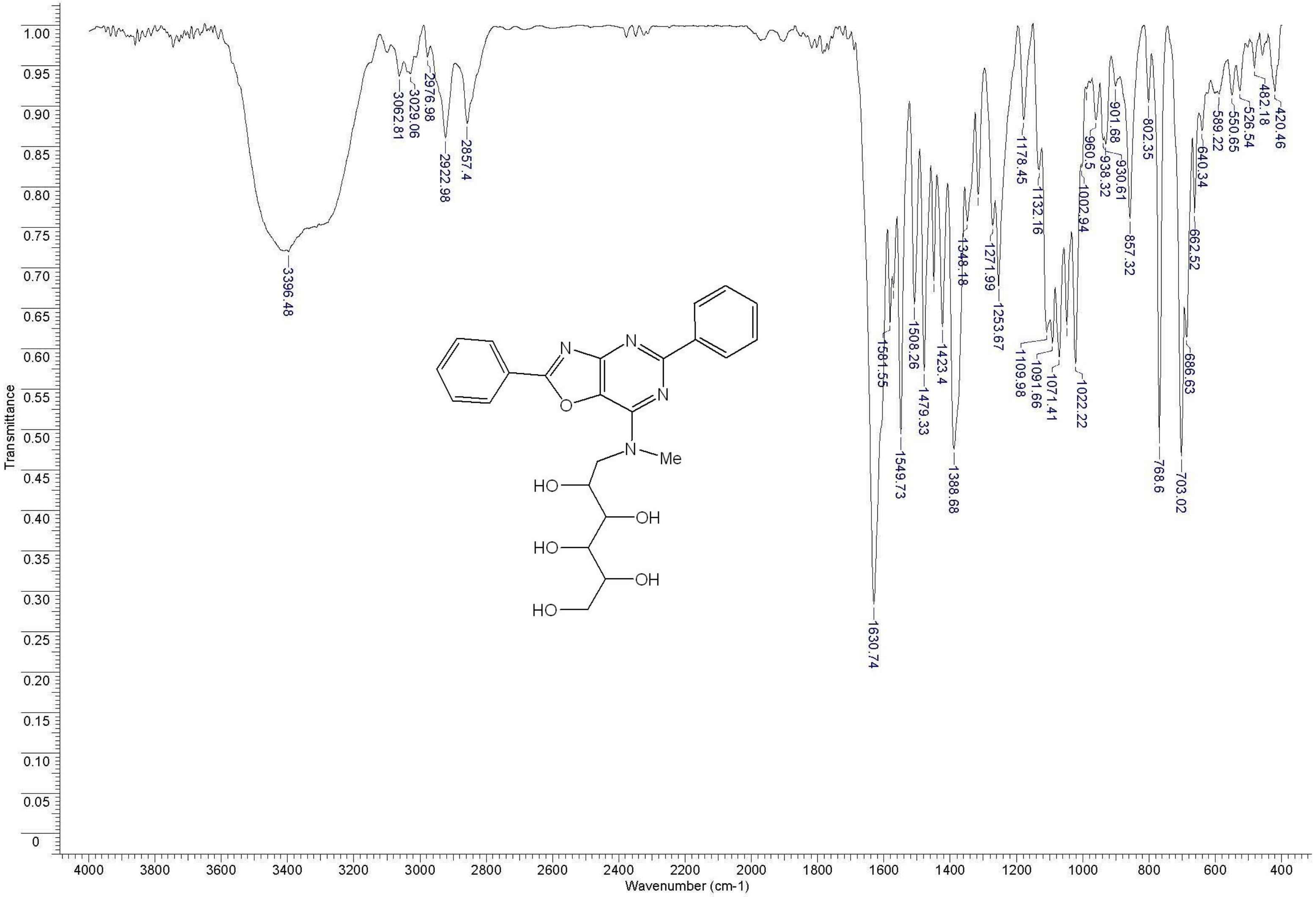

Figure S33. IR spectrum of 6-[(2,5-diphenyl-[1,3]oxazolo[4,5-*d*]pyrimidin-7-yl)-methylamino]hexane-1,2,3,4,5-pentol (**9**)

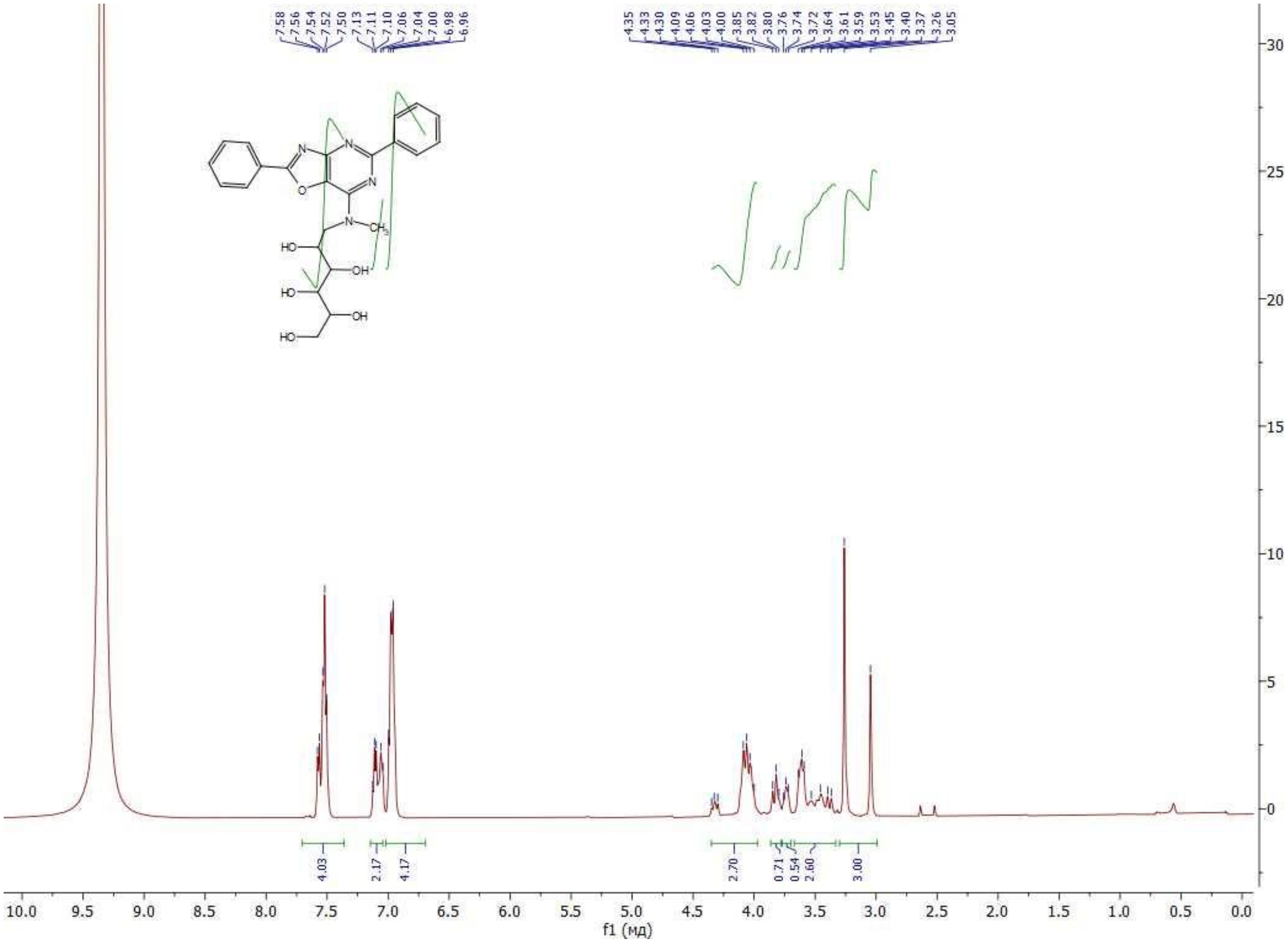

Figure S34. ^{1}H NMR spectrum of 6-[(2,5-diphenyl-[1,3]oxazolo[4,5-*d*]pyrimidin-7-yl)-methylamino]hexane-1,2,3,4,5-pentol (**9**)

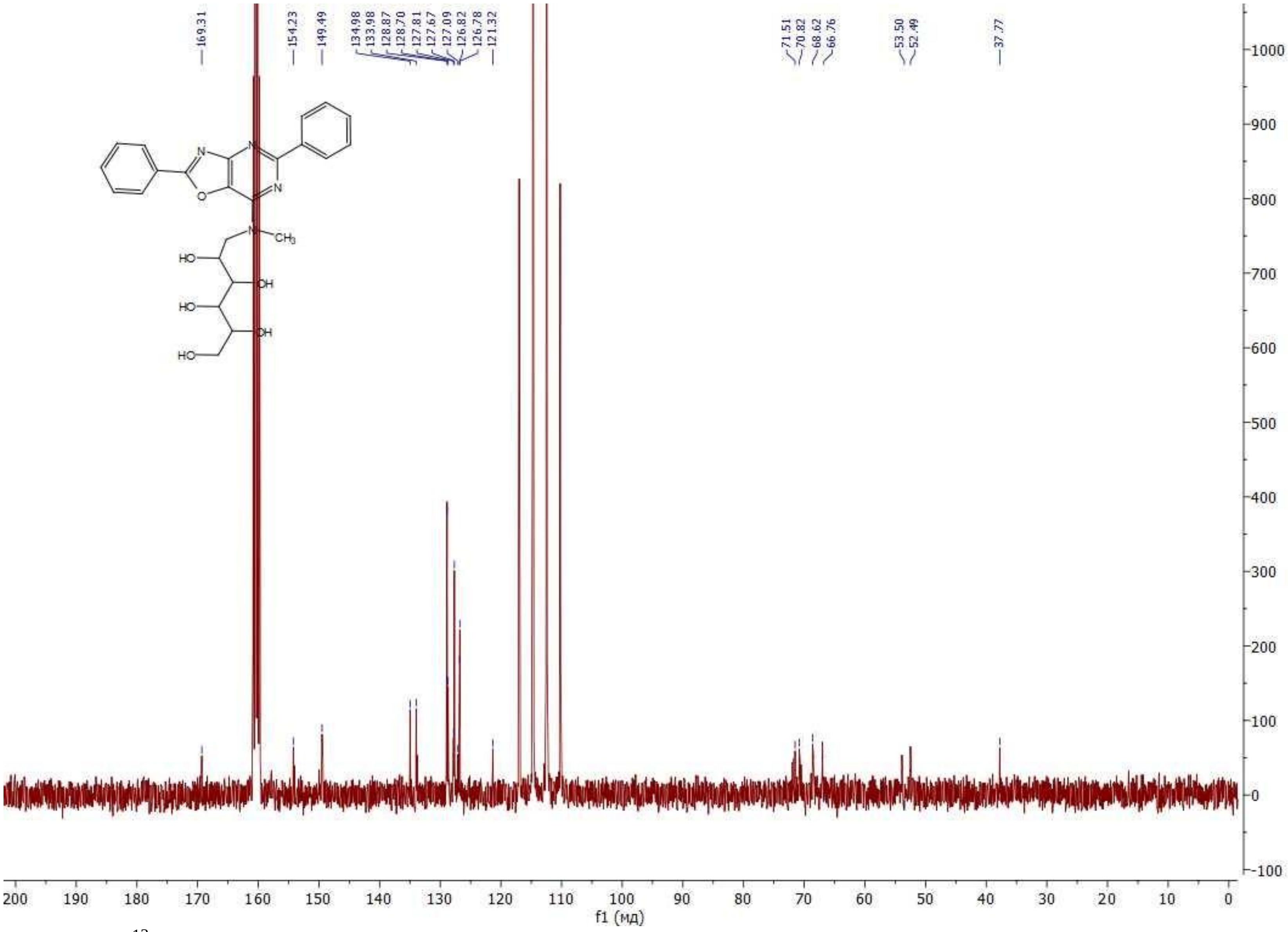

Figure S35. ^{13}C NMR spectrum of 6-[(2,5-diphenyl-[1,3]oxazolo[4,5-*d*]pyrimidin-7-yl)-methylamino]hexane-1,2,3,4,5-pentol (**9**)

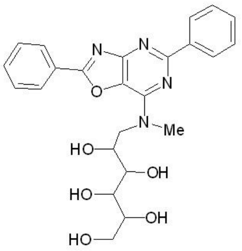

CLQ394879

L970615D
LCMS-37
SUPOR_30.M
17:02 27.08.2025
Product not found
No product

#	RT	DAD1A	DAD1B	MSD1	MSD2	ELSD	MSD1 ions	MSD1 rt	MSD2 ions	MSD2 rt	Info
1	1.154	100.0%	100.0%	100.0%	100.0%	100.0%	467.2(100)	1.165	465.2(40),501.2(36),579.2(12)	1.163	

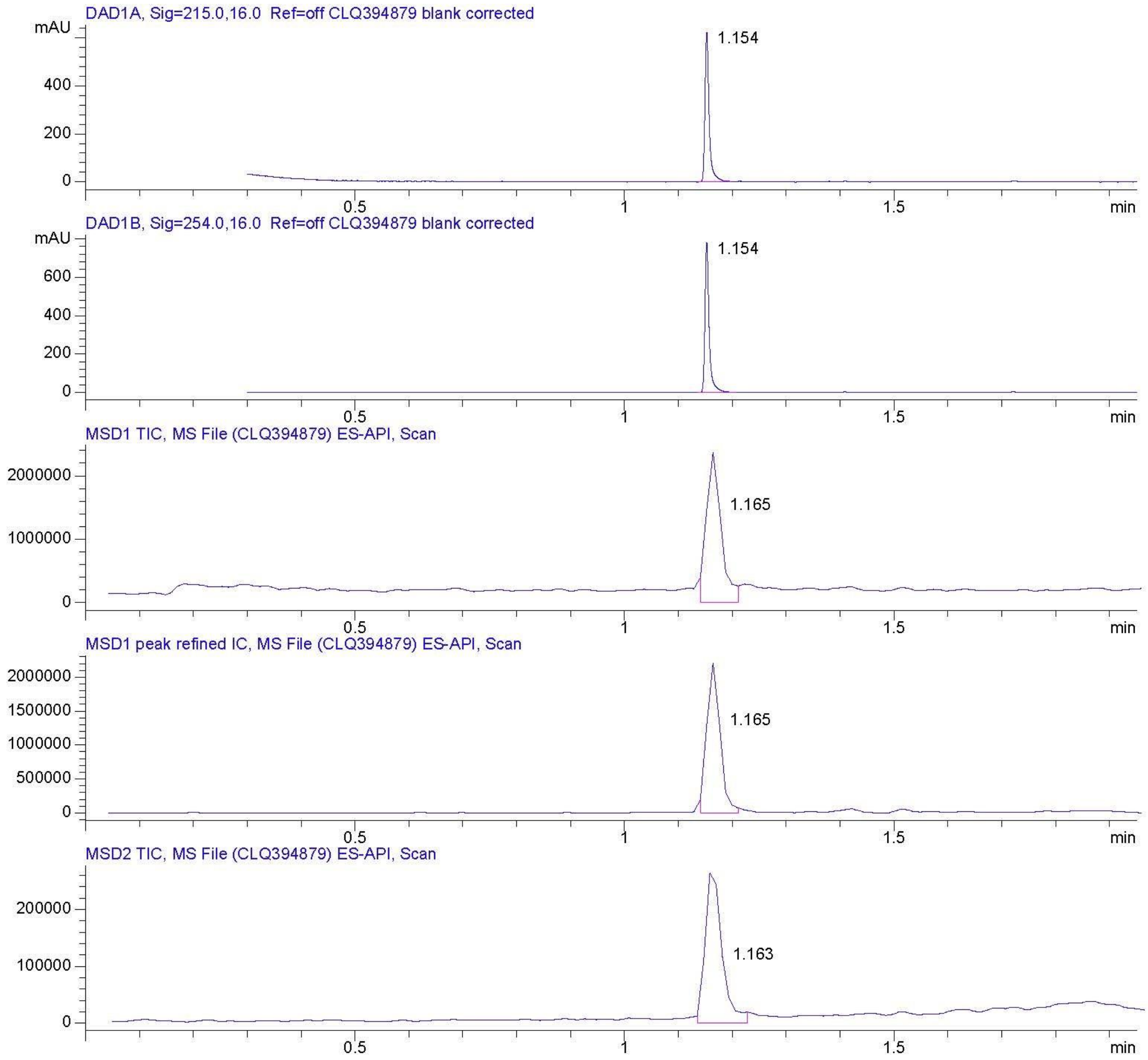

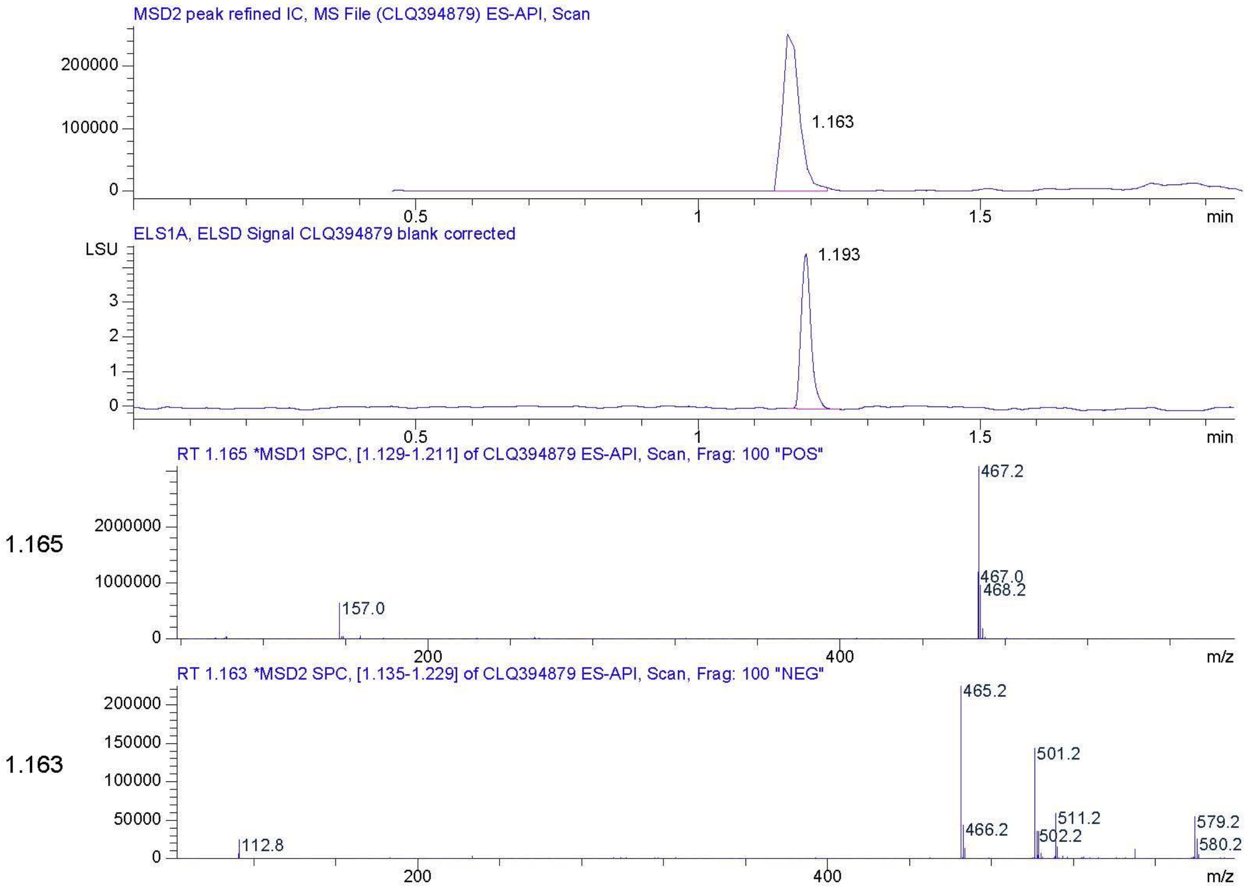

Figure S36. LC–MS spectrum of 6-[(2,5-diphenyl-[1,3]oxazolo[4,5-*d*]pyrimidin-7-yl)-methylamino]hexane-1,2,3,4,5-pentol (**9**)
